# Supplementary material for: Fast-exchanging spirocyclic rhodamine probes for aptamer-based super-resolution RNA imaging
Source: Nat Commun. 2023 Jun 30;14:3879. doi: 10.1038/s41467-023-39611-1 (PMC10313827; doi:10.1038/s41467-023-39611-1)
Supplement: Supplementary file 1 — Supplementary Information [file 41467_2023_39611_MOESM1_ESM.pdf]

Supplementary Information

Fast-exchanging spirocyclic rhodamine probes for aptamer-based super-resolution RNA imaging

Daniel Englert[1], Eva-Maria Burger[1], Franziska Grün[1], Mrigank S. Verma[2], Jens Lackner[2], Marko Lampe[3], Bastian Bühler[1], Janin Schokolowski[1], G. Ulrich Nienhaus[2,4,5,6]*, Andres Jäschke[1]* and Murat Sunbul[1]*

* Corresponding authors: uli@uiuc.edu , jaeschke@uni-hd.de , msunbul@uni-heidelberg.de

[1]Institute of Pharmacy and Molecular Biotechnology (IPMB), Heidelberg University, Heidelberg, Germany.

[2]Institute of Applied Physics (APH), Karlsruhe Institute of Technology (KIT), Karlsruhe, Germany.

[3]Advanced Light Microscopy Facility, European Molecular Biology Laboratory, Heidelberg, Germany.

[4]Institute of Nanotechnology (INT), Karlsruhe Institute of Technology (KIT), Eggenstein-Leopoldshafen, Germany.

[5]Institute of Biological and Chemical Systems (IBCS), Karlsruhe Institute of Technology (KIT), Eggenstein-Leopoldshafen, Germany.

[6]Department of Physics, University of Illinois at Urbana–Champaign, Urbana, IL, USA.

Supplementary figures

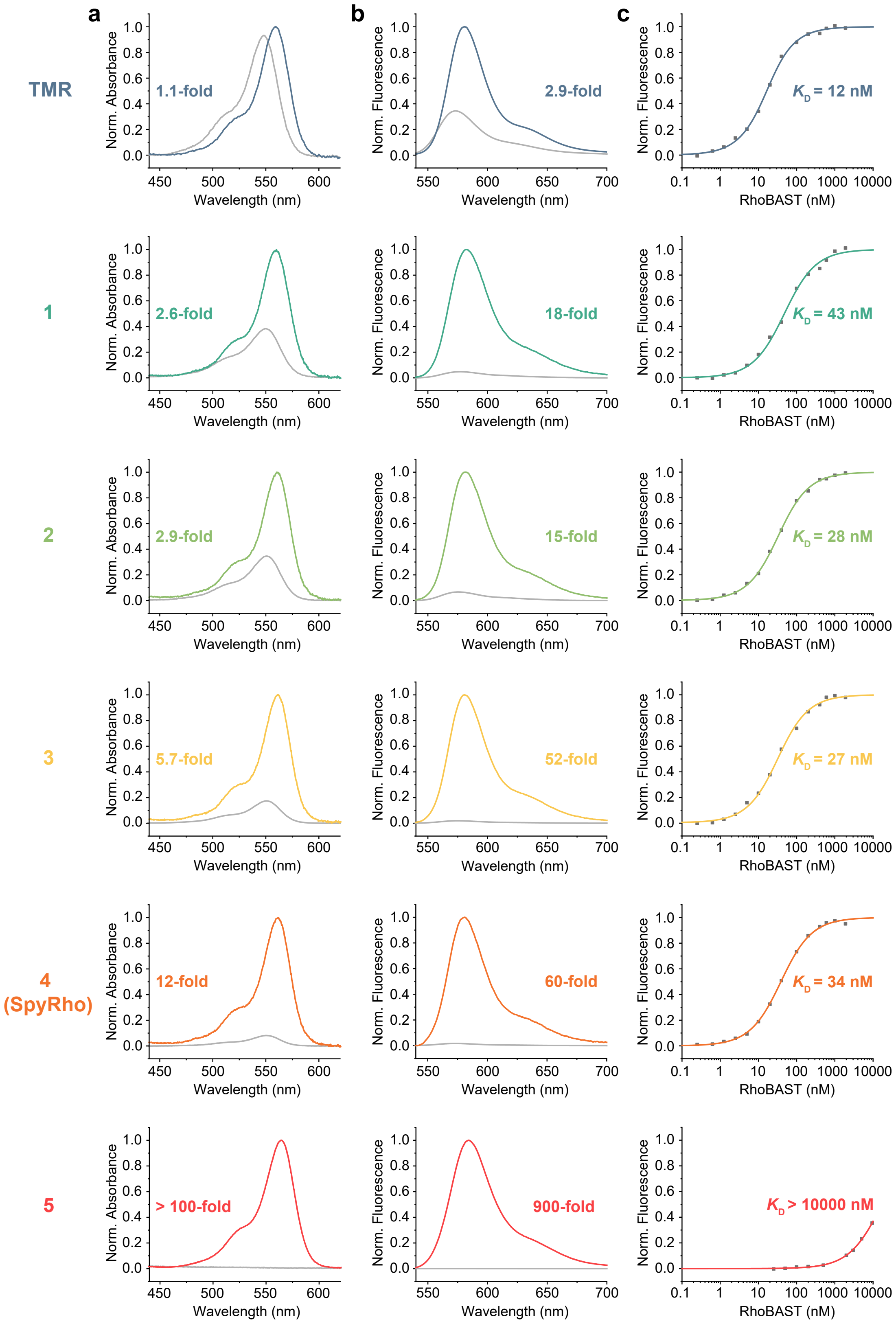

Supplementary Figure 1. Properties of TMR and amide-substituted TMR derivatives. a) Normalized absorption spectra of rhodamines in the presence (colored lines) and absence (grey lines) of RhoBAST. Given absorption increase corresponds to the ratio of the excitation maxima of the bound and unbound probe. **b)** Normalized fluorescence emission spectra of rhodamines in the presence (colored lines) and absence (grey lines) of RhoBAST. Absorbance and fluorescence measurements were performed in ASB at 25 °C using 1 µM dye and 5 µM RhoBAST. **c)** Isotherms of rhodamines binding to RhoBAST. Measurements were performed in ASB supplemented with 0.05% Tween20 at 25 °C using 10 nM probe (50 nM for **5**).

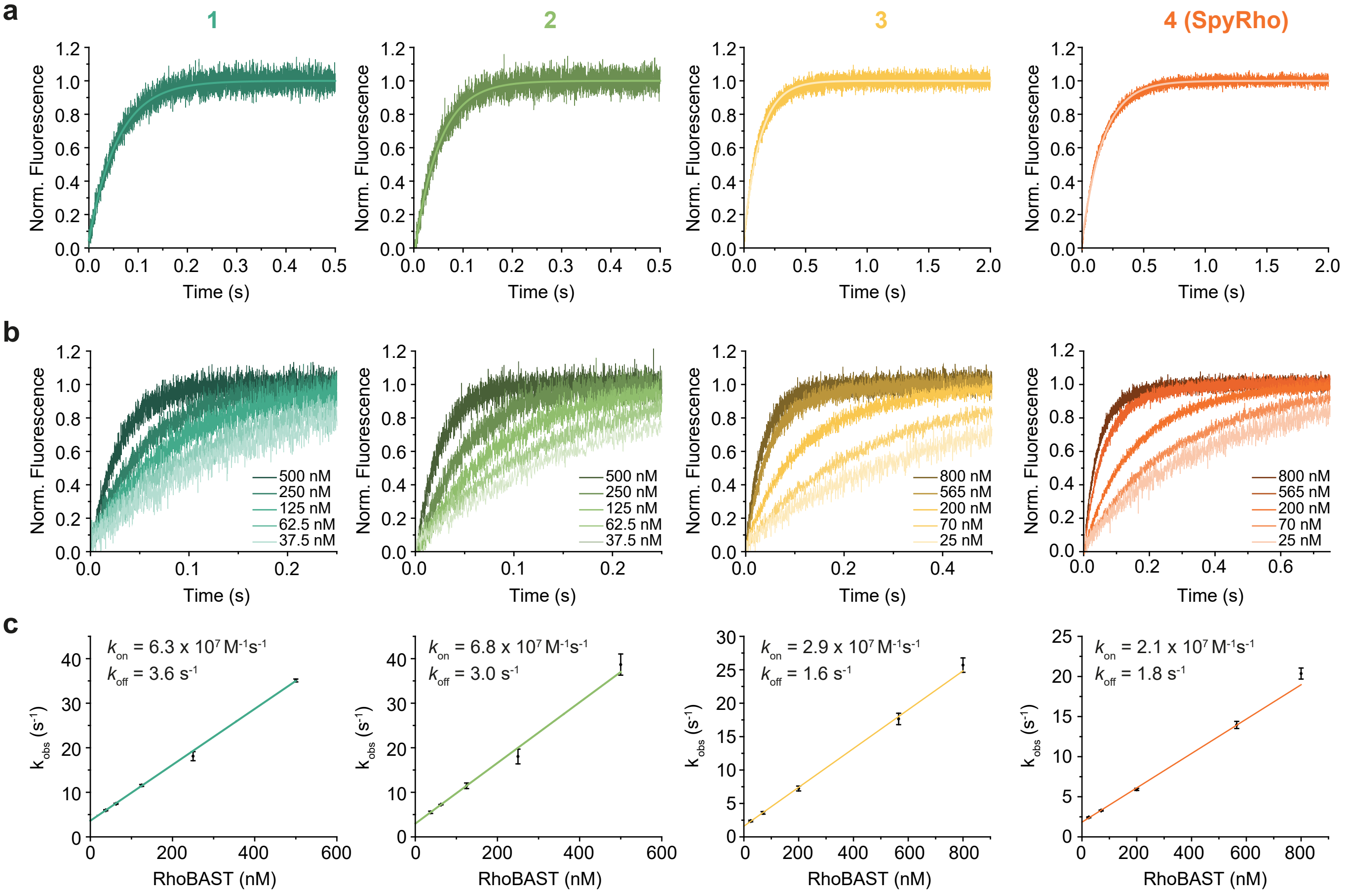

Supplementary Figure 2: Stopped-flow binding kinetics of RhoBAST:dye complexes. a) Normalized fluorescence increase over time upon mixing of dye (5 nM) and RhoBAST (for **1** or **2**: 250 nM RhoBAST; for **3** or **4 (SpyRho)** 200 nM RhoBAST) and single exponential fitting. **b)** Normalized fluorescence increase due to complex formation over time upon mixing of 5 nM dye with different RhoBAST concentrations at 25 °C. **c)** Observed kinetic rates (k_{obs}) were obtained by single-exponential fitting of the data shown in b). Linear fitting of the observed rates versus the RhoBAST concentration yields the dissociation (k_{off}) and association (k_{on}) rate coefficient. Data points represent mean ± s.d. of three independent measurements.

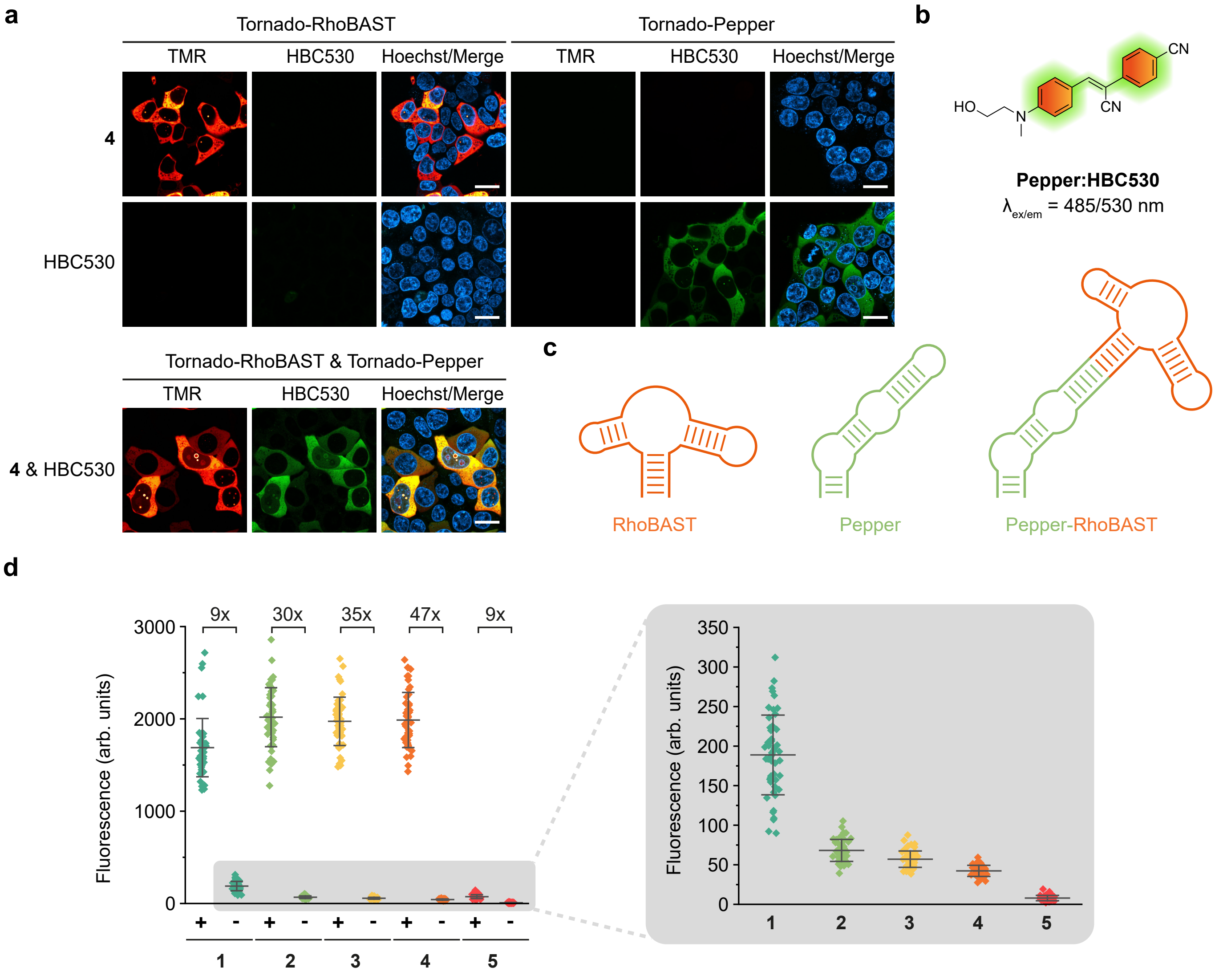

Supplementary Figure 3. Comparison of amide-substituted TMR derivatives in live cells. a) Orthogonality of RhoBAST and Pepper FLAP system. Confocal imaging of live HEK293T cells expressing circular RhoBAST and/or circular Pepper aptamer incubated with **4** (100 nM) and/or HBC530 (1 µM) for 30 min. At least three independent experiments were carried out with similar results. **b)** Chemical structure of HBC530. **c)** Schematic drawing of RhoBAST, Pepper and the designed Pepper-RhoBAST tandem aptamer construct. **d)** Left: Figure 2e reproduced; right: background fluorescence in negative control cells (-) shown with an expanded scale. Scale bars, 20 µm.

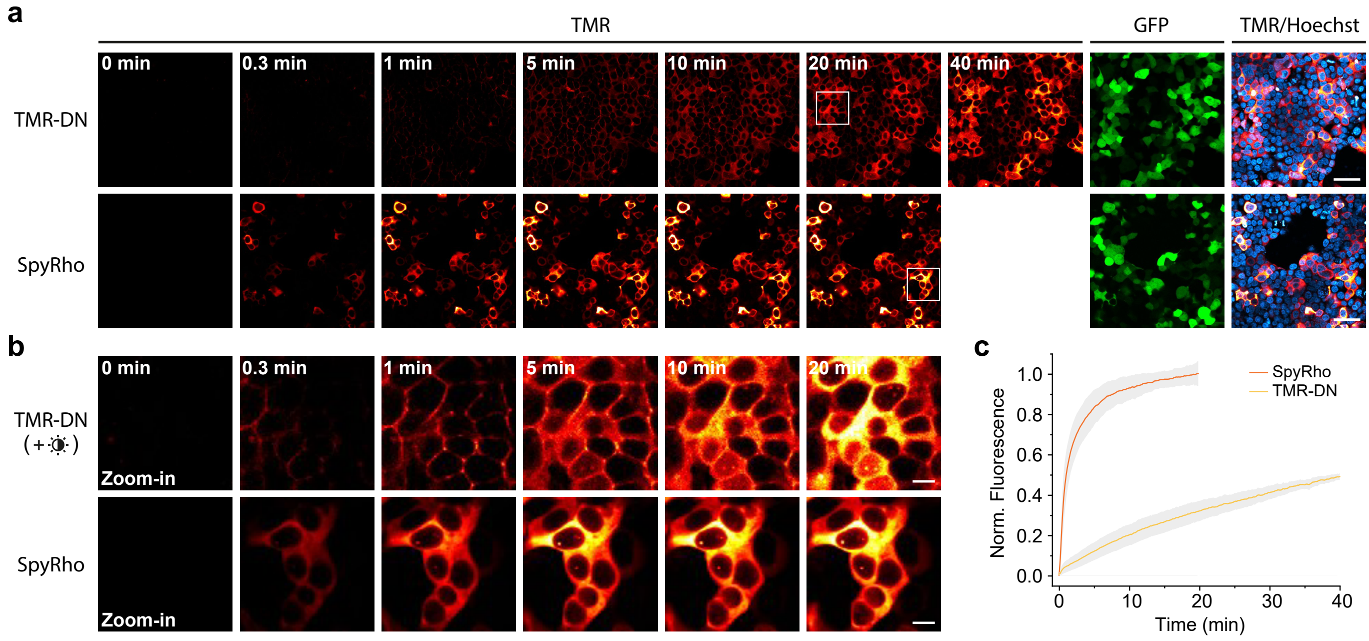

Supplementary Figure 4. Cellular uptake of SpyRho and TMR-DN. a) Confocal imaging of live HEK293T cells expressing circular RhoBAST and mEGFP (as transfection control) at different time points after addition of the dyes (100 nM). Scale bars, 50 µm. **b)** Zoom-ins of the highlighted regions of a). Scale bars, 10 µm. **c)** Quantification of the fluorescence increase over time (mean ± s.d.) in the cytosol of individual transfected cells ($N \geq 100$) shown in a) The fluorescence was normalized to the average fluorescence intensity of SpyRho at t = 20 min.

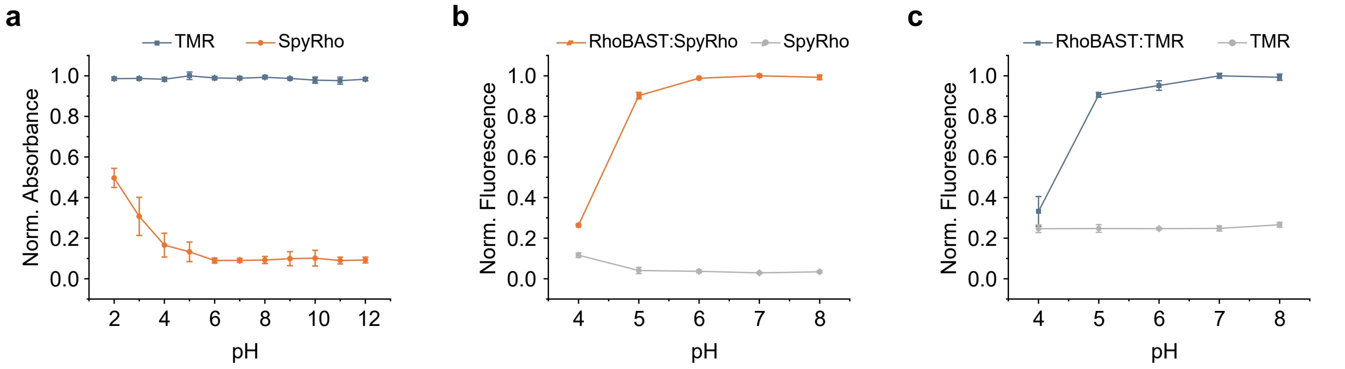

Supplementary Figure 5. pH dependency of the spirocyclization of SpyRho. a) Absorbance of TMR and SpyRho (both 5 µM) in 0.1 M phosphate buffer as a function of pH. Data points represent mean ± s.d. of three independent measurements and were normalized to the peak TMR absorbance. **b, c)** Fluorescence of SpyRho (panel b) and TMR (50 nM) (panel c) in the presence and absence of RhoBAST (1 µM) as a function of pH. Data points represent mean ± s.d. of three independent measurements and were recorded in modified ASB (20 mM phosphate buffer, 1 mM $MgCl_2$, 125 mM KCl) and normalized to the maximum fluorescence of the corresponding RhoBAST:dye complex.

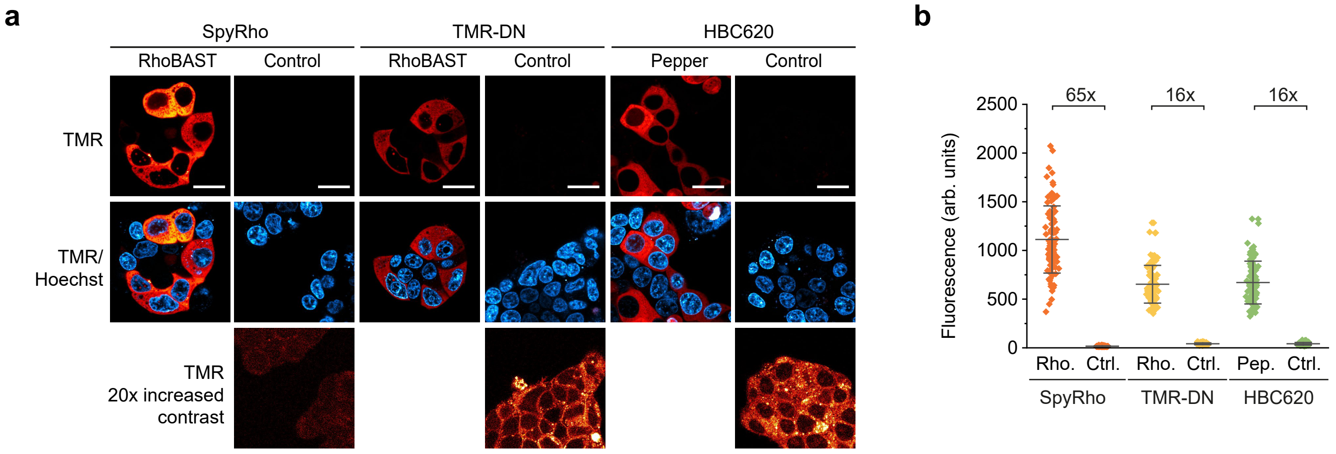

Supplementary Figure 6. Confocal imaging of live HEK293T cells expressing circular RhoBAST or circular Pepper aptamers, incubated with the corresponding fluorogenic probes (100 nM for SpyRho and TMR-DN, 1 µM HBC620) for 1 h. As a control, cells expressing the orthogonal aptamer (Pepper for SpyRho and TMR-DN, RhoBAST for HBC620) were used. **b)** Quantification of normalized TMR fluorescence (mean ± s.d.) in the cytosol of individual transfected cells (*N* = 100) of images in a). Scale bars, 20 µm.

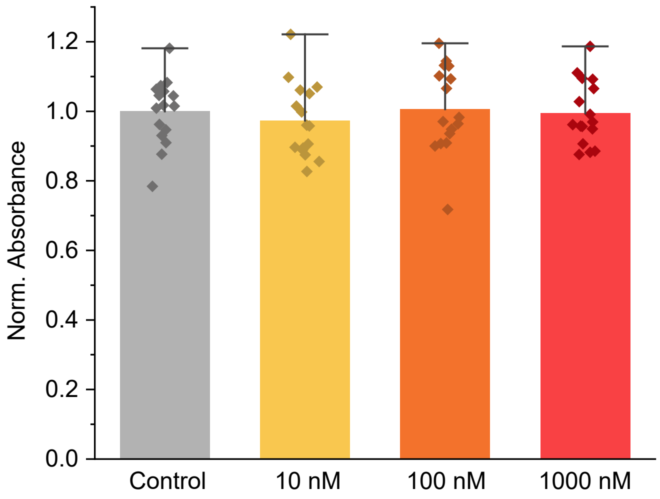

Supplementary Figure 7. Cell viability in the presence of SpyRho. Cell viability of HEK293T cells after 24-h incubation in the absence (control) and presence of SpyRho at various concentrations (10 nM, 100 nM and 1000 nM) using the Cell Titer96 AQ_{ueous} One Solution Cell Proliferation Assay (Promega). Measured absorbances at 450 nm (mean ± s.d. of 16 independent measurements) were normalized to the absorbances of the control cells.

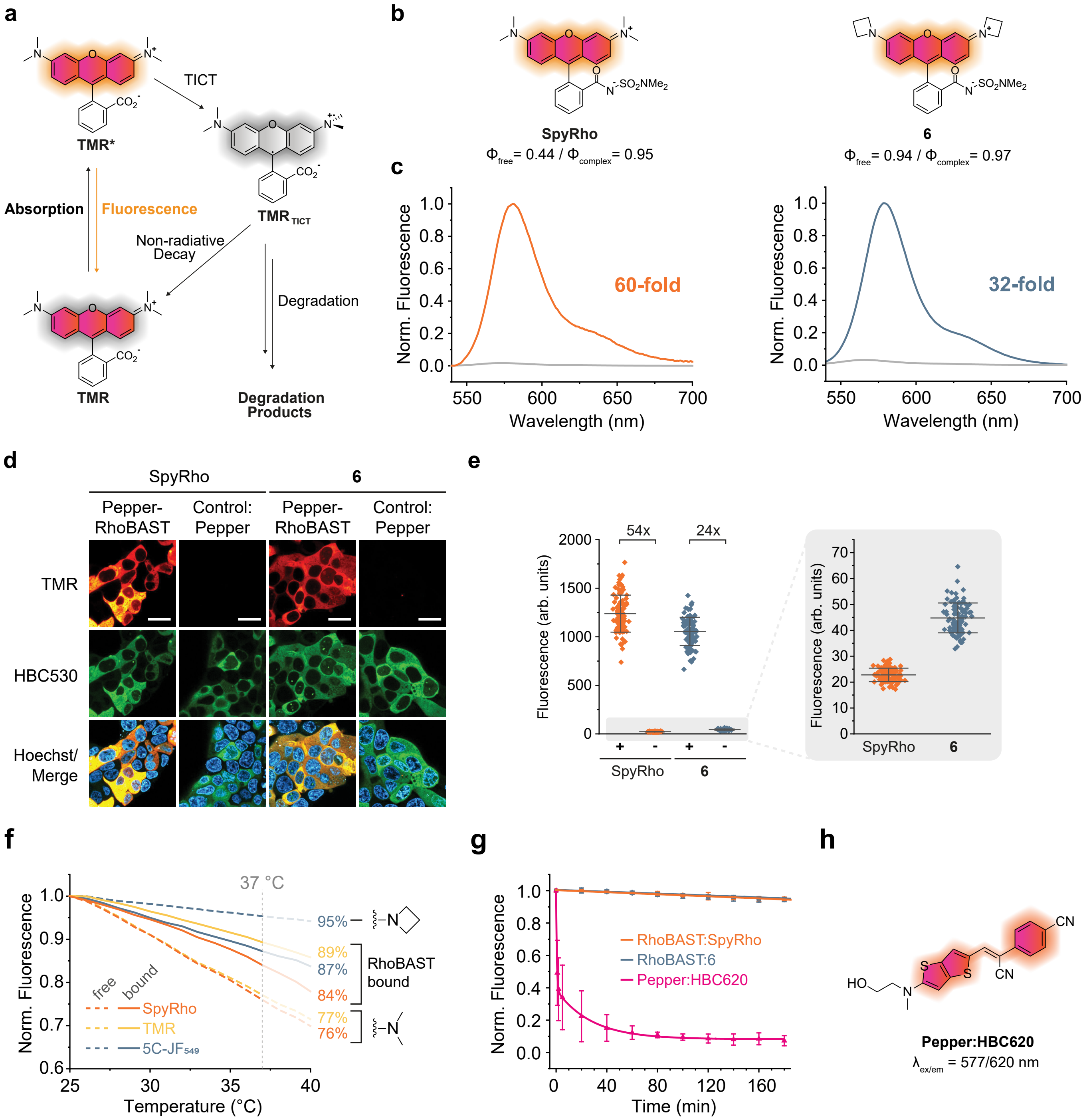

Supplementary Figure 8. Suppression of the twisted intramolecular charge transfer (TICT) mechanism. a) Schematic illustrating the TICT process. **b)** Chemical structure of SpyRho and the corresponding azetidinyl rhodamine **6**. **c)** Normalized emission spectra of SpyRho and **6** in the presence (colored line) and absence (grey line) of RhoBAST. Measurements were performed in ASB at 25 °C using 1 µM dye and 5 µM RhoBAST. **d)** Confocal imaging of live HEK293T cells expressing circular Pepper-RhoBAST or circular Pepper aptamer incubated with SpyRho or **6** (100 nM) and HBC530 (1 µM) for 1 h. **e)** Left: Quantification of normalized TMR fluorescence (mean ± s.d.) in the cytosol of individual transfected (Pepper-RhoBAST (+), Pepper (-)) cells (*N* = 50) from images as those shown in d); right: background fluorescence in negative control cells (-), shown with an expanded scale. **f)** Temperature dependence of the fluorescence of tetramethyl and azetidinyl rhodamines (500 nM) in the absence and presence of RhoBAST (2 µM) in ASB supplemented with 0.05% Tween 20. **g)** Fluorescence decrease (mean ± s.d. of three independent measurements) of aptamer:dye complexes (20 nM dye, 500 nM aptamer) under constant irradiation with an LED (λ_{max} = 567 nm, 680 µW mm^{-2}) in ASB supplemented with 0.05% Tween 20 at 25 °C. **h)** Chemical structure of HBC620. Scale bars, 20 µm.

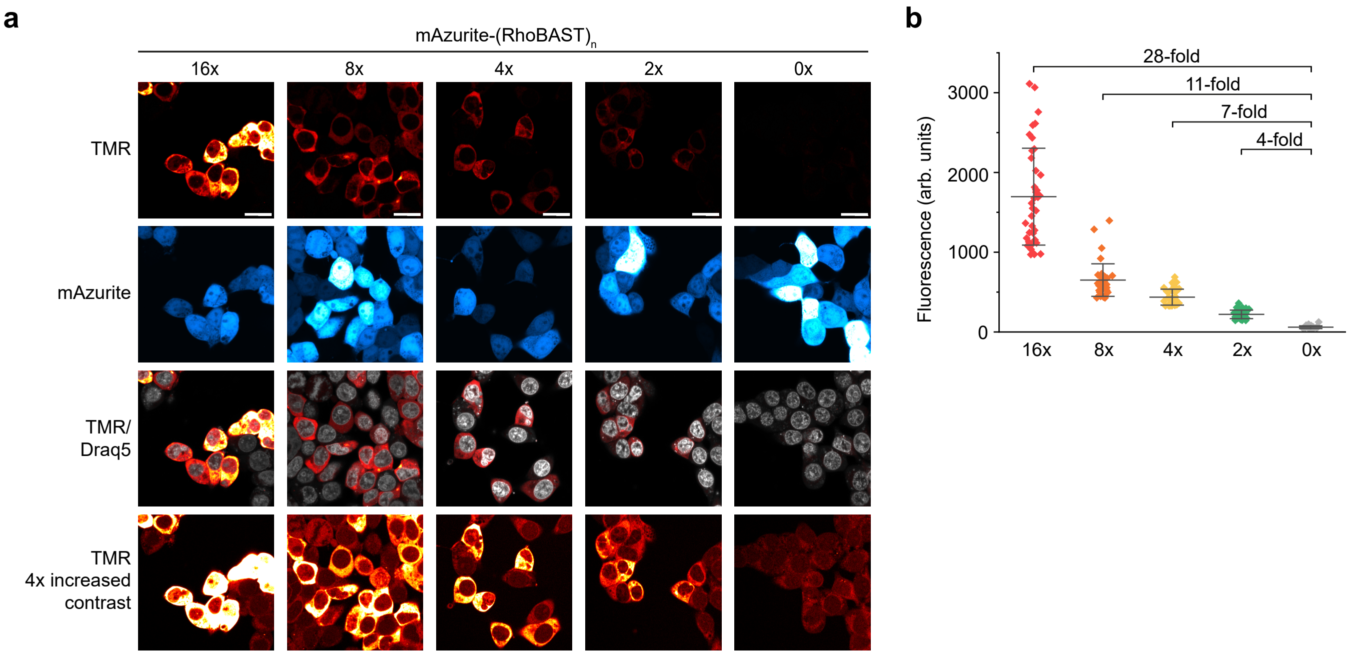

Supplementary Figure 9: CLSM imaging of mRNA using synonymous repeats of RhoBAST. a) Confocal images of living HEK293T expressing *mAzurite, mAzurite-RhoBAST*$_2$, *mAzurite-RhoBAST*$_4$, *mAzurite-RhoBAST*$_8$, *mAzurite-RhoBAST*$_{16}$ mRNAs using 100 nM SpyRho. **b)** Quantification of TMR fluorescence (mean ± s.d.) in the cytosol of individual transfected cells (N = 40) expressing *mAzurite-RhoBAST*$_n$ or *mAzurite* mRNAs from images as those shown in a). Scale bars, 20 µm.

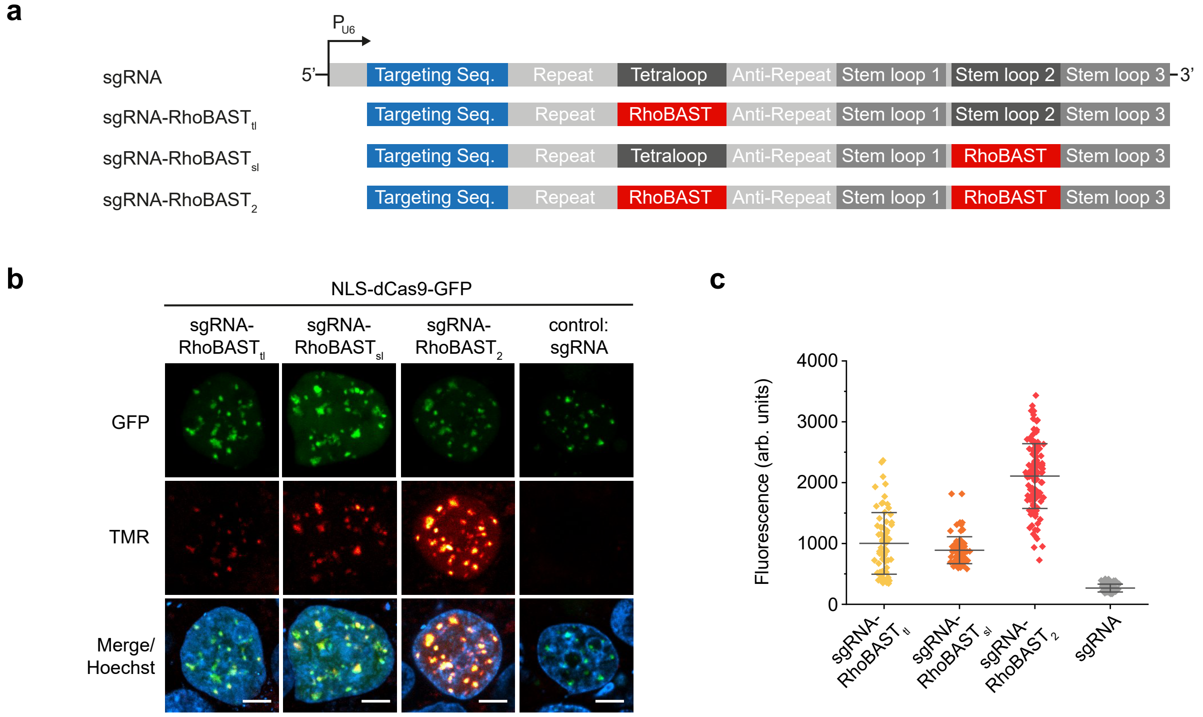

Supplementary Figure 10. Visualization of genomic loci using RhoBAST-modified sgRNAs. a) Schematic illustration of gene constructs for expression of unmodified and RhoBAST-modified sgRNA. **b)** Confocal imaging of HEK293T cells co-expressing NLS-dCas9-GFP and different sgRNAs constructs (plasmid ratio 1:10) targeting centromeres (targeting sequence: GAATCTGCAAGTGGATATT). Cells were incubated with SpyRho (100 nM) for 1 h prior to imaging. Maximum intensity projections of acquired z-stacks (500 nm step-size, 5 µm total) are shown for the GFP and TMR channels. **c)** Quantification of TMR fluorescence (mean ± s.d.) of nuclear foci (left to right N = 70, 91, 127 and 82) in individual transfected cells (left to right N = 10, 12, 18 and 12) of images in (c). Scale bars, 5 µm.

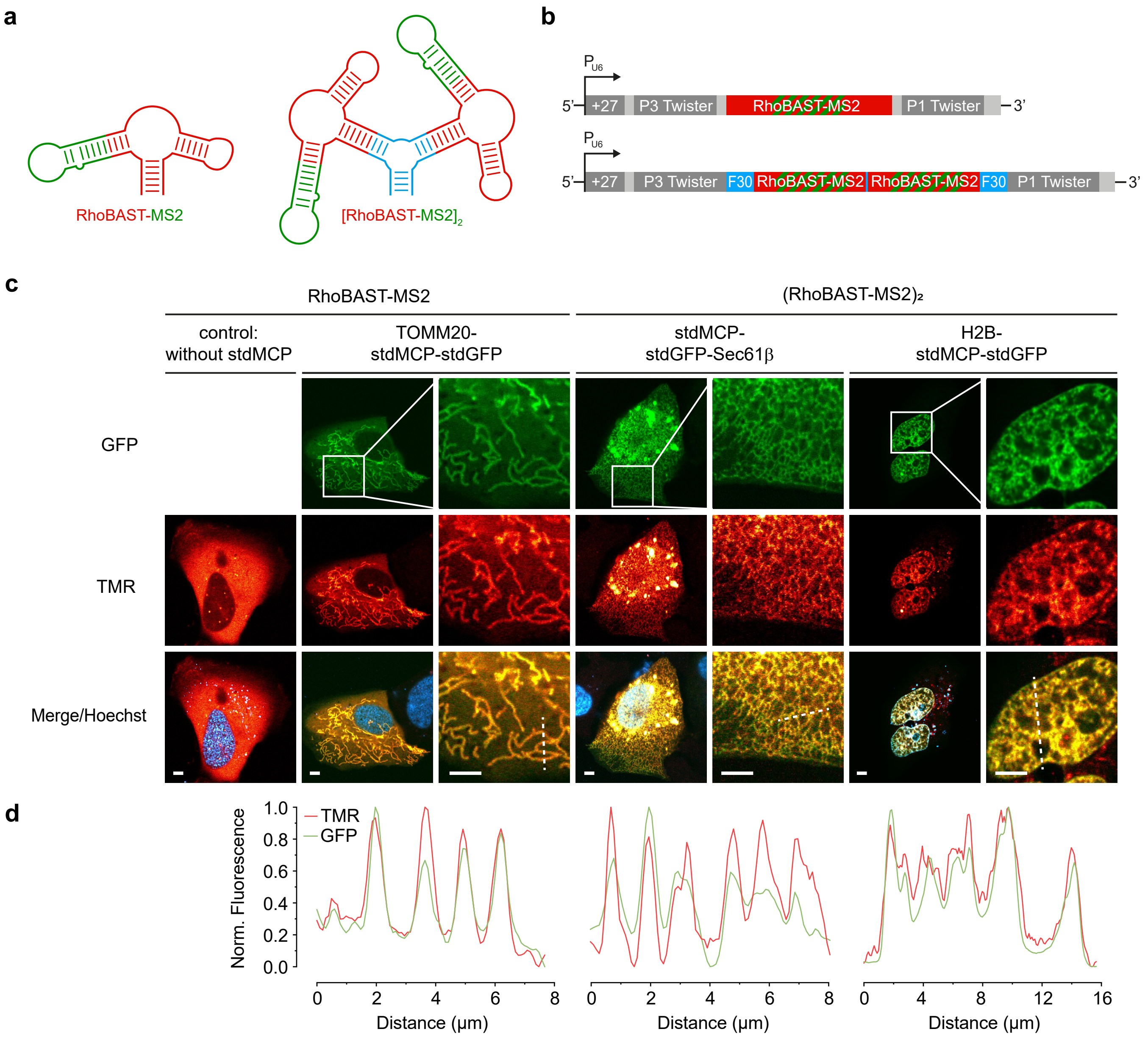

Supplementary Figure 11. Visualization of proteins using RhoBAST-MS2 tandem constructs. a) Schematic drawing of RhoBAST-MS2 and [RhoBAST-MS2]$_2$. **b)** Schematics of gene constructs for expressing circular *RhoBAST-MS2* and *[RhoBAST-MS2]$_2$* imager RNA. **c)** Confocal imaging of live U2OS cells co-expressing circular *RhoBAST-MS2* or *[RhoBAST-MS2]$_2$* and different stdMCP fusion proteins (plasmid ratio 10:1). Cells were incubated with SpyRho (100 nM) for 1 h prior to imaging. For clarity, the merged zoom-ins are shown without Hoechst. At least three independent experiments were carried out with similar results. **d)** Normalized fluorescence profiles of the indicated dashed lines in c). Scale bars, 5 µm.

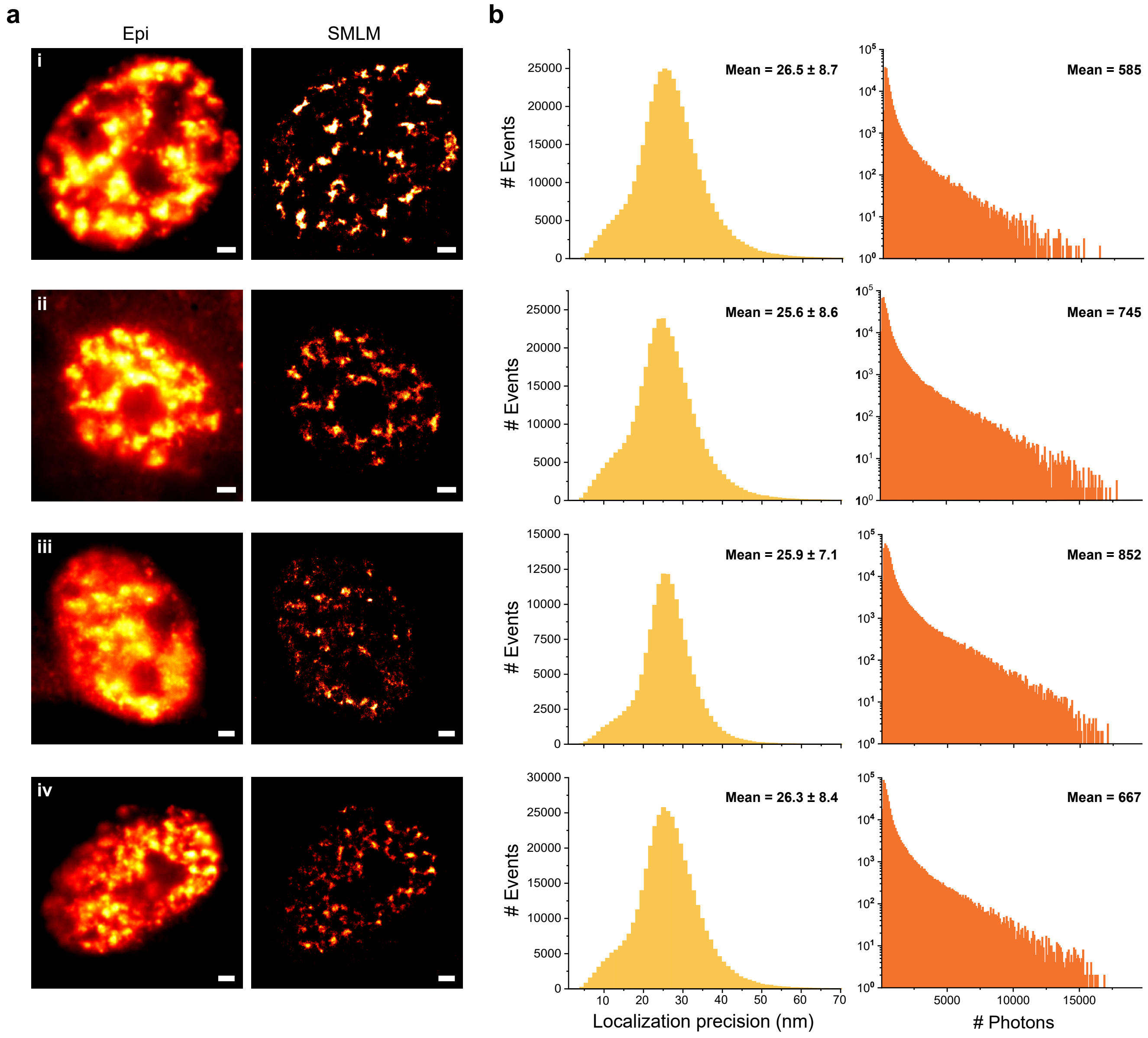

Supplementary Figure 12. SMLM imaging of CGG repeat-containing FMR1 using RhoBAST:SpyRho. a) Epifluorescence (Epi) and SMLM images of four nuclei (i-iv) of fixed Cos7 cells expressing *CGG₉₉-FMR1-GFP-RhoBAST₁₆* mRNA. Cells were incubated with SpyRho (1 nM) for 30 min prior to imaging. At least three independent experiments were carried out with similar results. **b)** Distributions of localization precision (left) and photon number (right) of the corresponding SMLM images shown in panel a. SMLM images were reconstructed from 80,000 frames, each with 30 ms exposure. The number of events recorded per unit area and time is 0.419, 0.686, 0.198 and 0.477 μm^{-2} s^{-1} for nucleus i-iv, respectively. Note that nucleus i is also shown in Figure 6 of the main manuscript. Scale bars, 2 µm.

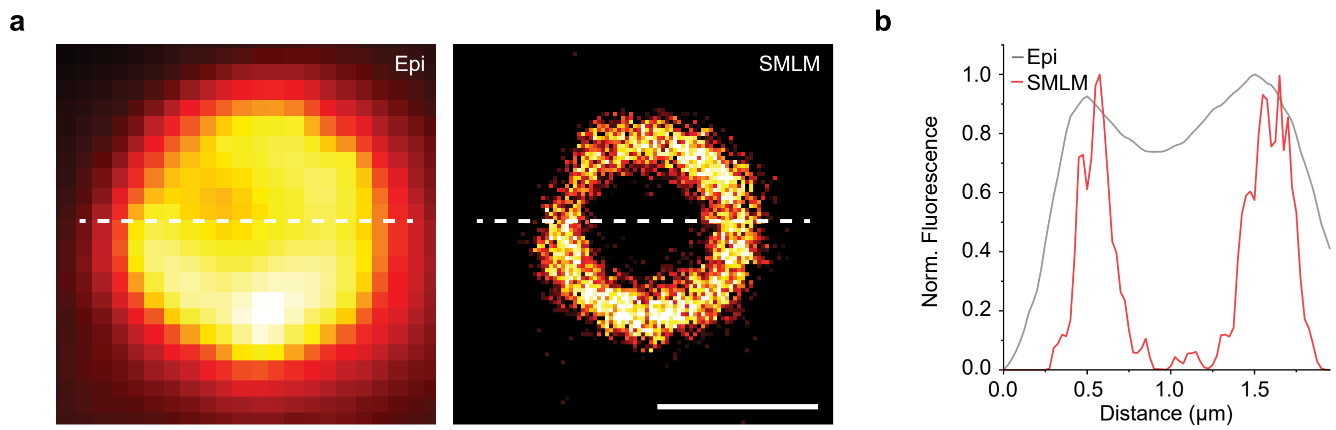

Supplementary Figure 13. SMLM imaging of circular RhoBAST. a) Epifluorescence (Epi) and SMLM images of the nucleus of a fixed HeLa cell expressing circular RhoBAST incubated with SpyRho (1 nM) for 30 min. The SMLM image with a mean localization precision of 16 nm was reconstructed using 40,000 frames (30 ms exposure time). At least three independent experiments were carried out with similar results. **b)** Normalized intensity profile along the dashed line in a). Scale bar, 1 µm.

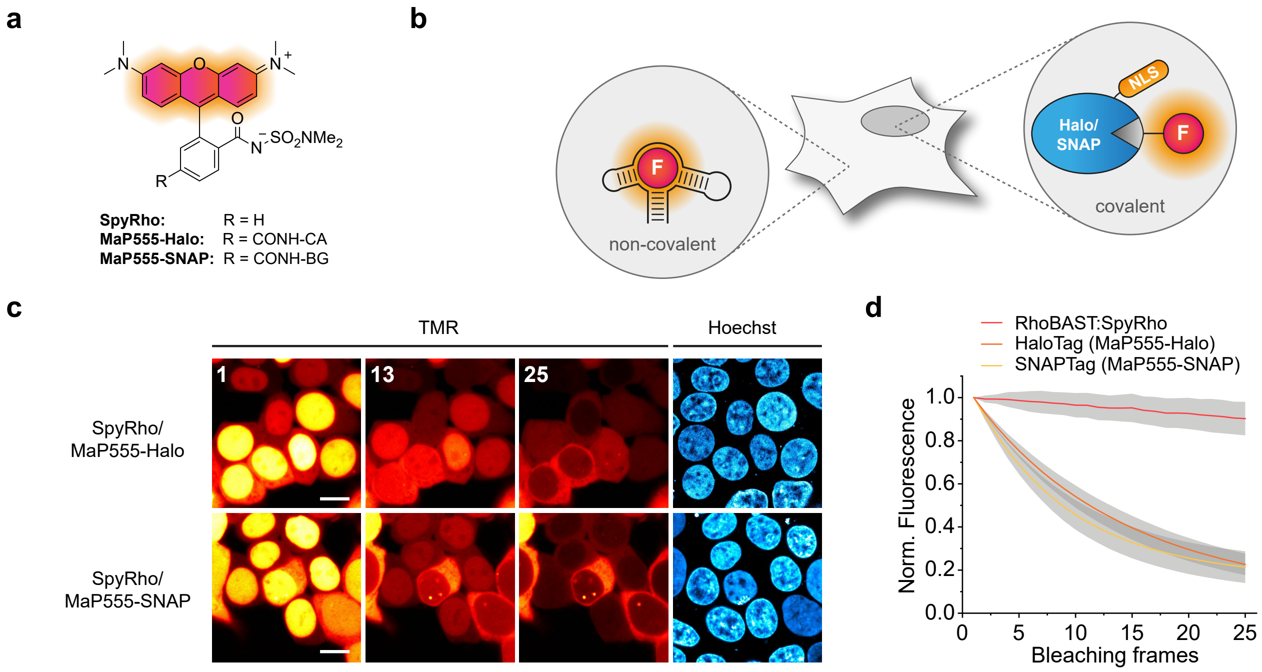

Supplementary Figure 14. Comparison of photostability of RhoBAST:SpyRho with MaP dyes. a) Chemical structure of SpyRho and employed MaP dyes (CA = chloroalkane; BG = benzyl-guanine). **b)** Illustration of localization of fluorescence arising from circular RhoBAST (cytosol) and HaloTag-SNAP-Tag-NLS fusion protein (nucleus) after labeling with the corresponding dyes (SpyRho, MaP555-Halo or MaP555-SNAP). **c)** Confocal imaging of HEK293T cells expressing circular RhoBAST and HaloTag-SNAP-Tag-NLS fusion protein (plasmid ratio 10:1). Cells were first incubated with the corresponding fluorogenic MaP555 derivative (MaP555-Halo: 50 nM, MaP555-SNAP: 500 nM) for 30 min;excess dye was removed by washing. Next, cells were incubated with SpyRho (100 nM) for 15 min and cells were imaged for 4 min (0.1 frames/s, 2× line accumulation, 530 µW). Numbers in the images correspond to the position in the sequence of acquired frames. **d)** Quantification of TMR fluorescence normalized to the initial value (mean ± s.d.) in the cytosol and nucleus of individual transfected cells (*N* = 30) of images as those shown in c). Scale bars, 10 µm.

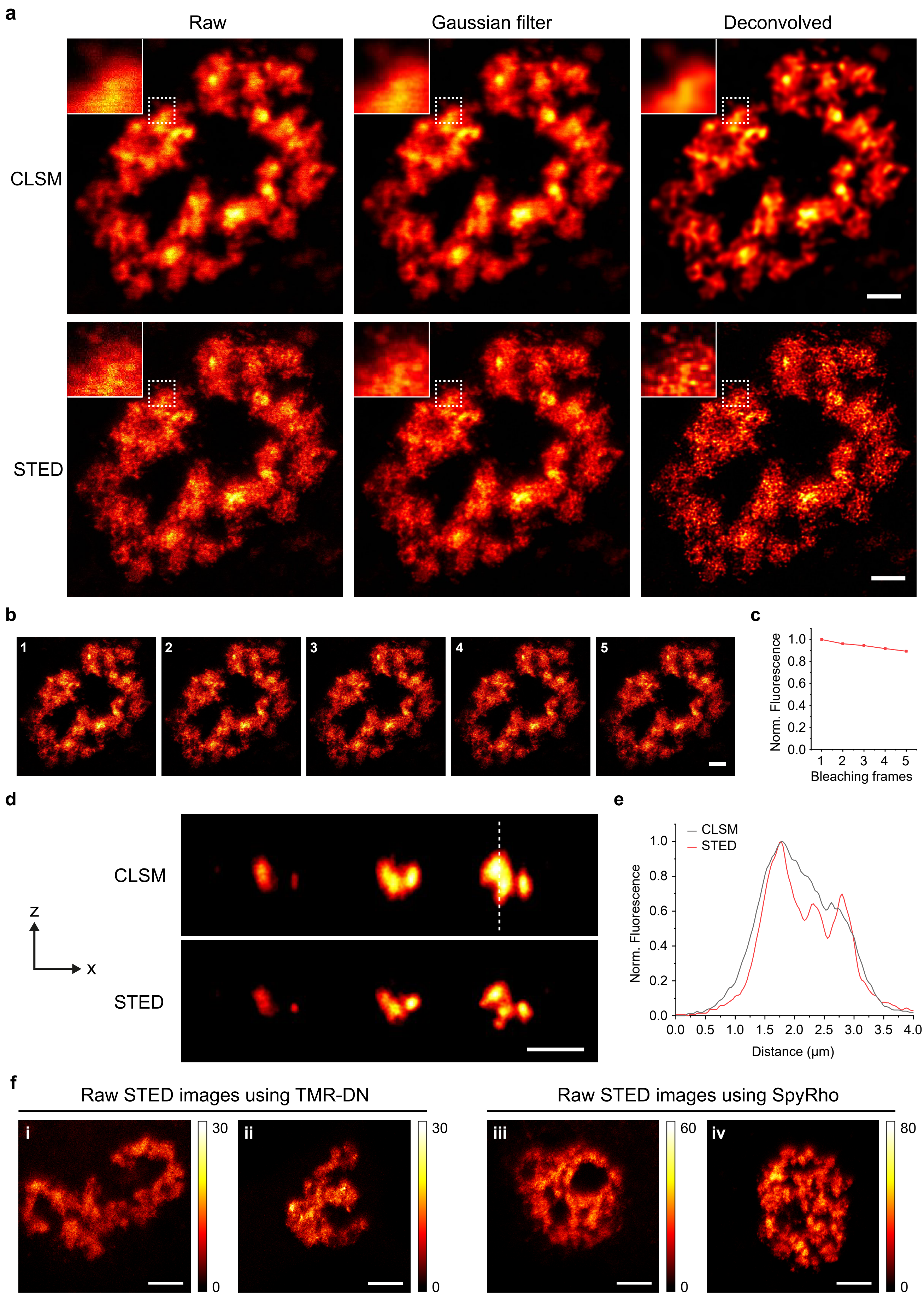

Supplementary Figure 15. Stimulated emission depletion (STED) RNA imaging using RhoBAST:SpyRho. a) Raw, Gaussian-filtered and deconvolved confocal laser scanning microscopy (CLSM) and 2D-STED images of a nucleus of a live Cos7

cell expressing *CGG$_{99}$-FMR1-GFP-RhoBAST$_{16}$* mRNA incubated with SpyRho (100 nM) for 1 h. Magnification of indicated region (dashed box) is shown as inset. **b)** Time-lapse STED imaging of a live Cos7 cell expressing *CGG$_{99}$-FMR1-GFP-RhoBAST$_{16}$* mRNA incubated with SpyRho (100 nM) for 1 h. Each frame was acquired using 16× line accumulation. Frames were acquired continuously. **c)** Quantification of the normalized TMR fluorescence of the images shown in **b**). **d)** XZ scans using 3D-STED mode of the nucleus of a live Cos7 cell expressing *CGG$_{99}$-FMR1-GFP-RhoBAST$_{16}$* mRNA incubated with SpyRho (100 nM) for 1 h. **e)** Normalized fluorescence profile along the dashed line shown in d). Scale bars, 2 µm. **f)** Raw 2D-STED images of nuclei of live Cos7 cells expressing *CGG$_{99}$-FMR1-GFP-RhoBAST$_{16}$* mRNA incubated with either SpyRho or TMR-DN (100 nM) for 1 h. Scale bars, 5 µm.

Supplementary Tables

Supplementary Table 1. Properties of fluorogenic rhodamines and the corresponding RhoBAST:dye complexes. The excitation wavelength (λ_{ex}), emission wavelength (λ_{em}), peak extinction coefficient (ε), fluorescence quantum yield (Φ_F), and fluorescence turn-on (1 µM probe, 5 µM RhoBAST) were measured in ASB at 25 °C. Absolute quantum yields were measured for rhodamines; values for RhoBAST:dye complexes were determined relative to sulforhodamine 101. The brightness was calculated as the product of ε and Φ_F. Equilibrium dissociation constants (K_D) were measured in ASB supplemented with 0.05% Tween 20 at 25°C using 10 nM probe.

	Free dye						RhoBAST:dye complex							
	λ_{ex} [nm]	λ_{em} [nm]	ε [$M^{-1}cm^{-1}$]	Φ_F	Brightness [$M^{-1}cm^{-1}$]	D_{50}	λ_{ex} [nm]	λ_{em} [nm]	ε [$M^{-1}cm^{-1}$]	Φ_F	Brightness [$M^{-1}cm^{-1}$]	Abs. increase [n-fold]	Turn-on [n-fold]	K_D [nM]
**TMR**	549	573	75000 ± 3000	0.48	36000	12	560	581	80000 ± 3000	0.92 ± 0.02	74000	1.1 ± 0.1	2.9 ± 0.2	12 ± 1
**1**	551	578	23000 ± 2000	0.46	11000	43	560	581	60000 ± 1000	0.98 ± 0.03	59000	2.6 ± 0.3	18 ± 4	43 ± 4
**2**	552	575	24000 ± 1000	0.47	11000	55	561	582	69000 ± 1000	0.95 ± 0.02	66000	2.9 ± 0.2	15 ± 1	28 ± 1
**3**	551	575	12000 ± 2000	0.45	5400	70	561	581	69000 ± 1000	0.97 ± 0.03	67000	5.7 ± 0.9	52 ± 19	27 ± 2
**4 (SpyRho)**	551	573	5300 ± 600	0.44	2300	70	562	581	65000 ± 1000	0.95 ± 0.03	62000	12 ± 2	60 ± 18	34 ± 2
**5**	549	571	<200	-	-	>70	565	584	ND[a]	0.98 ± 0.02	ND[a]	>100	>900	>10000[b]
**6**	552	573	6700 ± 100	0.94	6300	>70	562	580	74000 ± 3000	0.97 ± 0.03	72000	11 ± 1	32 ± 7	100 ± 8

[a] Not determined (ND) due to incomplete complexation.
[b] Dissociation constant was determined using 50 nM probe.

Supplementary Table 2. Kinetic parameters. Association (k_{on}) and dissociation (k_{off}) rate coefficients of the RhoBAST:dye complexes were obtained from stopped-flow measurements in ASB supplemented with 0.05% Tween 20 at 25 °C.

	k_{on} [$M^{-1}s^{-1}$]	k_{off} [s^{-1}]
RhoBAST:**1**	$6.3 \pm 0.1 \times 10^7$	3.6 ± 0.1
RhoBAST:**2**	$6.8 \pm 0.3 \times 10^7$	3.0 ± 0.2
RhoBAST:**3**	$2.9 \pm 0.1 \times 10^7$	1.6 ± 0.1
RhoBAST:SpyRho (**4**)	$2.1 \pm 0.1 \times 10^7$	1.8 ± 0.1
RhoBAST:**6**	$1.2 \pm 0.1 \times 10^7$	2.6 ± 0.1

Supplementary Table 3. DNA Sequences.

pAV-U6+27-tornado-RhoBAST	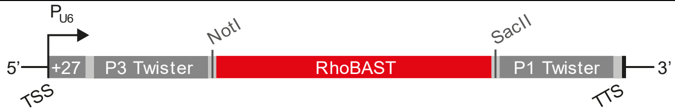
	GTCGACGGGCCGCACTCGCCGGTCCCAAGCCCGGATAAAATGGAGGGGGCGGG<br>AAACCGCCTAACCATGCCGAGT***GCGGCCGC*****ACCTCCGCGAAAGCGGTGAAGGAG**<br>**AGGCGCAAGGTTAACCGCCTCAGGT**GTGG***CCGCGG***TCGGCGTGGACTGTAGAAC<br>ACTGCCAATGCCGGTCCCAAGCCCGGATAAAAGTGGAGGGTACAGTCCACGCTC<br>TAGA
pAV-U6+27-tornado-Pepper	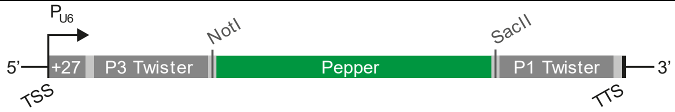
	GTCGACGGGCCGCACTCGCCGGTCCCAAGCCCGGATAAAATGGAGGGGGCGGG<br>AAACCGCCTAACCATGCCGAGT***GCGGCCGC*****CCCAATCGTGGCGTGTCGGCCTGC**<br>**TTCGGCAGGCACTGGCGCCGG**GTGG***CCGCGG***TCGGCGTGGACTGTAGAACACTG<br>CCAATGCCGGTCCCAAGCCCGGATAAAAGTGGAGGGTACAGTCCACGCTCTAGA
pAV-U6+27-tornado-Pepper-RhoBAST	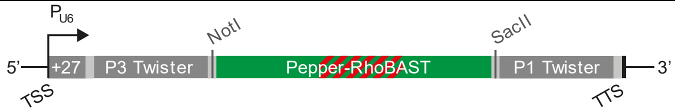
	GTCGACGGGCCGCACTCGCCGGTCCCAAGCCCGGATAAAATGGAGGGGGCGGG<br>AAACCGCCTAACCATGCCGAGT***GCGGCCGC*****CCCAATCGTGGCGTGTCGGCCTGC**<br>**AACCTCCGCGAAAGCGGTGAAGGAGAGGCGCAAGGTTAACCGCCTCAGGTTGCA**<br>**GGCACTGGCGCCGG**GTGG**CCGCGG**TCGGCGTGGACTGTAGAACACTGCCAATGC<br>CGGTCCCAAGCCCGGATAAAAGTGGAGGGTACAGTCCACGCTCTAGA
pAV-U6+27-tornado-RhoBAST-MS2	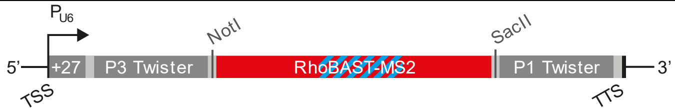
	GTCGACGGGCCGCACTCGCCGGTCCCAAGCCCGGATAAAATGGAGGGGGCGGG<br>AAACCGCCTAACCATGCCGAGT***GCGGCCGC*****ACCTCGGCGGCCAACATGAGGATC**<br>**ACCCATGTCTGCAGGGCCGCCGTGAAGGAGAGGCGCAAGGTTAACCGCCTCAGG**<br>**T**GTGG***CCGCGG***TCGGCGTGGACTGTAGAACACTGCCAATGCCGGTCCCAAGCCC<br>GGATAAAAGTGGAGGGTACAGTCCACGCTCTAGA
pAV-U6+27-tornado-[RhoBAST-MS2]2	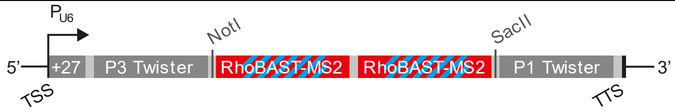
	GTCGACGGGCCGCACTCGCCGGTCCCAAGCCCGGATAAAATGGAGGGGGCGGG<br>AAACCGCCTAACCATGCCGAGT***GCGGCCGC***TTGCCATGTGTATCGG**AAGGCCTC**<br>**CGCGCCCAGGTGGAGGATCACCCCACCCTGCAGGGGCGCGGTGAAGGAGCGGCA**<br>**CAAGGTTAACTGCCGCAGGCCTT**CCGATACTCTGATGATCC**GGAACCTCCGGGG**<br>**CCAACATGAGGATCACCCATGTCTGCAGGGCCCCGGTGAAGGAGAGGCGCAAGG**

	**TTAACCGCCTCAGGTTCC**GGATCATTCATGGCAAGTGG***CCGCGG***TCGGCGTGGA<br>CTGTAGAACACTGCCAATGCCGGTCCCAAGCCCGGATAAAAGTGGAGGGTACAG<br>TCCACGCTCTAGA
pcDNA3-HaloTag7-Sam68	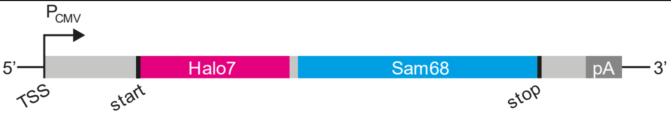
	AAGCTTCGCCGCCACC**ATGGGATCCGAAATCGGTACTGGCTTTCCATTCGACCC<br>CCATTATGTGGAAGTCCTGGGCGAGCGCATGCACTACGTCGATGTTGGTCCGCG<br>CGATGGCACCCCTGTGCTGTTCCTGCACGGTAACCCGACCTCCTCCTACGTGTG<br>GCGCAACATCATCCCGCATGTTGCACCGACCCATCGCTGCATTGCTCCAGACCT<br>GATCGGTATGGGCAAATCCGACAAACCAGACCTGGGTTATTTCTTCGACGACCA<br>CGTCCGCTTCATGGATGCCTTCATCGAAGCCCTGGGTCTGGAAGAGGTCGTCCT<br>GGTCATTCACGACTGGGGCTCCGCTCTGGGTTTCCACTGGGCCAAGCGCAATCC<br>AGAGCGCGTCAAAGGTATTGCATTTATGGAGTTCATCCGCCCTATCCCGACCTG<br>GGACGAATGGCCAGAATTTGCCCGCGAGACCTTCCAGGCCTTCCGCACCACCGA<br>CGTCGGCCGCAAGCTGATCATCGATCAGAACGTTTTTATCGAGGGTACGCTGCC<br>GATGGGTGTCGTCCGCCCGCTGACTGAAGTCGAGATGGACCATTACCGCGAGCC<br>GTTCCTGAATCCTGTTGACCGCGAGCCACTGTGGCGCTTCCCAAACGAGCTGCC<br>AATCGCCGGTGAGCCAGCGAACATCGTCGCGCTGGTCGAAGAATACATGGACTG<br>GCTGCACCAGTCCCCTGTCCCGAAGCTGCTGTTCTGGGGCACCCCAGGCGTTCT<br>GATCCCACCGGCCGAAGCCGCTCGCCTGGCCAAAAGCCTGCCTAACTGCAAGGC<br>TGTGGACATCGGCCCGGGTCTGAATCTGCTGCAAGAAGACAACCCGGACCTGAT<br>CGGCAGCGAGATCGCGCGCTGGCTGTCTACTCTGGAGATTTCCGGT**GGTTCCGG<br>CTCAGGA**TCTAGAGGATCC [...] ATCCATATGGACGTTATTA**AAAACAAACAGGA<br>GGGA
pcDNA5-FRT/TO-NLS-dCas9-NLS-GFP	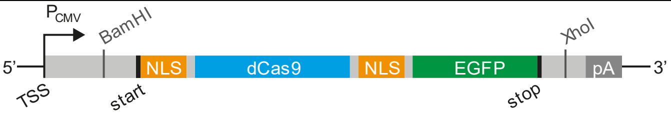
	CCGAGCTC***GGATCC***TCGCCACCATGGCTAGC**CCCAAAAAGAAGAGGAAAGTGGA<br>CAAGAAGTATTCT** [...] **GCTCGGAGGGGAT**GAGGGAGCT**CCCAAGAAAAAGCGCA<br>A**GGTAGGTAGTTCC**GTGAGCAAGGGCGAGGAGCTGTTCACCGGGGTGGTGCCCA<br>TCCTGGTCGAGCTGGACGGCGACGTAAACGGCCACAAGTTCAGCGTGTCCGGCG<br>AGGGCGAGGGCGATGCCACCTACGGCAAGCTGACCCTGAAGTTCATCTGCACCA<br>CCGGCAAGCTGCCCGTGCCCTGGCCCACCCTCGTGACCACCCTGACCTACGGCG<br>TGCAGTGCTTCAGCCGCTACCCCGACCACATGAAGCAGCACGACTTCTTCAAGT<br>CCGCCATGCCCGAAGGCTACGTCCAGGAGCGCACCATCTTCTTCAAGGACGACG<br>GCAACTACAAGACCCGCGCCGAGGTGAAGTTCGAGGGCGACACCCTGGTGAACC<br>GCATCGAGCTGAAGGGCATCGACTTCAAGGAGGACGGCAACATCCTGGGGCACA<br>AGCTGGAGTACAACTACAACAGCCACAACGTCTATATCATGGCCGACAAGCAGA<br>AGAACGGCATCAAGGTGAACTTCAAGATCCGCCACAACATCGAGGACGGCAGCG<br>TGCAGCTCGCCGACCACTACCAGCAGAACACCCCCATCGGCGACGGCCCCGTGC<br>TGCTGCCCGACAACCACTACCTGAGCACCCAGTCCGCCCTGAGCAAAGACCCCA<br>ACGAGAAGCGCGATCACATGGTCCTGCTGGAGTTCGTGACCGCCGCCGGGATCA<br>CTCTCGGCATGGACGAGCTGTACAAGTAA**G***CTCGAG***TCTAG
pAV-U6-centromer-sgRNA	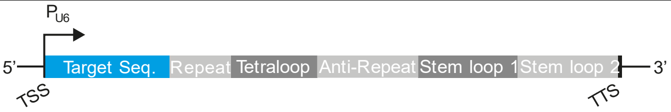
	GCC***GGATCC***AAGGTCGGGCAGGAAGAGGGCCTATTTCCCATGATTCCTTCATAT<br>TTGCATATACGATACAAGGCTGTTAGAGAGATAATTGGAATTAATTTGACTGTA<br>AACACAAAGATATTAGTACAAAATACGTGACGTAGAAAGTAATAATTTCTTGGG<br>TAGTTTGCAGTTTTAAAATTATGTTTTAAAATGGACTATCATATGCTTACCGTA<br>ACTTGAAAGTATTTCGATTTCTTGGCTTTATATATCTTGTGGAAAGGACGAAAC<br>ACC**GAATCTGCAAGTGGATATTGTTTGAGAGCTAGAAATAGCAAGTTCAAATAA<br>GGCTAGTCCGTTATCAACTTGAAAAAGTGGCACCGAGTCGGTGC**TTTTTTGTTT<br>TA***CTCGAG***CCGC

pAV-U6-centromer-sgRNA- RhoBAST$_{tl}$	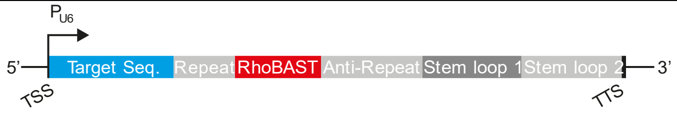
	GCC***GGATCC***AAGGTCGGGCAGGAAGAGGGCCTATTTCCCATGATTCCTTCATAT<br>TTGCATATACGATACAAGGCTGTTAGAGAGATAATTGGAATTAATTTGACTGTA<br>AACACAAAGATATTAGTACAAAATACGTGACGTAGAAAGTAATAATTTCTTGGG<br>TAGTTTGCAGTTTTAAAATTATGTTTTAAAATGGACTATCATATGCTTACCGTA<br>ACTTGAAAGTATTTCGATTTCTTGGCTTTATATATCTTGTGGAAAGGACGAAAC<br>ACCGAATCTGCAAGTGGATATTGTTTGAGAGCTAGGAACCTCCGCGAAAGCGGT<br>GAAGGAGAGGCGCAAGGTTAACCGCCTCAGGTTCCTAGCAAGTTCAAATAAGGC<br>TAGTCCGTTATCAACTTGAAAAAGTGGCACCGAGTCGGTGCTTTTTTGTTTTA***C***<br>***TCGAG***CCGC
pAV-U6-centromer-sgRNA-RhoBAST$_{sl}$	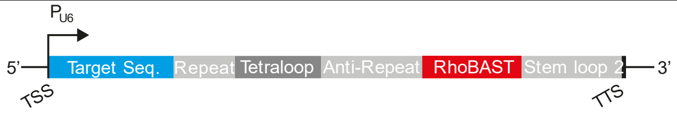
	GCC***GGATCC***AAGGTCGGGCAGGAAGAGGGCCTATTTCCCATGATTCCTTCATAT<br>TTGCATATACGATACAAGGCTGTTAGAGAGATAATTGGAATTAATTTGACTGTA<br>AACACAAAGATATTAGTACAAAATACGTGACGTAGAAAGTAATAATTTCTTGGG<br>TAGTTTGCAGTTTTAAAATTATGTTTTAAAATGGACTATCATATGCTTACCGTA<br>ACTTGAAAGTATTTCGATTTCTTGGCTTTATATATCTTGTGGAAAGGACGAAAC<br>ACCGAATCTGCAAGTGGATATTGTTTGAGAGCTAGAAATAGCAAGTTCAAATAA<br>GGCTAGTCCGTTATCAACTTGGAACCTCCGCGAAAGCGGTGAAGGAGAGGCGCA<br>AGGTTAACCGCCTCAGGTTCCAAGTGGCACCGAGTCGGTGCTTTTTTGTTTTA***C***<br>***TCGAG***CCGC
pAV-U6-centromer-sgRNA-RhoBAST$_2$	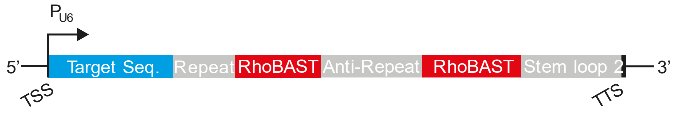
	GCC***GGATCC***AAGGTCGGGCAGGAAGAGGGCCTATTTCCCATGATTCCTTCATAT<br>TTGCATATACGATACAAGGCTGTTAGAGAGATAATTGGAATTAATTTGACTGTA<br>AACACAAAGATATTAGTACAAAATACGTGACGTAGAAAGTAATAATTTCTTGGG<br>TAGTTTGCAGTTTTAAAATTATGTTTTAAAATGGACTATCATATGCTTACCGTA<br>ACTTGAAAGTATTTCGATTTCTTGGCTTTATATATCTTGTGGAAAGGACGAAAC<br>ACCGAATCTGCAAGTGGATATTGTTTGAGAGCTAGGAACCTCCGCGAAAGCGGT<br>GAAGGAGAGGCGCAAGGTTAACCGCCTCAGGTTCCTAGCAAGTTCAAATAAGGC<br>TAGTCCGTTATCAACTTAAGGCCTCCGCGAAAGCGGTGAAGGAGCGGCACAAGG<br>TTAACTGCCGCAGGCCTTAAGTGGCACCGAGTCGGTGCTTTTTTGTTTTA***CTCG***<br>***AG***CCGC
pcDNA5-FRT-H2B-stdMCP-stdGFP	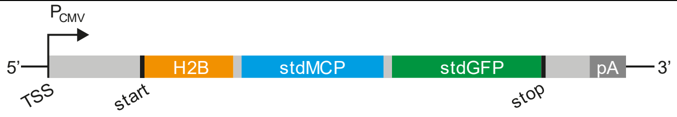
	AAGCTTGGCCACCATGCCAGAGCCAGCGAAGTCTGCTCCCGCCCCGAAAAAGGG<br>CTCCAAGAAGGCGGTGACTAAGGCGCAGAAGAAAGGCGGCAAGAAGCGCAAGCG<br>CAGCCGCAAGGAGAGCTATTCCATCTATGTGTACAAGGTTCTGAAGCAGGTCCA<br>CCCTGACACCGGCATTTCGTCCAAGGCCATGGGCATCATGAATTCGTTTGTGAA<br>CGACATTTTCGAGCGCATCGCAGGTGAGGCTTCCCGCCTGGCGCATTACAACAA<br>GCGCTCGACCATCACCTCCAGGGAGATCCAGACGGCCGTGCGCCTGCTGCTGCC<br>TGGGGAGTTGGCCAAGCACGCCGTGTCCGAGGGTACTAAGGCCATCACCAAGTA<br>CACCAGCGCTAAGCTGTTATTAATTAACGCTTCTAACT [...] ATGGATGAATTGT<br>ACAAATAATCTAGAG
pcDNA5-FRT-TOMM20-stdMCP-stdGFP	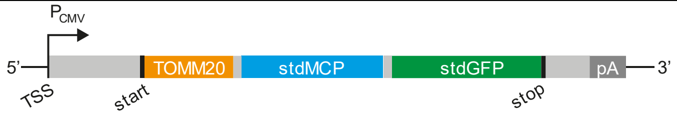
	AAGCTTGGCCACCATGGGTCGGAACAGCGCCATCGCCGCGGGCGTGTGCGGTGC<br>CCTCTTCATAGGGTACTGCATCTACTTTGACCGCAAAAGACGAAGTGACCCCAA<br>CTTCCTGTTATTAATTAACGCTTCTAACT [...] ATGGATGAATTGTACAAATAAT<br>CTAGAG

<table>
<tr><td rowspan="2">pcDNA5-FRT-stdMCP-stdGFP- Sec61β</td><td>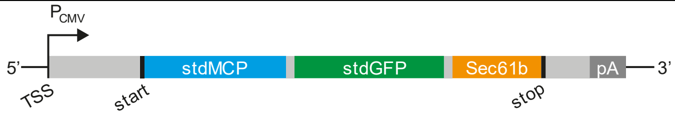
</td></tr>
<tr><td>AAGCTTGGCCACCATGGCTTCTAACT [...] ATGGATGAATTGTACAAATCCGGAC
TCAGATCTGGCTCCAGCGCAGGCAGCGCATCCGGCGGAAGCGGAAGCCCTGGTC
CGACCCCCAGTGGCACTAACGTGGGATCCTCAGGGCGCTCTCCCAGCAAAGCAG
TGGCCGCCCGGGCGGCGGGATCCACTGTCCGGCAGAGGAAAAATGCCAGCTGTG
GGACAAGGAGTGCAGGCCGCACAACCTCGGCAGGCACCGGGGGATGTGGCGAT
TCTACACAGAAGATTCACCTGGGCTCAAAGTTGGCCCTGTTCCAGTATTGGTTA
TGAGTCTTCTGTTCATCGCTTCTGTATTTATGTTGCACATTTGGGCAAGTACA
CTCGTTCGTAGTCTAGAG</td></tr>
<tr><td rowspan="2">mAzurite-C1-RhoBAST$_2$</td><td>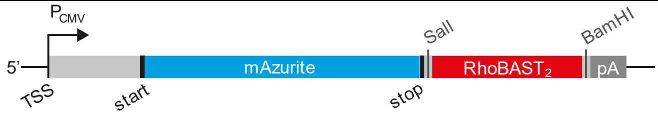
</td></tr>
<tr><td>GCTAGCGCTACCGGTCGCCACCATGGTGAGCAA [...] TACAAGTAAGGACTCAGA
TCTCGAGCTCAAGCTTCGAATTCTGCAGTCGACATAGAGGAACCTCCGCGAAAG
CGGTGAAGGAGAGGCGCAAGGTTAACCGCCTCAGGTTCCTCATAACAAGGCCTC
CGCGAAAGCGGTGAAGGAGCGGCACAAGGTTAACTGCCGCAGGCCTTGTATACT
CGAGAGATCGATCTCGACGGGATCCACCGG</td></tr>
<tr><td rowspan="2">mAzurite-C1-RhoBAST$_4$</td><td>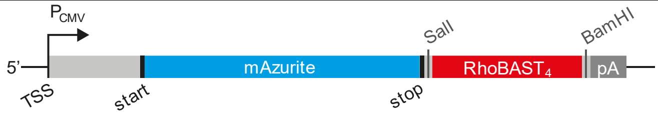
</td></tr>
<tr><td>GCTAGCGCTACCGGTCGCCACCATGGTGAGCAA [...] TACAAGTAAGGACTCAGA
TCTCGAGCTCAAGCTTCGAATTCTGCAGTCGACATAGAGGAACCTCCGCGAAAG
CGGTGAAGGAGAGGCGCAAGGTTAACCGCCTCAGGTTCCTCATAACAAGGCCTC
CGCGAAAGCGGTGAAGGAGCGGCACAAGGTTAACTGCCGCAGGCCTTGTATACT
CGACATAGGAAGACCTTCGCGAAAGCGATGAAGGAGCGGTGCAAGGTTAACCAC
CGCAGGTCTTCCATAAGCAGACCTTCGCGAAAGCGATGAAGGAGTGGCGCAAGG
TTAACCGCCACAGGTCTGCTATACTCGAGAGATCGATCTCGACGGGATCCACCG
G</td></tr>
<tr><td rowspan="2">mAzurite-C1-RhoBAST$_8$</td><td>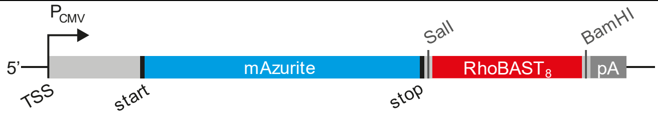
</td></tr>
<tr><td>GCTAGCGCTACCGGTCGCCACCATGGTGAGCAA [...] TACAAGTAAGGACTCAGA
TCTCGAGCTCAAGCTTCGAATTCTGCAGTCGACATAGAGGAACCTCCGCGAAAG
CGGTGAAGGAGAGGCGCAAGGTTAACCGCCTCAGGTTCCTCATAACAAGGCCTC
CGCGAAAGCGGTGAAGGAGCGGCACAAGGTTAACTGCCGCAGGCCTTGTATACT
CGACATAGGAAGACCTTCGCGAAAGCGATGAAGGAGCGGTGCAAGGTTAACCAC
CGCAGGTCTTCCATAAGCAGACCTTCGCGAAAGCGATGAAGGAGTGGCGCAAGG
TTAACCGCCACAGGTCTGCTATACTCGACATAGAGGAACCTCCGCGAAAGCGGT
GAAGGAGAGGCGCAAGGTTAACCGCCTCAGGTTCCTCATAACAAGGCCTCCGCG
AAAGCGGTGAAGGAGCGGCACAAGGTTAACTGCCGCAGGCCTTGTATACTCGAC
ATAGGAAGACCTTCGCGAAAGCGATGAAGGAGCGGTGCAAGGTTAACCACCGCA
GGTCTTCCATAAGCAGACCTTCGCGAAAGCGATGAAGGAGTGGCGCAAGGTTAA
CCGCCACAGGTCTGCTATACTCGAGAGATCGATCTCGACGGGATCCACCGG</td></tr>
<tr><td>mAzurite-C1-RhoBAST$_{16}$</td><td>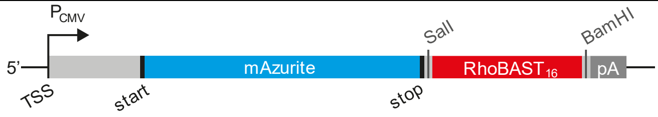
</td></tr>
</table>

	GCTAGCGCTACCGGTCGCCACCATGGTGAGCAA[…]TACAAGTAAGGACTCAGA<br>TCTCGAGCTCAAGCTTCGAATTCTGCAGTCGACATAGAGGAACCTCCGCGAAAG<br>CGGTGAAGGAGAGGCGCAAGGTTAACCGCCTCAGGTTCCTCATAACAAGGCCTC<br>CGCGAAAGCGGTGAAGGAGCGGCACAAGGTTAACTGCCGCAGGCCTTGTATACT<br>CGACATAGGAAGACCTTCGCGAAAGCGATGAAGGAGCGGTGCAAGGTTAACCAC<br>CGCAGGTCTTCCATAAGCAGACCTTCGCGAAAGCGATGAAGGAGTGGCGCAAGG<br>TTAACCGCCACAGGTCTGCTATACTCGACATAGAGGAACCTCCGCGAAAGCGGT<br>GAAGGAGAGGCGCAAGGTTAACCGCCTCAGGTTCCTCATAACAAGGCCTCCGCG<br>AAAGCGGTGAAGGAGCGGCACAAGGTTAACTGCCGCAGGCCTTGTATACTCGAC<br>ATAGGAAGACCTTCGCGAAAGCGATGAAGGAGCGGTGCAAGGTTAACCACCGCA<br>GGTCTTCCATAAGCAGACCTTCGCGAAAGCGATGAAGGAGTGGCGCAAGGTTAA<br>CCGCCACAGGTCTGCTATACTCGACATAGAGGAACCTCCGCGAAAGCGGTGAAG<br>GAGAGGCGCAAGGTTAACCGCCTCAGGTTCCTCATAACAAGGCCTCCGCGAAAG<br>CGGTGAAGGAGCGGCACAAGGTTAACTGCCGCAGGCCTTGTATACTCGACATAG<br>GAAGACCTTCGCGAAAGCGATGAAGGAGCGGTGCAAGGTTAACCACCGCAGGTC<br>TTCCATAAGCAGACCTTCGCGAAAGCGATGAAGGAGTGGCGCAAGGTTAACCGC<br>CACAGGTCTGCTATACTCGACATAGAGGAACCTCCGCGAAAGCGGTGAAGGAGA<br>GGCGCAAGGTTAACCGCCTCAGGTTCCTCATAACAAGGCCTCCGCGAAAGCGGT<br>GAAGGAGCGGCACAAGGTTAACTGCCGCAGGCCTTGTATACTCGACATAGGAAG<br>ACCTTCGCGAAAGCGATGAAGGAGCGGTGCAAGGTTAACCACCGCAGGTCTTCC<br>ATAAGCAGACCTTCGCGAAAGCGATGAAGGAGTGGCGCAAGGTTAACCGCCACA<br>GGTCTGCTATACTCGAGAGATCGATCTCGACGGGATCCACCGG

Supplementary Table 4. List of used single-stranded DNA oligonucleotides.

Plasmid	Comment	Sequence
pAV-U6+27-Tornado-RhoBAST	Insert	AAGAAGCGGCCGCACCTCCGCGAAAGCGGTGAAGGAGAGGCGC
pAV-U6+27-Tornado-RhoBAST	Insert	TTCTTCCGCGGCCACACCTGAGGCGGTTAACCTTGCGCCTCTCCTTCAC
pAV-U6+27-Tornado-Pepper	Insert	AAGAAGCGGCCGCCCCAATCGTGGCGTGTCGGCCTGCTTCGGCAG
pAV-U6+27-Tornado-Pepper	Insert	TTCTTCCGCGGCCACCCGGCGCCAGTGCCTGCCGAAGCAGGCCGAC
pAV-U6+27-Tornado-Pepper-RhoBAST	Insert	AGTGCGGCCGCCCCAATCGTGGCGTGTCGGCCTGCAACCTCCG
pAV-U6+27-Tornado-Pepper-RhoBAST	Insert	CGACCGCGGCCACCCGGCGCCAGTGCCTGCAACCTGAGGCGGTTAACCTTG
pAV-U6+27-Tornado-RhoBAST-MS2	Insert	AGTGCGGCCGCACCTCGGCGGCCAACATGAGGATCACCC
pAV-U6+27-Tornado-RhoBAST-MS2	Insert	CGACCGCGGCCACACCTGAGGCGGTTAACCTTGCGCCTCTCCTTC
pAV-U6+27-Tornado-[RhoBAST-MS2]2	Insert	AGTGCGGCCGCTTGCCATGTGTATCGGAAGGCCTCCGCGCCCAGGTG
pAV-U6+27-Tornado-[RhoBAST-MS2]2	Insert	CGACCGCGGCCACTTGCCATGAATGATCCGGAACCTGAGGCGG
pcDNA5-FRT/TO-NLS-dCas9-NLS-GFP	Insert	AGACTCGAGCTTACTTGTACAGCTCGTCCATG
pcDNA5-FRT/TO-NLS-dCas9-NLS-GFP	Insert	CTCGGATCCTCGCCACCATGGCTAG
pcDNA3-HaloTag7-Sam68	Vector	CATGGTGGCGGCGAAGCTTGGGTCTCCC
pcDNA3-HaloTag7-Sam68	Vector	GGTTCCGGCTCAGGATCTAGAGGATCCCAGCGC

pcDNA3-HaloTag7-Sam68	Insert	GACCCAAGCTTCGCCGCCACCATGGGATCCGAAATCGGTACTGGC
pcDNA3-HaloTag7-Sam68	Insert	GATCCTCTAGATCCTGAGCCGGAACCACCGGAAATCTCCAGAGTAGAC
pcDNA5-FRT-*Localization-Seq*-stdMCP-stdGFP	Vector (for N- and C-terminal loc. seq.)	CAAGCTTAAGTTTAAACGCTAGCCAGC
pcDNA5-FRT-*Localization-Seq*-stdMCP-stdGFP	Vector (for N- and C-terminal loc. seq.)	TCTAGAGGGCCCGTTTAAACC
pcDNA5-FRT-*Localization-Seq*-stdMCP-stdGFP	Insert (stdMCP-stdGFP) for N-terminal loc. seq.	CTGTTATTAATTAACGCTTCTAACTTTACTCAGTTCG
pcDNA5-FRT-*Localization-Seq*-stdMCP-stdGFP	Insert (stdMCP-stdGFP) for N-terminal loc. seq.	GCGGGTTTAAACGGGCCCTCTAGATTATTTGTACAATTCATCCATACCATGGGTAATAC
pcDNA5-FRT-*Localization-Seq*-stdMCP-stdGFP	Insert (stdMCP-stdGFP) for C-terminal loc. seq.	GGCTAGCGTTTAAACTTAAGCTTGGCCACCATGGCTTCTAACTTTACTCAGTTCGTTCTC
pcDNA5-FRT-*Localization-Seq*-stdMCP-stdGFP	Insert (stdMCP-stdGFP) for C-terminal loc. seq.	TTTGTACAATTCATCCATACCATGGGTAATAC
pcDNA5-FRT-TOMM20-stdMCP-stdGFP	Insert (TOMM20)	GGCTAGCGTTTAAACTTAAGCTTGGCCACCATGGGTCGGAACAGCGCCATCGCCGCGGG
pcDNA5-FRT-TOMM20-stdMCP-stdGFP	Insert (TOMM20)	GTTAGAAGCGTTAATTAATAACAGGAAGTTGGGGTCACTTCGTCTTTTGCGGTCAAAGTA
pcDNA5-FRT-TOMM20-stdMCP-stdGFP	Insert (TOMM20)	CGCCGCGGGCGTGTGCGGTGCCCTCTTCATAGGGTACTGCATCTACTTTGACCGCAAAAG
mAzurite-RhoBASTn	Vector	CTGTACAAGTAAGGACTCAGATCTCGAGC
mAzurite-RhoBASTn	Vector	CTCGTCCATGCCGTGAGT
mAzurite-RhoBASTn	Insert	GGCGTAAGAATTCAGAGTCGAC
mAzurite-RhoBASTn	Insert	GGTGGATCCCGTCGAGATCGATCTCTCGAG

Supplementary Table 5. List of used double-stranded gene fragments.

Plasmid	Sequence
pAV-U6-centromere-sgRNA	GCCGGATCCAAGGTCGGGCAGGAAGAGGGCCTATTTCCCATGATTCCTTCATATTTGCATATACGATACAAGGCTGTTAGAGAGATAATTGGAATTAATTTGACTGTAAACACAAAGATATTAGTACAAAATACGTGACGTAGAAAGTAATAATTTCTTGGGTAGTTTGCAGTTTTAAAATTATGTTTTAAAATGGACTATCATATGCTTACCGTAACTTGAAAGTATTTCGATTTCTTGGCTTTATATATCTTGTGGAAAGGACGAAACACCGAATCTGCAAGTGGATATTGTTTGAGAGCTAGAAATAGCAAGTTCAAATAAGGCTAGTCCGTTATCAACTTGAAAAAGTGGCACCGAGTCGGTGCTTTTTTTGTTTTACTCGAGCCGAGGAC
pAV-U6-centromer-sgRNA- $RhoBAST_{tl}$	GCCGGATCCAAGGTCGGGCAGGAAGAGGGCCTATTTCCCATGATTCCTTCATATTTGCATATACGATACAAGGCTGTTAGAGAGATAATTGGAATTAATTTGACTGTAAACACAAAGATATTAGTACAAAATACGTGACGTAGAAAGTAATAATTTCTTGGGTAGTTTGCAGTTTTAAAATTATGTTTTAAAATGGACTATCATATGCTTACCGTAACTTGAAAGTATTTCGATTTCTTGGCTTTATATATCTTGTGGAAAGGACGAAACACCGAATCTGCAAGTGGATATTGTTTGAGAGCTAGGAACCTCCGCGAAAGCGGTGAAGGAGAGGCGCAAGGTTAACCGCCTCAGGTTCCTAGCAAGTTCAAATAAGGCTAGTCCGTTATCAACTTGAAAAAGTGGCACCGAGTCGGTGCTTTTTTTGTTTTACTCGAGCCGAGGAC

pAV-U6-centromer-sgRNA-RhoBAST$_{sl}$	GCCGGATCCAAGGTCGGGCAGGAAGAGGGCCTATTTCCCATGATTCCTTCATATTT<br>GCATATACGATACAAGGCTGTTAGAGAGATAATTGGAATTAATTTGACTGTAAACA<br>CAAAGATATTAGTACAAAATACGTGACGTAGAAAGTAATAATTTCTTGGGTAGTTT<br>GCAGTTTTAAAATTATGTTTTAAAATGGACTATCATATGCTTACCGTAACTTGAAA<br>GTATTTCGATTTCTTGGCTTTATATATCTTGTGGAAAGGACGAAACACCGAATCTG<br>CAAGTGGATATTGTTTGAGAGCTAGAAATAGCAAGTTCAAATAAGGCTAGTCCGTT<br>ATCAACTTGGAACCTCCGCGAAAGCGGTGAAGGAGAGGCGCAAGGTTAACCGCCTC<br>AGGTTCCAAGTGGCACCGAGTCGGTGCTTTTTTGTTTTACTCGAGCCGAGGAC
mAzurite-C1-RhoBAST$_2$	GCCGGATCCAAGGTCGGGCAGGAAGAGGGCCTATTTCCCATGATTCCTTCATATTT<br>GCATATACGATACAAGGCTGTTAGAGAGATAATTGGAATTAATTTGACTGTAAACA<br>CAAAGATATTAGTACAAAATACGTGACGTAGAAAGTAATAATTTCTTGGGTAGTTT<br>GCAGTTTTAAAATTATGTTTTAAAATGGACTATCATATGCTTACCGTAACTTGAAA<br>GTATTTCGATTTCTTGGCTTTATATATCTTGTGGAAAGGACGAAACACCGAATCTG<br>CAAGTGGATATTGTTTGAGAGCTAGGAACCTCCGCGAAAGCGGTGAAGGAGAGGCG<br>CAAGGTTAACCGCCTCAGGTTCCTAGCAAGTTCAAATAAGGCTAGTCCGTTATCAA<br>CTTAAGGCCTCCGCGAAAGCGGTGAAGGAGCGGCACAAGGTTAACTGCCGCAGGCC<br>TTAAGTGGCACCGAGTCGGTGCTTTTTTGTTTTACTCGAGCCGAGGAC
pcDNA5-FRT-stdMCP-stdGFP-Sec61β	CATGGTATGGATGAATTGTACAAATCCGGACTCAGATCTGGCTCCAGCGCAGGCAG<br>CGCATCCGGCGGAAGCGGAAGCCCTGGTCCGACCCCCAGTGGCACTAACGTGGGAT<br>CCTCAGGGCGCTCTCCCAGCAAAGCAGTGGCCGCCCGGGCGGCGGGATCCACTGTC<br>CGGCAGAGGAAAAAATGCCAGCTGTGGGACAAGGAGTGCAGGCCGCACAACCTCGGC<br>AGGCACCGGGGGGATGTGGCGATTCTACACAGAAGATTCACCTGGGCTCAAAGTTG<br>GCCCTGTTCCAGTATTGGTTATGAGTCTTCTGTTCATCGCTTCTGTATTTATGTTG<br>CACATTTGGGGCAAGTACACTCGTTCGTAGTCTAGAGGGCCCGTTTAAACCCGC
pcDNA5-FRT-H2B-stdMCP-stdGFP	GCTAGCGTTTAAACTTAAGCTTGGCCACCATGCCAGAGCCAGCGAAGTCTGCTCCC<br>GCCCCGAAAAAGGGCTCCAAGAAGGCGGTGACTAAGGCGCAGAAGAAAGGCGGCAA<br>GAAGCGCAAGCGCAGCCGCAAGGAGAGCTATTCCATCTATGTGTACAAGGTTCTGA<br>AGCAGGTCCACCCTGACACCGGCATTTCGTCCAAGGCCATGGGCATCATGAATTCG<br>TTTGTGAACGACATTTTCGAGCGCATCGCAGGTGAGGCTTCCCGCCTGGCGCATTA<br>CAACAAGCGCTCGACCATCACCTCCAGGGAGATCCAGACGGCCGTGCGCCTGCTGC<br>TGCCTGGGGAGTTGGCCAAGCACGCCGTGTCCGAGGGTACTAAGGCCATCACCAAG<br>TACACCAGCGCTAAGCTGTTATTAATTAACGCTTCTAAC

Supplementary Note 1:

Limitations of fluorophore-quencher conjugates due to residual quenching

Initially, we aimed to enhance the brightness of the established FLAP RhoBAST:TMR-DN[1] by increasing the fluorescence quantum yield, Φ_F, of the complex. Accordingly, we synthesized the fluorophore-quencher conjugate JF_{549}-DN, in which the dimethylamino groups of the TMR fluorophore were replaced by strained azetidine heterocycles (Supplementary Fig. N1a,b). This small structural modification is known to substantially increase the quantum yield of tetramethyl rhodamines from about 0.4 to 0.9 *via* suppression of the twisted intramolecular charge transfer (TICT) non-radiative decay channel.[2]

The prepared JF_{549}-DN probe displayed similar binding properties to RhoBAST than TMR-DN, specifically, an equilibrium dissociation coefficient K_D = 15 nM and a 30-fold fluorescence turn-on (Supplementary Fig. N1c-e, Supplementary Table N1). Surprisingly, the RhoBAST:JF_{549}-DN (Φ_F = 0.55) complex had a similar low quantum yield as RhoBAST:TMR-DN (Φ_F = 0.57), despite TICT inhibition. Thus, we hypothesized that the fluorescence quenching by the conjugated DN is not completely abolished through the binding of RhoBAST. Indeed, the quantum yield of RhoBAST:TMR (without quencher moiety) was very high (Φ_F =0.92), whereas the free dye has a quantum yield of 0.48.

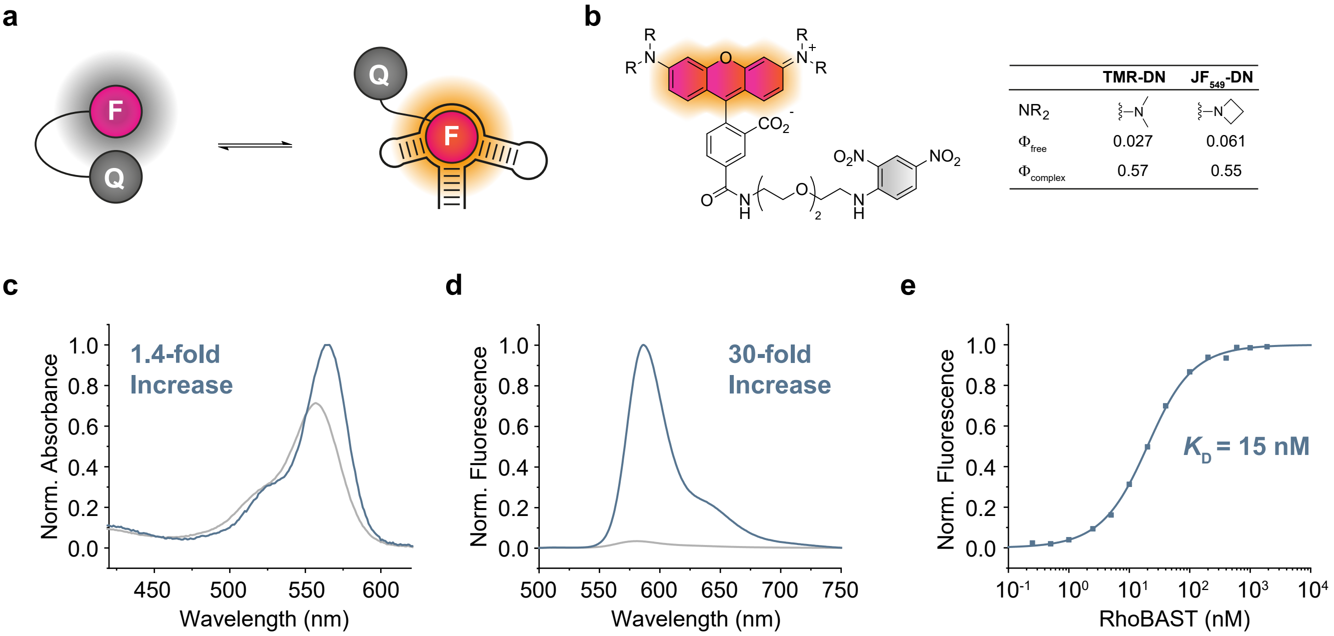

Supplementary Figure N1: Properties of the fluorophore-quencher conjugate JF_{549}-DN. a) Schematic illustration of the light-up mechanism of fluorophore-quencher conjugates. Binding to an aptamer leads to disruption of the fluorophore-quencher interaction and consequently a fluorescence increase. **b)** Chemical structures and fluorescence quantum yields of TMR-DN and JF_{549}-DN. **c)** Normalized absorption spectra of JF_{549}-DN in the presence (blue line) and absence (grey line) of RhoBAST. **d)** Normalized fluorescence emission spectra of JF_{549}-DN in the presence (blue line) and absence (grey line) of RhoBAST. Absorbance and fluorescence measurements were performed in ASB at 25 °C using 1 µM dye and 5 µM RhoBAST. **e)** Binding isotherm of JF_{549}-DN using RhoBAST. Measurements were performed in ASB supplemented with 0.05% Tween 20 at 25 °C using 10 nM probe.

Supplementary Table N1. Properties of fluorophore-quencher conjugates and the corresponding RhoBAST:dye complexes. The excitation wavelength (λ_{ex}), emission wavelength (λ_{em}), extinction coefficient (ε), fluorescence quantum yield (Φ_F), and fluorescence turn-on (1 µM probe, 5 µM RhoBAST) were measured in ASB at 25 °C. Absolute quantum yields were measured for rhodamines; values for RhoBAST:dye complexes were determined relative to sulforhodamine 101. The brightness was calculated as the product of ε and Φ_F. K_D values were measured in ASB supplemented with 0.05% Tween 20 at 25 °C using 10 nM probe.

	free dye					RhoBAST:dye complex						
	λ_{ex} [nm]	λ_{em} [nm]	ε [$M^{-1}cm^{-1}$]	Φ_F	Brightness [$M^{-1}cm^{-1}$]	λ_{ex} [nm]	λ_{em} [nm]	ε [$M^{-1}cm^{-1}$]	Φ_F	Brightness [$M^{-1}cm^{-1}$]	Turn-on [n-fold]	K_D [nM]
TMR	549	573	75000 ± 3000	0.48	36000	560	581	80000 ± 3000	0.92 ± 0.02	74000	2.9 ± 0.2	12 ± 1
5C-TMR	551	578	77000 ± 1000	0.43	33000	563	587	-	-	-	-	32 ± 6
TMR-DN[1]	553	582	64000	0.027	1700	564	590	96000	0.57 ± 0.04	55000	26	15
5C-JF_{549}	552	578	81000 ± 1000	0.92	75000	564	584	-	-	-	-	35 ± 1
JF_{549}-DN	554	581	62000 ± 1000	0.061 ± 0.002[a]	3800	565	586	88000 ± 4000	0.55 ± 0.02	48000	30 ± 10	15 ± 1

[a] Quantum yield was determined relative to sulforhodamine 101.

Supplementary Note 2:

Shifting the open-closed equilibrium by increasing the electrophilicity of the xanthene

The electrophilicity of the xanthene core can be increased by the introduction of fluorine substituents at the 2′- and 7′-position and/or the use of amine substituents bearing electron-withdrawing groups (Supplementary Fig. N2a).[3-7] Notably, the decreased electron density does not only lead to a shift of the open-closed equilibrium to the non-fluorescent spirolactone form, but also to an undesired hypsochromic shift of the excitation and emission maxima,[3] making the dye less suitable for excitation with a common 561 nm laser. However, due to the high brightness, photostability and structural similarity to the high-affinity binder 5C-JF$_{549}$ (K_D = 35 nM), we decided to synthesize the fluorinated version 5C-JF$_{525}$ and test its applicability as a ligand for RhoBAST.[3]

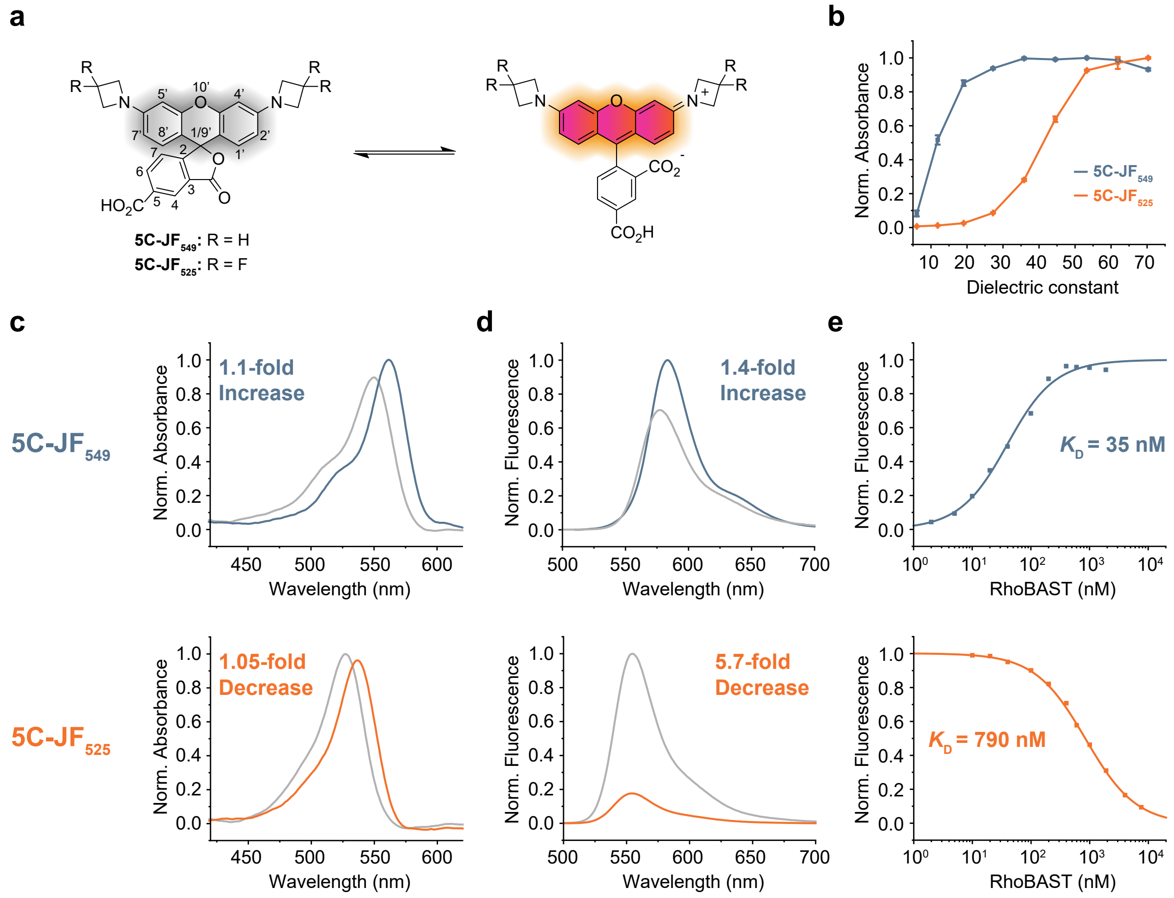

Supplementary Figure N2. Properties of azetidinyl rhodamines. a) Chemical structure and open-closed equilibrium of 5C-JF$_{549}$ and 5C-JF$_{525}$. **b)** Absorption of 5C-JF$_{549}$ (5 µM) and 5C-JF$_{525}$ (5 µM) as a function of the dielectric constant of the solvent mixture (water/dioxane). The absorbance was normalized to the highest measured point of each derivative. **c)** Normalized absorption spectra of 5C-JF$_{549}$ and 5C-JF$_{525}$ in the presence (colored lines) and absence (grey lines) of RhoBAST. **d)** Normalized fluorescence emission spectra of 5C-JF$_{549}$ and 5C-JF$_{525}$ in the presence (colored lines) and absence (grey lines) of RhoBAST. Absorbance and fluorescence spectra were measured in ASB at 25 °C using 1 µM dye and 5 µM RhoBAST. **e)** Binding isotherms of 5C-JF$_{549}$ and 5C-JF$_{525}$ using RhoBAST, measured in ASB supplemented with 0.05% Tween 20 at 25 °C using 10 nM probe.

First, the D_{50} values of azetidinyl rhodamine 5C-JF$_{549}$ and 5C-JF$_{525}$ were measured to investigate the position of the open-closed equilibrium (Supplementary Fig. N2a,b). Compared to the non-fluorinated

parent dye 5C-JF_{549} (D_{50} = 12), 5C-JF_{525} displayed the desired shift of the equilibrium towards the spirolactone form (D_{50} = 41). However, despite the structural similarity, 5C-JF_{525} bound to RhoBAST with a substantially lower affinity (K_D = 793 nM) than 5C-JF_{549} (K_D = 35 nM) and displayed a fluorescence decrease instead of the desired light-up (Supplementary Fig. N2c-e). A similar turn-off behavior was previously observed for the xanthene dyes 9-aminoacridine and oxazine 1, which possess a relatively electron-poor conjugated system, upon binding to sulforhodamine B-binding aptamer SRB-2, the predecessor to RhoBAST.[1] Consequently, we reasoned that reducing the electron density in the xanthene system would not lead to the desired advanced fluorogenic probes for the RhoBAST system.

Supplementary Note 3:

Chemical Synthesis

All commercial reagents were purchased from suppliers Sigma-Aldrich, ABCR, Acros, TCI and Alfa Aesar and used without further purification. Moisture and/or oxygen-sensitive reactions were carried out under an argon atmosphere using standard Schlenk techniques.

Silica column chromatography was performed using silica gel (high-quality, pore size 60 Å, 40-63 µm particle size) purchased from Sigma-Aldrich. All fluorophores were purified *via* reverse phase High Performance Liquid Chromatography (HPLC) before use in live-cell or *in vitro* experiments. HPLC was performed on an AGILENT 1100 Series HPLC system equipped with a multi wavelength detector and a fraction collector using a Phenomenex Luna 5 µm C-18(2) 100 Å (250 x 21.2 mm) column. All purification runs were carried out with a constant flow rate of 6 ml/min using mixtures of water and acetonitrile (containing 0.1% TFA each) as solvent system. Appropriate fractions were freeze-dried using a lyophilizer Alpha 2-4 LDplus (Christ).

Nuclear Magnetic Resonance Spectroscopy (NMR) spectra were recorded on Varian Mercury Plus 300 or Mercury Plus 500 systems and analyzed by MestReNova (9.0.1). Deuterated solvents were purchased from Euriso-Top. Chemical shifts (*δ*) are given in ppm (with respect to tetramethylsilane) and coupling constants (*J*) in Hz. All recorded ^{1}H and ^{13}C spectra were referenced to the protio impurity or the ^{13}C signal of the deuterated solvent. ^{13}C measurements were recorded as APT spectra.

High resolution mass spectra (HR-MS) were measured on a Bruker micrOTOF QII-ESI system using sodium formiate as internal calibrant. The reported mass to charge ratios (m/z) refer to the isotopic peak with the highest intensity.

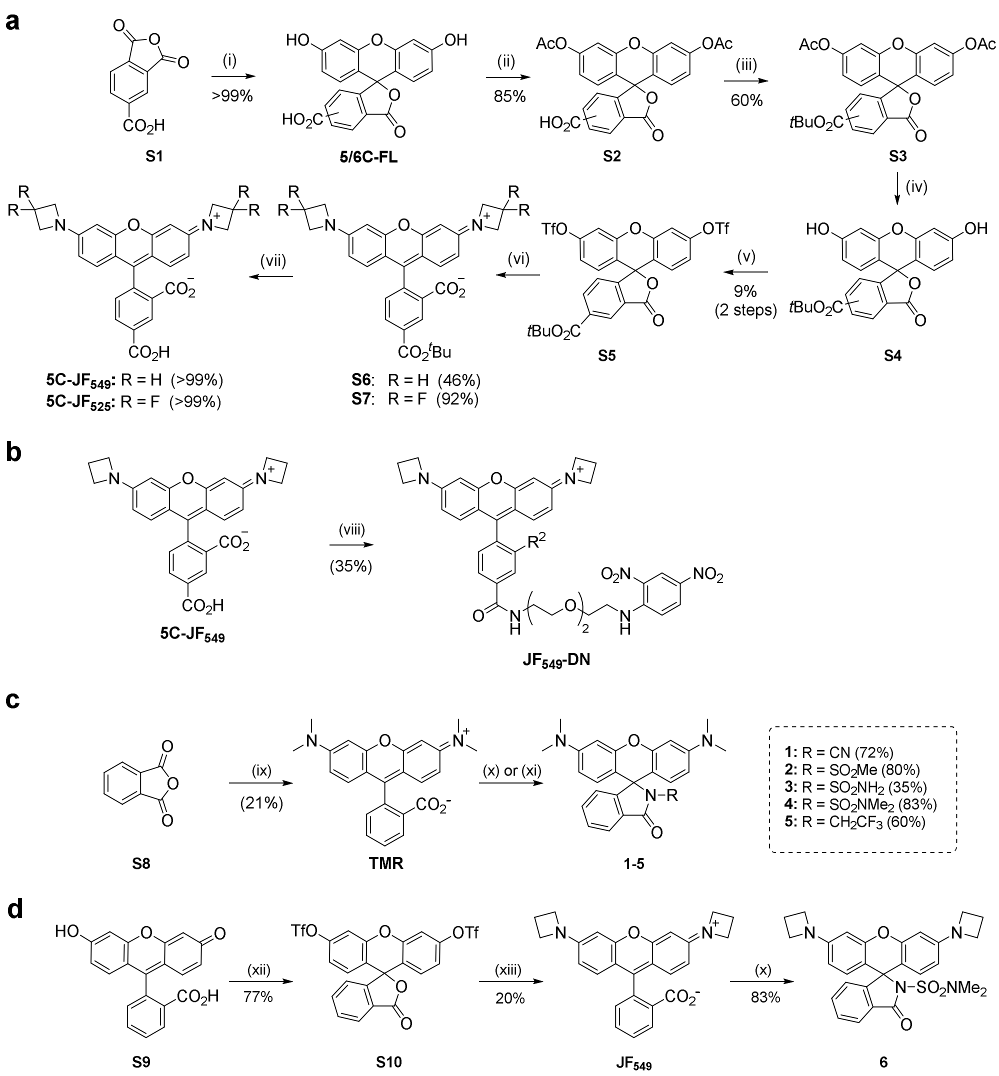

Supplementary Figure N3: Synthesis of rhodamine dyes. (i) Resorcinol, $MeSO_3H$; (ii) Acetic anhydride; (iii) Dimethylformamide di-tert-butyl acetal, toluene; (iv) NaOH, methanol, THF; (v) Trifluoromethane sulfonic anhydride, pyridine, DCM; (vi) Azetidine/3,3-difluoroazetidine hydrochloride, $Pd_2(dba)_3$, XPhos, Cs_2CO_3, dioxane; (vii) TFA, DCM. (viii) TSTU, **DN-NH$_2$**, DIPEA, DMF. (ix) 3-Dimethylaminophenol; (x) amine, EDC, DMAP (for **1**,**2**,**4**,**5**,**6**); (xi) $POCl_3$, amine, DIPEA (for **3**); (xii) Tf_2O, pyridine; (xiii) azetidine, Pd_2dba_3, XPhos, Cs_2CO_3.

5/6-Carboxy fluorescein (5/6-C-FL)

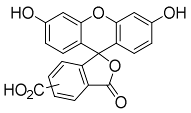

$C_{21}H_{12}O_7$
376.32 g/mol

Trimellitic anhydride (6.00 g, 31.2 mmol, 1.00 eq) and resorcinol (6.88 g, 62.5 mmol, 2.00 eq) were suspended in methanesulfonic acid (40 mL). The mixture was stirred at 120 °C for 2 h. The solution was poured into ice water (500 mL) and the resulting precipitate was isolated *via* filtration. The precipitate was washed with water (2 × 100 mL) and dissolved in aqueous 1.25 M NaOH (200 mL). The solution was reacidified with conc. HCl (~30 mL) to pH 2-3 and the resulting precipitate was isolated by filtration and washed with water (2 × 100 mL). Next, the residue was dried in an oven at 160 °C for 10 h to yield the product as an orange powder (11.7 g, >99%). The recorded spectrum was in accordance with literature.[8]

^{1}H NMR (300 MHz, $(CD_3)_2SO$): δ = 8.41 – 8.37 (m, 1H, 5-isomer), 8.29 (dd, *J* = 8.1, 1.5 Hz, 1H, 5-isomer), 8.22 (dd, *J* = 8.0, 1.3 Hz, 1H, 6-isomer), 8.14 – 8.07 (m, 1H, 6-isomer), 7.67 – 7.61 (m, 1H, 6-isomer), 7.42 – 7.35 (m, 1H, 5-isomer), 6.74 – 6.68 (m, 4H, 5-isomer, 6-isomer), 6.64 – 6.51 (m, 8H, 5-isomer, 6-isomer) ppm.

5/6-Carboxy fluorescein diacetate (S2)

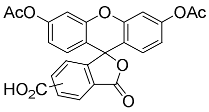

$C_{25}H_{16}O_9$
460.39 g/mol

First, 5/6-Carboxy fluorescein (1.50 g, 3.99 mmol, 1.00 eq) was suspended in Ac_2O (10 mL) and the mixture was refluxed for 4 h. Next, water was added until a white precipitate was obtained. The supernatant was discarded and the obtained solid was dissolved in EtOAc (50 mL). The organic phase was washed with brine (20 mL), dried over magnesium sulfate, filtered and the solvent was removed under reduced pressure. The desired product was obtained as a light yellowish solid (1.41 g, 85%). The recorded spectrum was in accordance with literature.[9]

^{1}H NMR (300 MHz, $(CD_3)_2SO$): δ = 8.46 – 8.43 (m, 1H, 5-isomer), 8.32 (dd, *J* = 8.1, 1.5 Hz, 1H, 5-isomer), 8.27 (dd, *J* = 8.0, 1.3 Hz, 1H, 6-isomer), 8.17 (d, *J* = 7.8 Hz, 1H, 6-isomer), 7.84 (s, 1H, 6-isomer), 7.55 (d, *J* = 8.0 Hz, 1H, 5-isomer), 7.30 (d, *J* = 1.3 Hz, 3H, 5/6-isomer), 6.95 (m, 7H, 5/6-isomer), 2.29 (s, 12H, 5/6-isomer) ppm.

5/6-*tert*-Butoxycarbonyl fluorescein diacetate (S3)

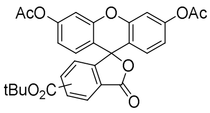

$C_{25}H_{16}O_9$
516.50 g/mol

A *Schlenk* flask was charged with 5/6-Carboxy fluorescein diacetate (1.20 g, 2.61 mmol, 1.00 eq) and evacuated/backfilled with Argon three times. The solid was suspended in anhydrous toluene (10 mL) and *N*,*N*-Dimethylformamide di-*tert*-butylacetal (2.50 mL, 2.12 g, 10.4 mmol, 4.00 eq) was added to the suspension. The mixture was stirred at 85 °C overnight. Next, the reaction mixture was diluted with toluene (50 mL) and washed with saturated aqueous $NaHCO_3$ solution (2 × 30 mL). The organic phase

was dried over magnesium sulfate, filtered and the solvent removed under reduced pressure to yield **S3** as light yellowish solid (773 mg, 57%).

^{1}H NMR (300 MHz, $CDCl_3$): δ = 8.62 (dd, J = 1.4, 0.7 Hz, 1H, 5-isomer), 8.31 (dd, J = 8.0, 1.5 Hz, 1H, 5-isomer), 8.26 (dd, J = 8.0, 1.3 Hz, 1H, 6-isomer), 8.07 (dd, J = 8.0, 0.7 Hz, 1H, 6-isomer), 7.73 (dd, J = 1.2, 0.8 Hz, 1H, 6-isomer), 7.23 (m, 1H, 5-isomer), 7.13 – 7.10 (m, 4H, 5/6-isomer), 6.85 – 6.77 (m, 8H, 5/6-isomer), 2.32 (m, 12H, 5/6-isomer), 1.64 (s, 9H, 5-isomer), 1.56 (s, 9H, 5/6-isomer) ppm.

^{13}C NMR (75 MHz, $CDCl_3$): δ = 169.0, 168.9, 168.4, 168.4, 164.0, 164.0, 156.2, 152.8, 152.4, 152.3, 151.7, 151.7, 138.8, 136.4, 134.6, 131.4, 129.4, 129.2, 129.1, 129.0, 128.4, 126.8, 126.6, 125.4, 125.3, 125.2, 124.3, 118.0, 116.0, 115.9, 110.7, 110.6, 83.0, 82.6, 82.2, 81.9, 28.3, 28.1, 21.3 ppm.

MS (HR-ESI, pos): meas. m/z = 539.1313, calc. m/z = 539.1313 for $C_{25}H_{16}NaO_9$ $[M+Na]^+$.

5-*tert*-Butoxycarbonyl fluorescein ditriflate (S5)

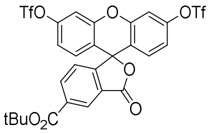

$C_{27}H_{18}F_6O_{11}S_2$
696.54 g/mol

First, 5/6-carboxyfluorescein diacetate *tert*-butyl ester (1.62 g, 3.14 mmol, 1.00 eq) was dissolved in a 1:1 mixture of THF and MeOH (25 mL) and aqueous 1 M NaOH (6 mL) was added. The mixture was stirred at room temperature for 1 h. The solution was acidified with aqueous 1 M HCl (7 mL) and diluted with water (30 mL). The aqueous phase was extracted with EtOAc (2 × 60 mL) and the combined organic layers were washed with brine (40 mL). The organic layer was dried over $MgSO_4$, filtered and the solvent removed *in vacuo*. The resulting orange solid was taken up in DCM (15 mL) and cooled to 0 °C. Pyridine (2.02 mL, 1.98 g, 25.1 mmol, 8.00 eq) was added to the suspension, followed by Tf_2O (2.11 mL, 3.54 g, 12.6 mmol, 4.00 eq). The mixture was allowed to warm up to room temperature and was stirred for an additional 1 h. The resulting red solution was diluted with water (30 mL) and extracted with DCM (2 × 60 mL). The combined organic layers were dried over magnesium sulfate, filtered and put on celite. The crude mixture was purified by silica column chromatography (cyclohexane:EtOAc = 10:0 → 7:1). The isomerically pure product was obtained as an off-white solid (200 mg, 9%).

^{1}H NMR (500 MHz, $CDCl_3$): δ = 8.66 (s, 1H), 8.36 (dd, J = 8.0, 1.2 Hz, 1H), 7.31 (d, J = 2.4 Hz, 2H), 7.24 (d, J = 8.0 Hz, 1H), 7.03 (dd, J = 8.8, 2.4 Hz, 2H), 6.94 (d, J = 8.8 Hz, 2H), 1.64 (s, 9H) ppm.

^{13}C NMR (126 MHz, $CDCl_3$): δ = 167.8, 163.7, 155.4, 151.4, 150.5, 136.9, 135.3, 130.0, 127.2, 126.0, 123.9, 120.9, 118.9, 118.0, 111.0, 82.9, 80.3 28.3 ppm.

MS (HR-ESI, pos): meas. m/z = 719.0088, calc. m/z = 719.0087 for $C_{27}H_{18}F_6NaO_{11}S_2$ $[M+Na]^+$.

General procedure for *Buchwald Hartwig* coupling of ditriflates (GP1)

The following procedure is exemplary for the *Buchwald-Hartwig amination* of fluorescein ditriflates to the corresponding azetidine-substituted rhodamines:

A reaction vessel was charged with fluorescein ditriflate (1.00 eq), Cs_2CO_3 (2.80 eq), $Pd_2(dba)_3$ (0.20 eq) and 2-dicyclohexyl-phosphino-2′,4′,6′-triisopropylbiphenyl (XPhos, 0.30 eq), sealed, evacuated and backfilled with argon (3×). The solids were suspended in dry dioxane (35 mM solution). Azetidine or 3,3-difluoroazetidine hydrochloride (2.40 eq) was added and the mixture

was stirred at 100 °C overnight. The mixture was diluted with MeOH and the solvent was evaporated under reduced pressure. The crude product was purified by silica column chromatography.

5-*tert*-Butoxycarbonyl Janelia Fluor 549 (S6)

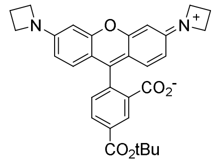

$C_{31}H_{30}N_2O_5$
510.59 g/mol

Rhodamine **S6** was synthesized from ditriflate **S5** and azetidine according to general procedure 1. The crude mixture was purified by silica column chromatography ($CHCl_3$:2 M NH_3 in MeOH = 10:0 → 9:1) and the product was obtained as violet solid (33.8 mg, 46%).

^{1}H NMR (300 MHz, $CDCl_3$): *δ* = 8.60 (s, 1H), 8.25 (dd, *J* = 8.0, 1.3 Hz, 1H), 7.21 (d, *J* = 8.0 Hz, 1H), 6.56 (d, *J* = 8.6 Hz, 2H), 6.20 (d, *J* = 1.9 Hz, 2H), 6.09 (dd, *J* = 8.6, 2.0 Hz, 2H), 3.93 (t, *J* = 7.3 Hz, 8H), 2.39 (p, *J* = 7.2 Hz, 4H), 1.63 (s, 9H) ppm.

^{13}C NMR (75 MHz, $CDCl_3$): *δ* = 169.0, 164.5, 155.0, 154.0, 153.2, 135.3, 133.8, 129.1, 128.6, 126.9, 124.8, 108.2, 107.7, 97.4, 82.3, 70.7, 52.2, 28.3, 16.7 ppm.

MS (HR-ESI, pos): meas. *m/z* = 511.2225, calc. *m/z* = 511.2227 for $C_{31}H_{31}N_2O_5$ $[M+H]^+$.

5-*tert*-Butoxycarbonyl Janelia Fluor 525 (S7)

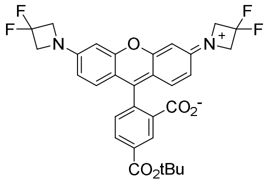

$C_{31}H_{26}F_4N_2O_5$
582.55 g/mol

Rhodamine **S7** was synthesized from ditriflate **S5** and 3,3-difluoroazetidine hydrochloride according to general procedure 1. The crude mixture was purified by silica column chromatography ($CHCl_3$:MeOH = 10:0 → 60:1) and the product was obtained as violet solid (80.5 mg, 92%).

^{1}H NMR (500 MHz, $CDCl_3$): *δ* = 8.65 – 8.54 (m, 1H), 8.28 (dd, *J* = 8.0, 1.4 Hz, 1H), 7.20 (d, *J* = 8.0 Hz, 1H), 6.61 (d, *J* = 8.6 Hz, 2H), 6.30 (d, *J* = 2.3 Hz, 2H), 6.16 (dd, *J* = 8.6, 2.3 Hz, 2H), 4.25 (t, $^3J_{HF}$ = 11.7 Hz, 8H), 1.64 (s, 9H) ppm.

^{19}F NMR (282 MHz, $CDCl_3$) *δ* = −99.5 (p, $^3J_{HF}$ = 11.7 Hz) ppm.

^{13}C NMR (126 MHz, $CDCl_3$): *δ* = 168.8, 164.3, 156.6, 152.5, 151.5 (t, $^4J_{CF}$ = 2.77 Hz), 136.0, 134.2, 129.3, 127.5, 126.6, 124.1, 115.7 (t, $^1J_{CF}$ = 275 Hz), 109.0, 108.9, 99.4, 84.2, 82.5, 63.4 (t, $^2J_{CF}$ = 26.5 Hz), 28.3 ppm.

MS (HR-ESI, pos): meas. *m/z* = 605.1670; calc. *m/z* = 605.1770 for $C_{31}H_{26}F_4N_2NaO_5$ $[M+Na]^+$.

General procedure for the deprotection of *tert*-butyl ester of rhodamines (GP2)

The following procedure is exemplary for the deprotection of *tert*-butyl esters of azetidine-substituted rhodamines.

The rhodamine was dissolved in DCM (1.5 mL) and TFA (150 µL) was added. The mixture was stirred at room temperature for 6 h. The resulting solution was diluted with benzene upon which a dark solid precipitated. The solvents were removed under reduced pressure and the resulting crude product was co-evaporated with MeOH (3×).

5-Carboxy Janelia Fluor 549 (5C-JF$_{549}$)

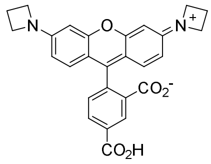

$C_{27}H_{22}N_2O_5$
454.48 g/mol

The deprotected **5C-JF$_{549}$** fluorophore was synthesized from **S6** according to general procedure 2. The TFA salt of the product was obtained as dark violet solid (32.9 mg, 98%).

^{1}H NMR (500 MHz, CD_3OD): δ = 8.88 (d, J = 1.4 Hz, 1H), 8.39 (dd, J = 7.9, 1.6 Hz, 1H), 7.48 (d, J = 7.9 Hz, 1H), 7.06 (d, J = 9.2 Hz, 2H), 6.60 (dd, J = 9.2, 2.1 Hz, 2H), 6.53 (d, J = 2.0 Hz, 2H), 4.30 (t, J = 7.6 Hz, 8H), 2.56 (p, J = 7.6 Hz, 4H) ppm.

^{13}C NMR (126 MHz, CD_3OD): δ = 168.2, 167.8, 160.5, 158.8, 158.1, 139.3, 134.4, 134.1, 133.8, 133.3, 132.2, 131.8, 114.7, 113.6, 95.1, 52.8, 16.8 ppm.

MS (HR-ESI, pos): meas. *m/z* = 455.1612, calc. *m/z* = 455.1601 for $C_{27}H_{22}N_2O_5$ $[M+H]^+$.

5-Carboxy Janelia Fluor 525 (5C-JF$_{525}$)

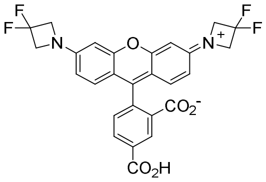

$C_{27}H_{15}F_4N_2O_5$
526.44 g/mol

The deprotected **5C-JF$_{525}$** fluorophore was synthesized from **S7** according to general procedure 2. The TFA salt of the product was obtained as dark violet solid (86.0 mg, 98%).

^{1}H NMR (500 MHz, CD_3OD): δ = 8.92 (d, J = 1.3 Hz, 1H), 8.45 (dd, J = 7.9, 1.6 Hz, 1H), 7.54 (d, J = 7.9 Hz, 1H), 7.20 (d, J = 9.1 Hz, 2H), 6.81 (d, J = 2.1 Hz, 2H), 6.78 (dd, J = 9.1, 2.2 Hz, 2H), 4.69 (t, $^3J_{HF}$ = 11.6 Hz, 8H) ppm.

^{19}F NMR (282 MHz, CD_3OD) δ = −77.04 (s), −102.55 (p, $^3J_{HF}$ = 11.7 Hz) ppm.

^{13}C NMR (126 MHz, CD_3OD): δ = 167.8, 167.3, 158.7, 157.5 (t, $^4J_{CF}$ = 4.16 Hz), 134.8, 134.5, 133.2, 132.6, 132.5, 131.5, 116.5 (t, $^1J_{CF}$ = 285 Hz), 115.6, 115.0, 97.5, 64.2 (t, $^2J_{CF}$ = 29.0 Hz) ppm.

MS (HR-ESI, pos): meas. *m/z* = 527.1224; calc. *m/z* = 527.1225 $[M+H]^+$.

_N_-(2-(2-(2-aminoethoxy)ethoxy)ethyl)-2,4-dinitroaniline (DN-NH_2)

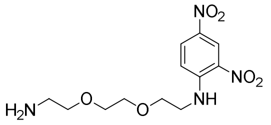

$C_{12}H_{18}N_4O_6$
314.30 g/mol

First, 2,2′-(Ethylenedioxy)bis(ethylamine) (8.58 mL, 58.8 mmol, 10.0 eq) was dissolved in DCM (40 mL). The resulting solution was cooled to 0 °C and 1-fluoro-2,4-dinitrobenzene (675 µL, 5.88 mmol, 1.00 eq) was added dropwise under stirring. The reaction mixture was allowed to warm up to room temperature and was stirred for 45 min. Next, water (200 mL) was added and the organic phase was recovered. The organic phase was extracted with 0.1 M aqueous HCl (200 mL). The pH of the aqueous phase was adjusted to pH = 12 using 1 M aqueous NaOH (~20 mL) and the aqueous layer was extracted with DCM (2 × 200 mL). The combined organic phases were washed with brine (100 mL), dried over $MgSO_4$, filtered and the solvent was removed under reduced pressure. The product was obtained as a brown oil (1.25 g, 74%). The obtained ^{1}H NMR spectrum was in accordance with the cited literature.[10]

^{1}H NMR (300 MHz, $CDCl_3$): δ = 9.14 (d, J = 2.6 Hz, 1H), 8.81 (s, 1H), 8.27 (dd, J = 9.5, 2.5 Hz, 1H), 6.94 (d, J = 9.5 Hz, 1H), 3.84 (t, J = 5.2 Hz, 2H), 3.75 – 3.64 (m, 4H), 3.60 (q, J = 5.1 Hz, 2H), 3.53 (t, J = 5.2 Hz, 2H), 2.89 (s, 2H), 1.65 (s, 2H) ppm.

5-(((2-(2-((2,4-dinitrophenyl)amino)ethoxy)ethoxy)methyl)carbamoyl) Janelia Fluor 549 (JF$_{549}$-DN)

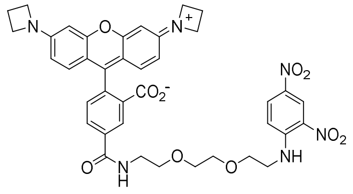

$C_{39}H_{38}N_6O_{10}$
750.77 g/mol

A *Schlenk* flask was charged with 5C-JF$_{549}$ (30.0 mg, 66.0 µmol, 1.00 eq) and TSTU (33.8 mg, 112 µmol, 1.70 eq), evacuated and backfilled with argon (3×). The solids were dissolved in anhydrous DMF (1 mL) and DIPEA (34.5 µL, 198 µmol, 5.00 eq) was added. The mixture was stirred at room temperature for 1.5 h. Next, DN-NH_2 (24.9 mg, 19.2 µmol, 1.20 eq) was dissolved in anhydrous DMF (0.5 mL) and added to the mixture. The reaction was stirred at room temperature overnight. The solvent was removed under reduced pressure and the crude product was purified by preparative reversed-phase HPLC (48 → 60% MeCN + 0.1% TFA in 40 min, R_t = 25 min) and the product was obtained as violet solid (16.9 mg, 35%).

^{1}H NMR (500 MHz, CD_3OD): δ = 8.88 (d, J = 2.7 Hz, 1H), 8.70 (d, J = 1.6 Hz, 1H), 8.21 (dd, J = 7.9, 1.7 Hz, 1H), 8.18 (dd, J = 9.6, 2.7 Hz, 1H), 7.42 (d, J = 7.9 Hz, 1H), 7.15 (d, J = 9.6 Hz, 1H), 7.01 (d, J = 9.2 Hz, 2H), 6.56 (dd, J = 9.2, 2.0 Hz, 2H), 6.50 (d, J = 2.0 Hz, 2H), 4.29 (t, J = 7.6 Hz, 8H), 3.84 (t, J = 5.2 Hz, 2H), 3.77 – 3.70 (m, 6H), 3.65 (q, J = 5.7 Hz, 4H), 2.56 (p, J = 7.6 Hz, 4H) ppm.

^{13}C NMR (126 MHz, CD_3OD): δ = 168.2, 167.3, 160.2, 158.7, 158.0, 149.8, 138.2, 137.6, 137.0, 132.8, 132.3, 132.1, 131.9, 131.4, 131.3, 131.0, 124.6, 116.1, 114.6, 113.6, 95.1, 71.7, 71.5, 70.6, 69.8, 52.9, 44.2, 41.2, 16.8 ppm.

MS (HR-ESI, pos): meas. *m/z* = 751.2713, calc. *m/z* = 751.2722 for $C_{39}H_{39}N_6O_{10}$ $[M+H]^+$.

Tetramethyl rhodamine (TMR)

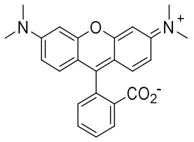

$C_{24}H_{22}N_2O_3$
386.45 g/mol

Phthalic anhydride (300 mg, 2.03 mmol, 1.00 eq) and 3-dimethylaminophenol (556 mg, 4.05 mmol, 2.00 eq) were placed inside a flask and stirred at 150 °C for 24 h. After allowing the mixture to cool to room temperature, the resulting solid was taken up in $CHCl_3$/MeOH (10:1) and purified by silica column chromatography ($CHCl_3$:MeOH = 10:1 → 5:1). The product was obtained as dark violet solid (164 mg, 21%). The measured spectrum was in accordance with literature.[11]

^{1}H NMR (300 MHz, CD_3OD): δ = 8.15 – 8.07 (m, 1H), 7.71 – 7.59 (m, 2H), 7.30 – 7.21 (m, 3H), 6.99 (dd, *J* = 9.5, 2.5 Hz, 2H), 6.89 (d, *J* = 2.5 Hz, 2H), 3.26 (s, 12H) ppm.

MS (HR-ESI, pos): meas. *m/z* = 387.1714, calc. *m/z* = 387.1703 for $C_{24}H_{23}N_2O_3$ $[M+H]^+$.

General procedure for the amide coupling reaction of 3-carboxy group of rhodamines (GP3)

The following procedure is exemplary for the amide coupling of the 3-carboxy functionality of rhodamine derivatives with the corresponding amines to obtain the respective cyclic lactams.

A reaction vessel was charged with the rhodamine derivative (1.00 eq), EDC hydrochloride (8.00 eq), DMAP (8.00 eq) and corresponding amine (1.20 eq), evacuated and backfilled with argon (3×). The solids were dissolved in anhydrous DCM (25 mM solution) and stirred at 60 °C overnight. The solvent was removed under reduced pressure and the crude product was purified by silica column chromatography.

3-(Cyanocarbamoyl) tetramethyl rhodamine (1)

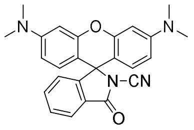

$C_{25}H_{22}N_4O_2$
410.48 g/mol

Rhodamine **1** was synthesized from TMR and cyanamide according to general procedure 3. The obtained crude mixture was purified by silica column chromatography ($CHCl_3$:MeOH = 10:0 → 50:1) and the product was obtained as violet solid (42.7 mg, 80%).

^{1}H NMR (500 MHz, $CDCl_3$): δ = 8.00 (d, *J* = 7.6 Hz, 1H), 7.63 (m, 1H), 7.58 - 7.54 (m, 1H), 7.14 (d, *J* = 7.7 Hz, 1H), 6.58 (d, *J* = 8.9 Hz, 2H), 6.47 (d, *J* = 2.5 Hz, 2H), 6.41 (dd, *J* = 8.9, 2.5 Hz, 2H), 2.99 (s, 12H) ppm.

^{13}C NMR (126 MHz, $CDCl_3$): δ = 166.6, 153.1, 152.6, 152.2, 135.7, 129.5, 128.3, 126.7, 124.9, 124.5, 109.3, 106.9, 104.3, 99.0, 68.8, 40.3 ppm.

MS (HR-ESI, pos): meas. *m/z* = 411.1814 calc. *m/z* = 411.1816 $[M+H]^+$.

3-((Methylsulfonyl)carbamoyl) tetramethyl rhodamine (2)

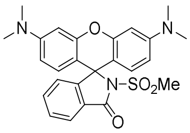

$C_{25}H_{25}N_3O_4S$
463.55 g/mol

Rhodamine **2** was synthesized from TMR and methanesulfonamide according to general procedure 3. The obtained crude mixture was purified by silica column chromatography ($CHCl_3$:MeOH = 10:0 → 50:1) and the product was obtained as violet solid (43.0 mg, 72%).

^{1}H NMR (500 MHz, $CDCl_3$): δ = 7.98 (d, J = 7.6 Hz, 1H), 7.58 (m, 1H), 7.52 (m, 1H), 7.08 (d, J = 7.7 Hz, 1H), 6.58 (d, J = 8.8 Hz, 2H), 6.47 (d, J = 2.5 Hz, 2H), 6.36 (dd, J = 8.8, 2.5 Hz, 2H), 2.97 (s, 12H), 2.93 (s, 3H) ppm.

^{13}C NMR (126 MHz, $CDCl_3$): δ = 167.5, 153.7, 153.1, 151.6, 135.3, 129.1, 128.2, 128.1, 124.7, 124.1, 108.5, 106.8, 99.1, 69.2, 42.1, 40.4 ppm.

MS (HR-ESI, pos): meas. m/z = 486.1417 calc. m/z = 486.1458 for $C_{25}H_{25}N_3NaO_4S$ $[M+Na]^+$.

3-((*N,N*-Dimethylsulfamoyl)carbamoyl) tetramethyl rhodamine / SpyRho (4)

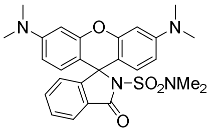

$C_{26}H_{28}N_4O_4S$
492.59 g/mol

SpyRho (**4**) was synthesized from TMR and *N*,*N*-dimethylsulfamide according to general procedure 3. The obtained crude mixture was purified by silica column chromatography ($CHCl_3$:MeOH = 10:0 → 50:1) and the product was obtained as violet solid (53.2 mg, 83%).

^{1}H NMR (500 MHz, $CDCl_3$): δ = 7.93 (d, J = 7.5 Hz, 1H), 7.57 – 7.48 (m, 2H), 7.06 (d, J = 7.6 Hz, 1H), 6.58 (d, J = 8.8 Hz, 2H), 6.48 (s, 2H), 6.35 (d, J = 8.4 Hz, 2H), 2.96 (s, 12H), 2.72 (s, 6H) ppm.

^{13}C NMR (126 MHz, $CDCl_3$): δ = 167.5, 154.0, 153.4, 151.5, 134.7, 128.8, 128.8, 128.4, 124.9, 123.7, 108.2, 107.6, 99.0, 68.9, 40.4, 38.0 ppm.

MS (HR-ESI, pos): meas. m/z = 493.1915; calc. m/z = 493.1904 for $C_{26}H_{29}N_4O_4S$ $[M+H]^+$.

3-((2,2,2-Trifluoroethyl)carbamoyl) tetramethyl rhodamine (5)

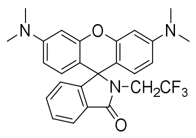

$C_{26}H_{24}F_3N_3O_2$
467.49 g/mol

Rhodamine **5** was synthesized from TMR and 2,2,2-trifluorethylamine according to general procedure 3. The obtained crude mixture was purified by silica column chromatography ($CHCl_3$:MeOH = 10:0 → 100:1) and the product was obtained as violet solid (36.5 mg, 60%).

^{1}H NMR (500 MHz, $CDCl_3$): δ = 7.95 (dd, J = 5.8, 2.7 Hz, 1H), 7.52 – 7.43 (m, 2H), 7.06 (dd, J = 5.7, 2.4 Hz, 1H), 6.49 – 6.41 (m, 4H), 6.35 (dd, J = 8.8, 2.5 Hz, 2H), 3.72 (q, $^3J_{HF}$ = 9.3 Hz, 2H), 2.97 (s, 12H) ppm.

^{19}F NMR (282 MHz, $CDCl_3$) δ = −68.4 (t, $^3J_{HF}$ = 9.4 Hz) ppm.

^{13}C NMR (126 MHz, $CDCl_3$): δ = 169.4, 154.1, 153.1, 151.6, 133.5, 129.5, 128.9, 128.5, 124.2, 123.8 (q, J_{CF} = 281 Hz)*, 123.5, 123.4, 109.0, 105.8, 98.7, 98.7, 65.6, 41.8*, 40.4 ppm.

Note: Highlighted peaks (*) were only detected in HSQC or HMBC experiments.

MS (HR-ESI, pos): meas. *m/z* = 468.1894; calc. *m/z* = 468.1893 for $C_{26}H_{25}F_3N_3O_2$ $[M+H]^+$.

3-(Sulfamoylcarbamoyl) tetramethyl rhodamine (3)

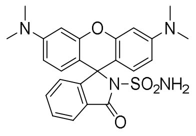

$C_{24}H_{24}N_4O_4S$
464.549 g/mol

A heat-dried *Schlenk* flask was charged with TMR (55.0 mg, 142 µmol, 1.00 eq), evacuated and backfilled with argon (3×). The solid was dissolved in anhydrous DCM (2 mL) and $POCl_3$ (266 µL, 436 mg, 2.85 mmol, 20.0 eq) was added dropwise. The solution was stirred at 60 °C for 3 h and the solvent was removed under reduced pressure to give the crude carboxylic acid chloride as a dark violet solid.

Next, sulfamide (136.8 mg, 1.42 mmol, 10.0 eq) and anhydrous DIPEA (372 µL, 276 mg, 2.13 mmol, 15.0 eq) were dissolved in anhydrous MeCN (4 mL) and added to the crude acyl chloride. The mixture was stirred at 70 °C for 1.5 h. The solvent was evaporated and the residue was taken up in chloroform (30 mL). The organic phase was washed with water (10 mL), dried over magnesium sulfate and filtered. After evaporation of the solvent, the crude product was purified by silica column chromatography ($CHCl_3$:MeOH = 10:0 → 50:1). The product was obtained as violet solid (23.1 mg, 35%)

^{1}H NMR (500 MHz, $(CD_3)_2SO$): δ = 7.89 (d, J = 7.3 Hz, 1H), 7.62 – 7.58 (m, 1H), 7.56 (s, 2H), 7.55 – 7.52 (m, 1H), 6.92 (d, J = 7.6 Hz, 1H), 6.51 – 6.47 (m, 2H), 6.40 (m, 4H), 2.91 (s, 12H) ppm.

^{13}C NMR (126 MHz, $(CD_3)_2SO$): δ = 166.2, 153.8, 151.9, 150.9, 134.8, 128.0, 127.2, 124.1, 123.1, 109.5, 108.5, 107.1, 98.3, 67.4, 39.9 ppm.

MS (HR-ESI, pos): meas. m/z = 487.1437; calc. m/z = 487.1410 for $C_{24}H_{24}N_4NaO_4S$ $[M+Na]^+$.

Fluorescein ditriflate (S10)

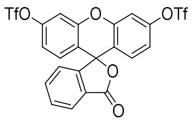

$C_{22}H_{10}F_6O_9S_2$
596.42 g/mol

A reaction vessel was charged with fluorescein (100 mg, 299 µmol, 1.00 eq), evacuated and backfilled with argon (3×). The starting material was suspended in anhydrous DCM (1.5 mL) and cooled to 0 °C. Next, Pyridine (193 µL, 189 mg, 2.39 mmol, 8.00 eq) was added followed by the dropwise addition of Tf_2O (201 µL, 338 mg, 1.20 mmol, 4.00 eq). The solution was allowed to warm up to room temperature and was stirred for 6 h. The reaction was quenched with water and the obtained mixture was diluted with DCM (30 mL). The reaction mixture was washed with saturated $CuSO_4$ solution (25 mL) and brine (25 mL). The combined organic layers were dried over magnesium sulfate, filtered, and the solvent was removed under reduced pressure. The crude product was purified by silica column chromatography (cyclo-hexane:EtOAc = 10:0 → 3:1). The product was obtained as colorless solid (137 mg, 70%). The recorded spectrum was in accordance with literature.[12]

^{1}H NMR (300 MHz, $CDCl_3$): δ = 8.13 – 8.05 (m, 1H), 7.72 (m, 2H), 7.30 (d, J = 2.3 Hz, 2H), 7.22 – 7.16 (m, 1H), 7.08 – 6.92 (m, 4H) ppm.

MS (HR-ESI, pos): meas. m/z = 618.9574, calc. m/z = 618.9563 for $C_{22}H_{10}F_6NaO_9S_2$ $[M+Na]^+$.

Janelia Fluor 549 (JF_{549})

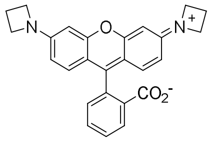

$C_{26}H_{22}N_2O_3$
410.47 g/mol

A reaction vessel was charged with fluorescein ditriflate (137 mg, 230 µmol, 1.00 eq), Cs_2CO_3 (210 mg, 643 µmol, 2.80 eq), $Pd_2(dba)_3$ (42.1 mg, 45.9 µmol, 0.20 eq) and 2-dicyclohexyl-phosphino-2′,4′,6′-triisopropylbiphenyl (XPhos; 32.9 mg, 68.9 µmol, 0.30 eq), sealed, evacuated and backfilled with argon (3×). The solids were suspended in dry dioxane (2 mL). Azetidine (31.5 mg, 551 µmol, 2.40 eq) was added and the mixture was stirred at 100 °C overnight. The mixture was diluted with MeOH and the solvent was evaporated under reduced pressure. The crude product was purified by silica column chromatography ($CHCl_3$:2 M NH_3 in MeOH = 10:0 → 9:1). The product was obtained as violet solid (19.0 mg, 20%). The recorded spectrum was in accordance with literature.[13]

^{1}H NMR (300 MHz, $CDCl_3$): δ = 8.00 (d, J = 7.6 Hz, 1H), 7.68 – 7.53 (m, 2H), 7.17 (d, J = 7.9 Hz, 1H), 6.57 (d, J = 8.6 Hz, 2H), 6.21 (d, J = 2.2 Hz, 2H), 6.09 (dd, J = 8.6, 2.3 Hz, 2H), 3.92 (t, J = 7.3 Hz, 8H), 2.38 (p, J = 7.5 Hz, 4H) ppm.

MS (HR-ESI, pos): meas. m/z = 411.1718, calc. m/z = 411.1703 for $C_{26}H_{23}N_2O_3$ $[M+H]^+$.

3-((*N,N*-Dimethylsulfamoyl)carbamoyl) Janelia Fluor 549 (6)

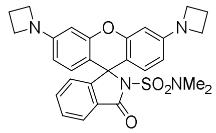

$C_{28}H_{28}N_4O_4S$
516.62 g/mol

A reaction vessel was charged with JF_{549} (18.0 mg, 43.9 µmol, 1.00 eq), EDC hydrochloride (67.3 mg, 351 µmol, 8.00 eq), DMAP (42.9 mg, 351 µmol, 8.00 eq) and *N*,*N*-dimethylsulfamide (6.53 mg, 52.6 µmol, 1.20 eq) evacuated and backfilled with argon (3×). The solids were dissolved in anhydrous DCM (2 mL) and stirred at 60 °C overnight. The solvent was removed under reduced pressure and the crude product was purified by silica column chromatography ($CHCl_3$:MeOH = 10:0 → 50:1). The product was obtained as violet solid (18.9 mg, 83%).

^{1}H NMR (500 MHz, $CDCl_3$): δ = 7.92 (d, J = 7.4 Hz, 1H), 7.55 (m, 1H), 7.49 (m, 1H), 7.05 (d, J = 7.5 Hz, 1H), 6.53 (d, J = 8.5 Hz, 2H), 6.23 – 6.15 (m, 2H), 6.07 – 5.97 (m, 2H), 3.89 (t, J = 7.2 Hz, 8H), 2.71 (s, 6H), 2.36 (p, J = 7.2 Hz, 4H) ppm.

^{13}C NMR (126 MHz, $CDCl_3$): δ = 167.4, 154.1, 153.2, 153.0, 134.8, 128.8, 128.8, 128.4, 124.9, 123.7, 108.2, 107.1, 97.9, 69.0, 52.2, 38.0, 16.8 ppm.

MS (HR-ESI, pos): meas. m/z = 517.1900; calc. m/z = 517.1904 for $C_{28}H_{29}N_4O_4S$ $[M+H]^+$.

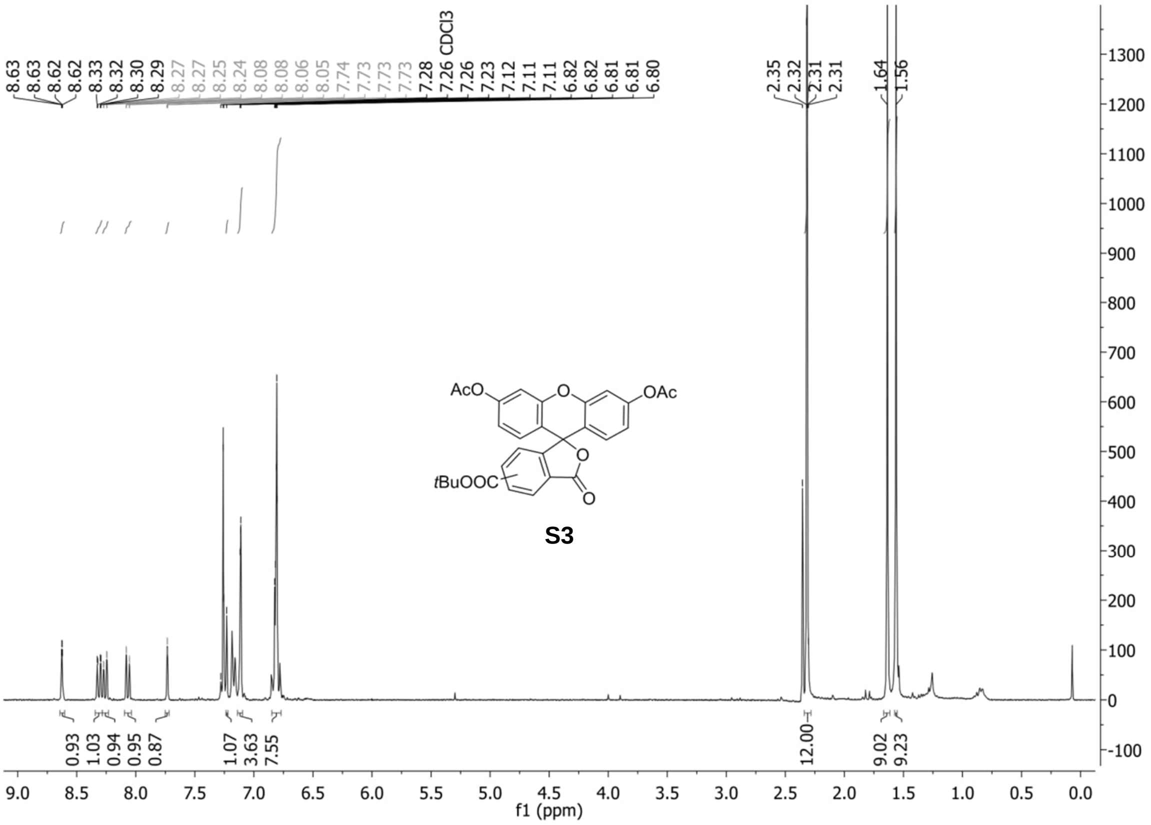
8.63
8.63
8.62
8.62
8.33
8.32
8.30
8.29
8.27
8.27
8.25
8.24
8.08
8.08
8.06
8.05
7.74
7.73
7.73
7.73
7.28
7.26 CDCl3
7.26
7.23
7.12
7.11
7.11
6.82
6.82
6.81
6.81
6.80
2.35
2.32
2.31
2.31
1.64
1.56
AcO
OAc
tBuOOC
S3
0.93
1.03
0.94
0.95
0.87
1.07
3.63
7.55
12.00
9.02
9.23
f1 (ppm)

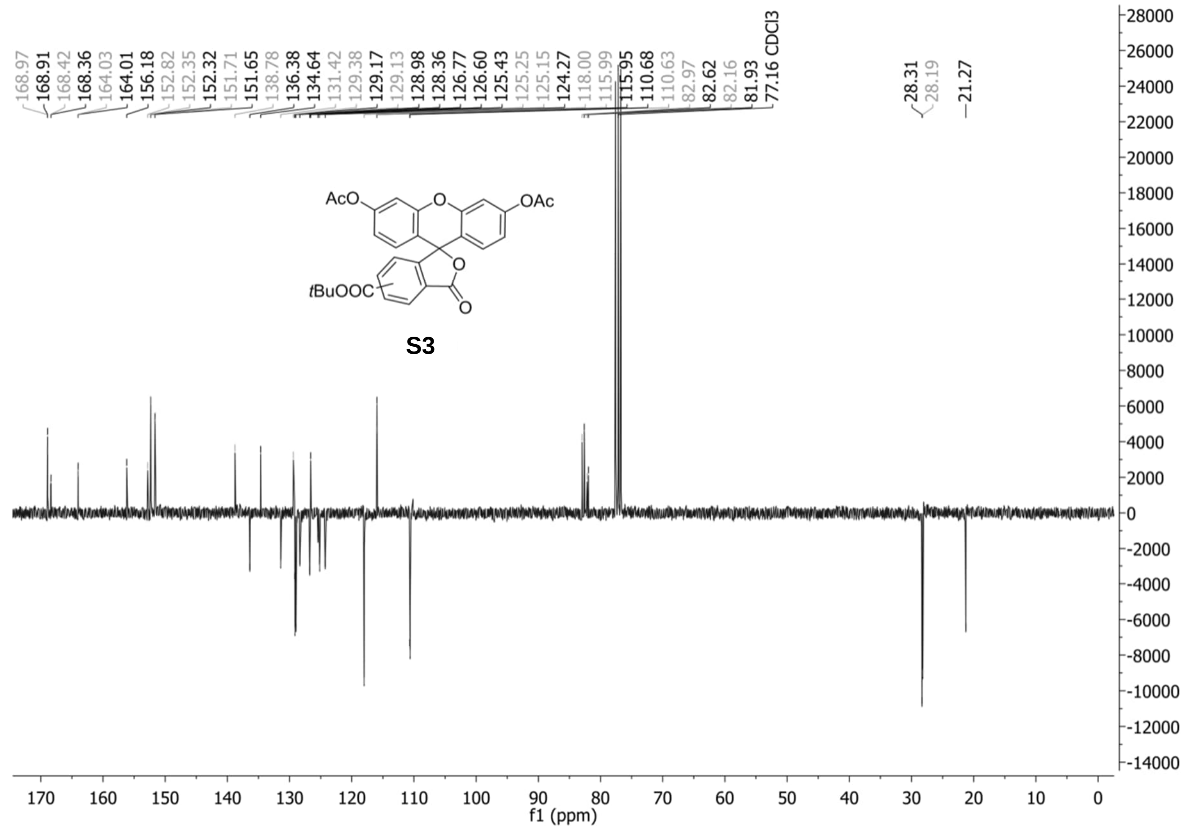
168.97
168.91
168.42
168.36
164.03
164.01
156.18
152.82
152.35
152.32
151.71
151.65
138.78
136.38
134.64
131.42
129.38
129.17
129.13
128.98
128.36
126.77
126.60
125.43
125.25
125.15
124.27
118.00
115.99
115.95
110.68
110.63
82.97
82.62
82.16
81.93
77.16 CDCl3
28.31
28.19
21.27
AcO
OAc
tBuOOC
S3
f1 (ppm)

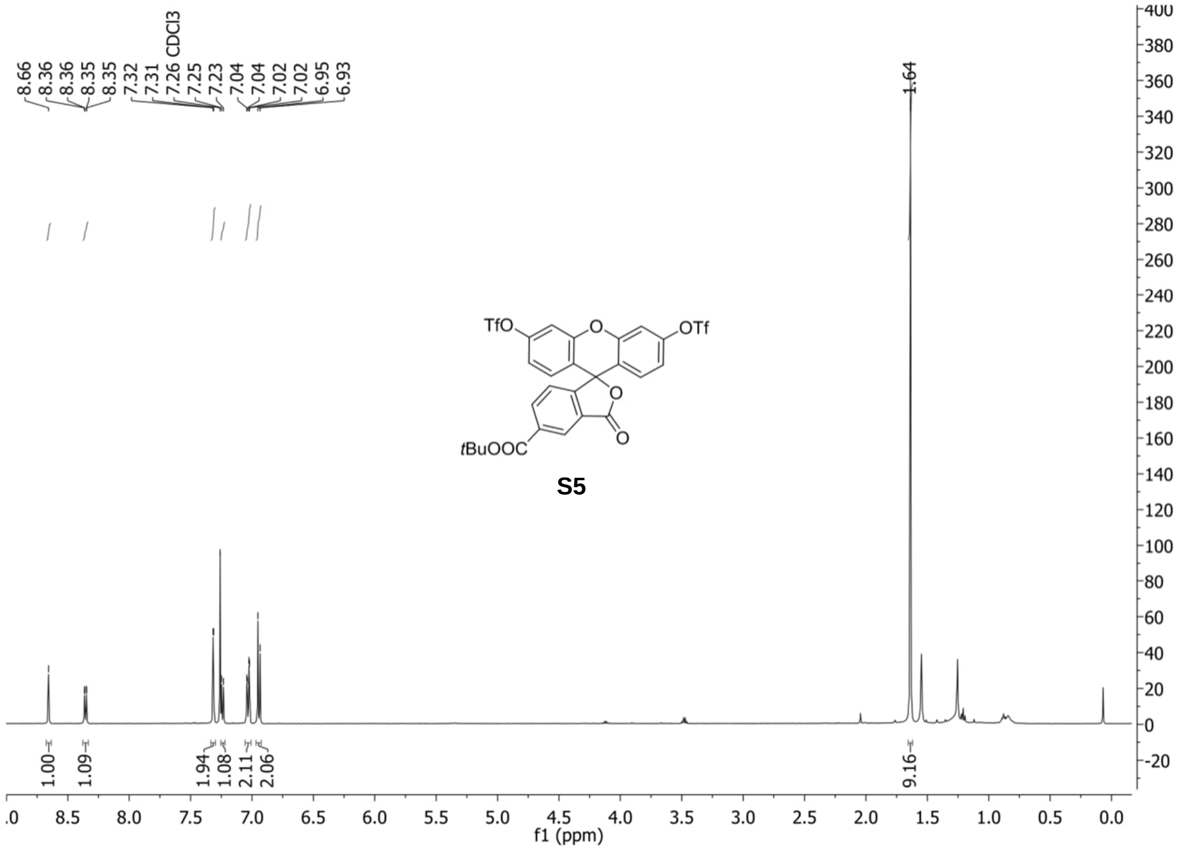
8.66
8.36
8.36
8.35
8.35
7.32
7.31
7.26 CDCl3
7.25
7.23
7.04
7.04
7.02
7.02
6.95
6.93
1.64
TfO
OTf
O
O
O
tBuOOC
S5
1.00
1.09
1.94
1.08
2.11
2.06
9.16
f1 (ppm)

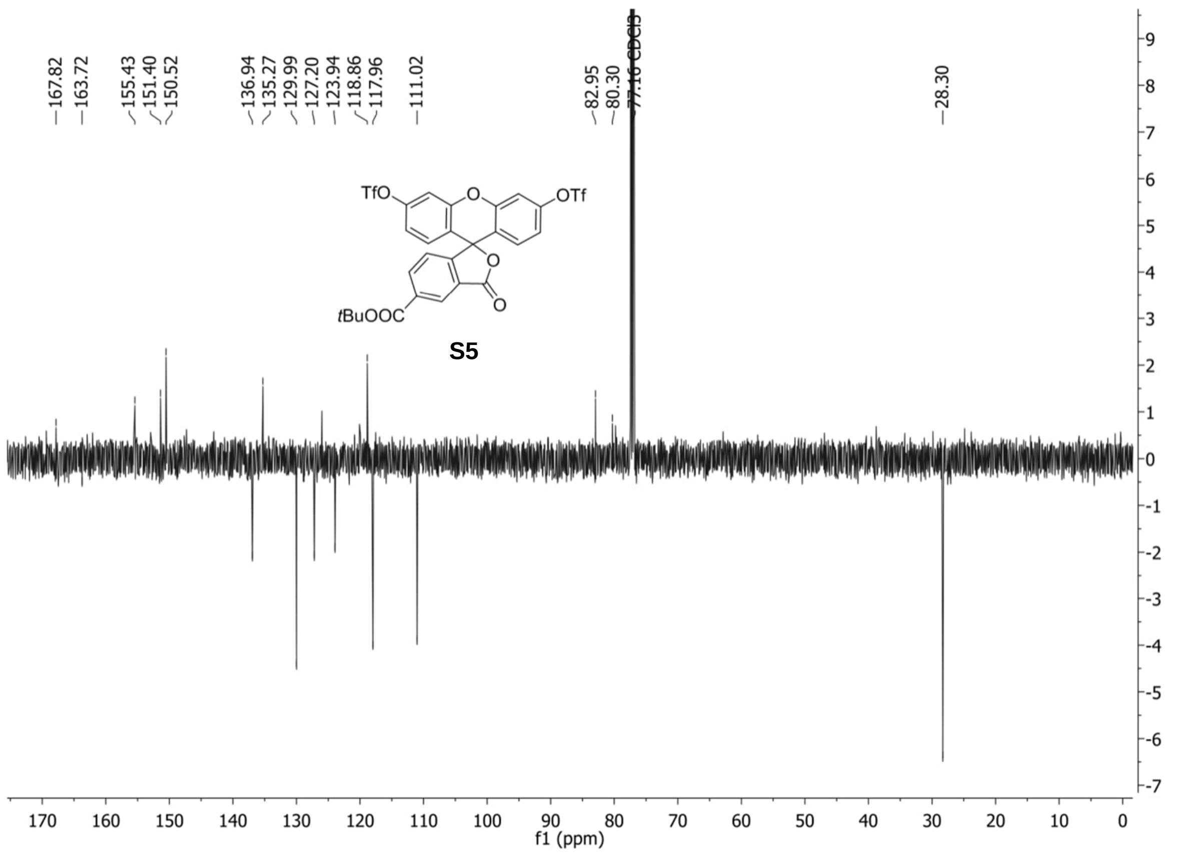
167.82
163.72
155.43
151.40
150.52
136.94
135.27
129.99
127.20
123.94
118.86
117.96
111.02
82.95
80.30
77.16 CDCl3
28.30
TfO
OTf
O
O
O
tBuOOC
S5
f1 (ppm)

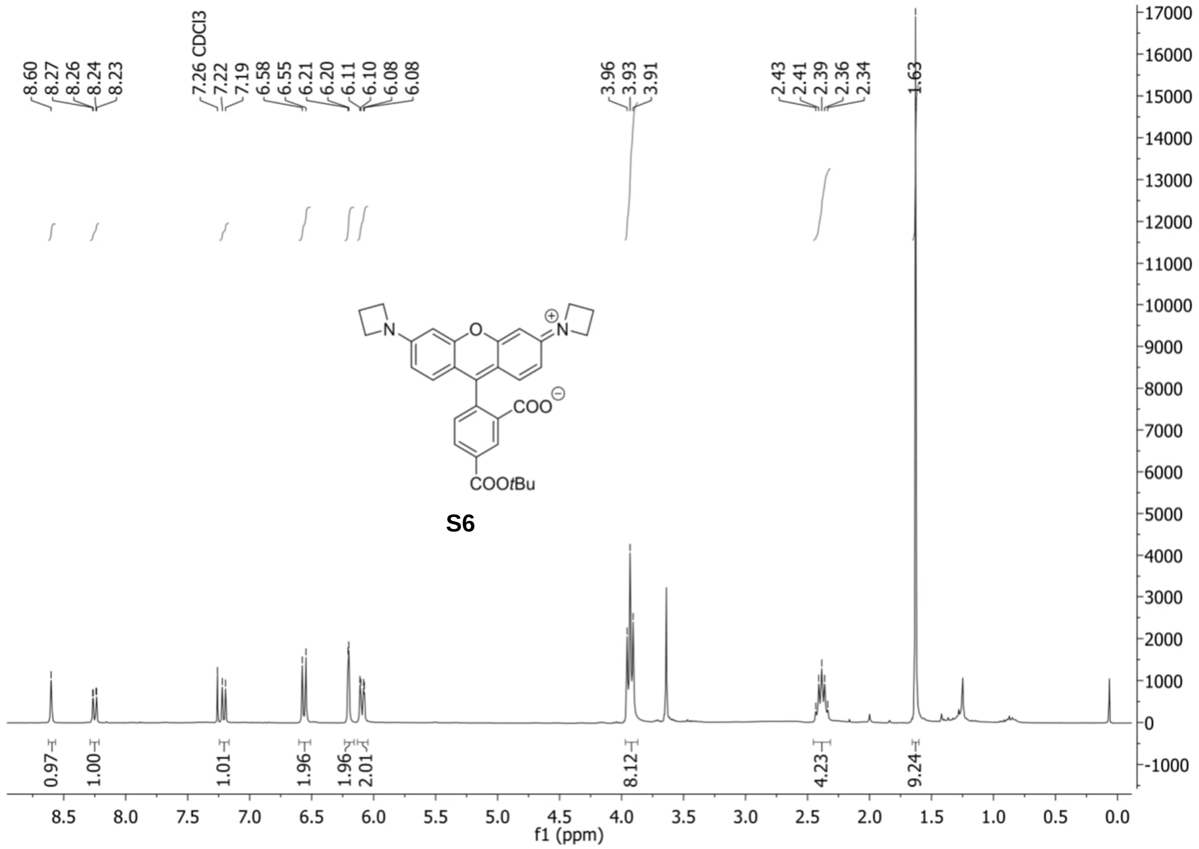
8.60
8.27
8.26
8.24
8.23
7.26 CDCl3
7.22
7.19
6.58
6.55
6.21
6.20
6.11
6.10
6.08
6.08
3.96
3.93
3.91
2.43
2.41
2.39
2.36
2.34
1.63
COO⊖
COOtBu
S6
0.97
1.00
1.01
1.96
1.96
2.01
8.12
4.23
9.24
f1 (ppm)

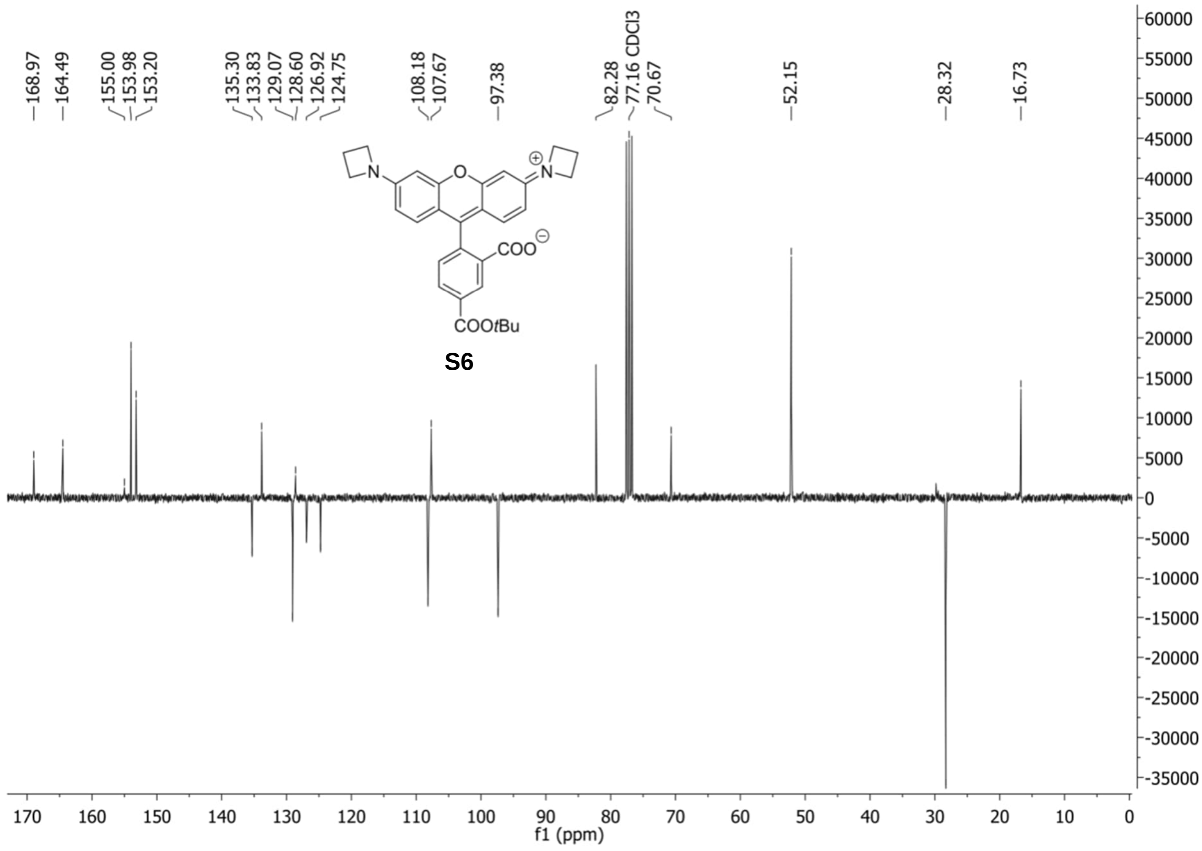
168.97
164.49
155.00
153.98
153.20
135.30
133.83
129.07
128.60
126.92
124.75
108.18
107.67
97.38
82.28
77.16 CDCl3
70.67
52.15
28.32
16.73
COO⊖
COOtBu
S6
f1 (ppm)

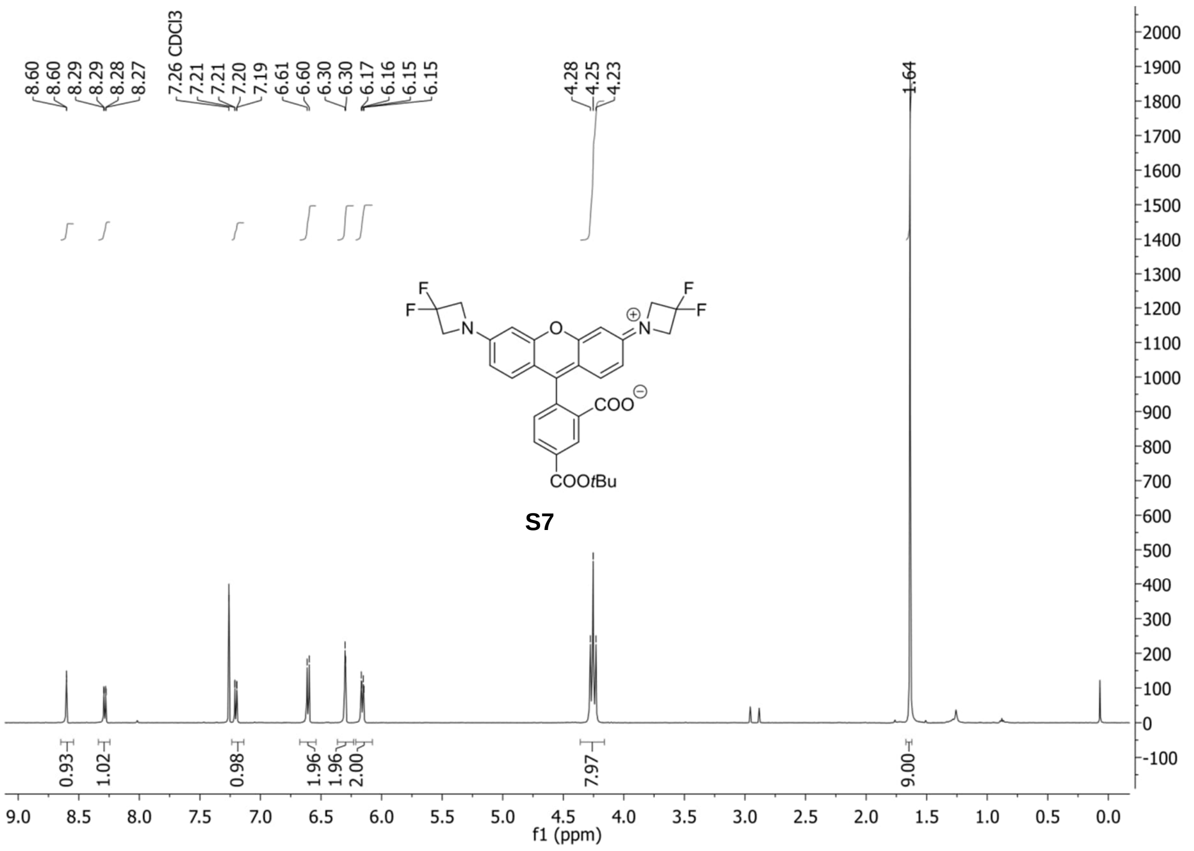
8.60
8.60
8.29
8.29
8.28
8.27
7.26 CDCl3
7.21
7.21
7.20
7.19
6.61
6.60
6.30
6.30
6.17
6.16
6.15
6.15
4.28
4.25
4.23
1.64
COO
COOtBu
S7
0.93
1.02
0.98
1.96
1.96
2.00
7.97
9.00
f1 (ppm)

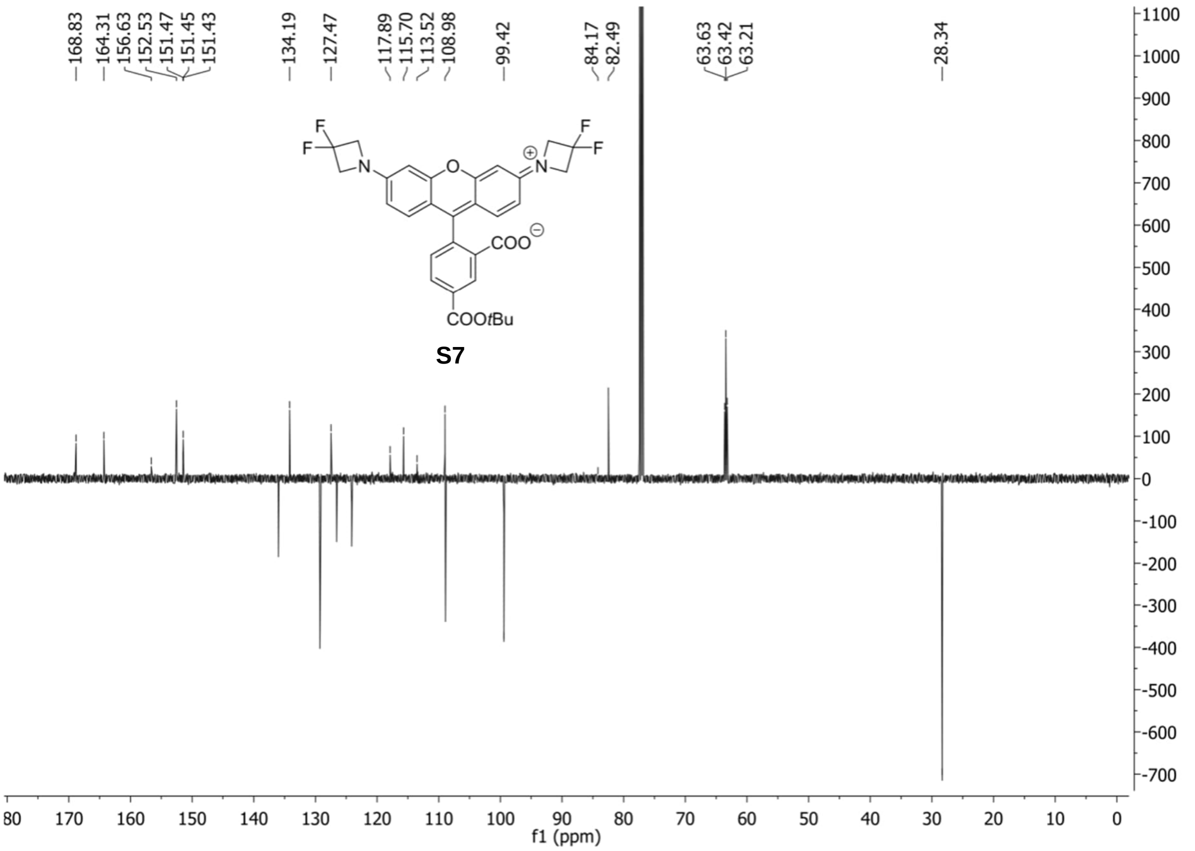
168.83
164.31
156.63
152.53
151.47
151.45
151.43
134.19
127.47
117.89
115.70
113.52
108.98
99.42
84.17
82.49
63.63
63.42
63.21
28.34
COO
COOtBu
S7
f1 (ppm)

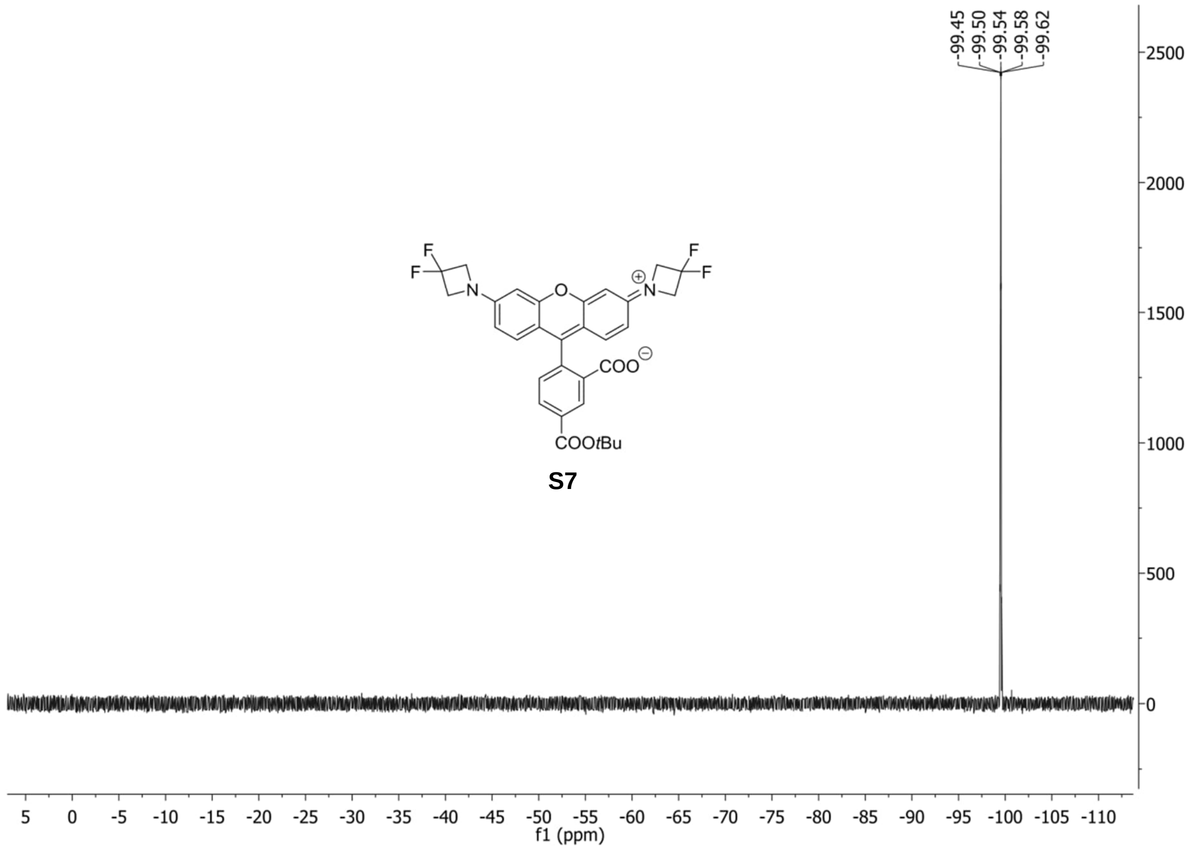
-99.45
-99.50
-99.54
-99.58
-99.62
F
F
N
O
N
F
F
COO
COOtBu
S7
2500
2000
1500
1000
500
0
5 0 -5 -10 -15 -20 -25 -30 -35 -40 -45 -50 -55 -60 -65 -70 -75 -80 -85 -90 -95 -100 -105 -110
f1 (ppm)

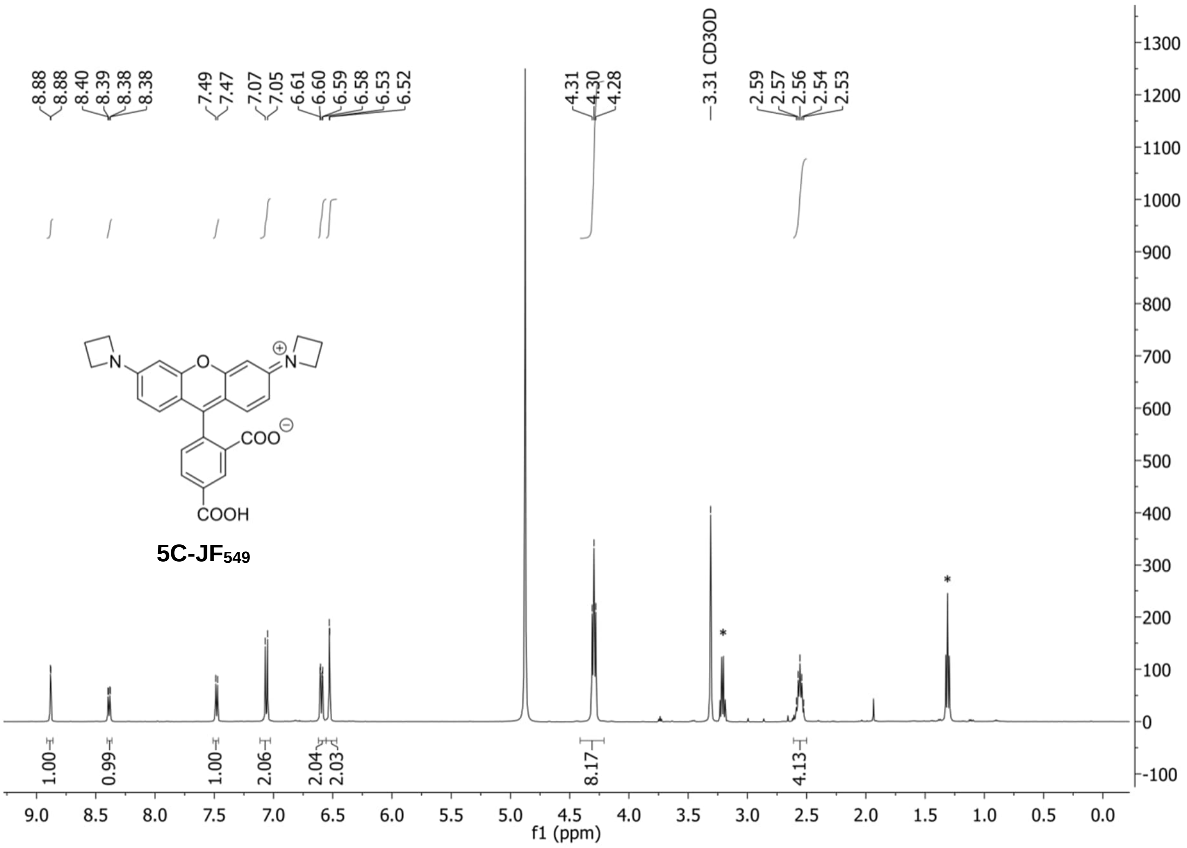

Signals indicated with * correspond to Et_3N.

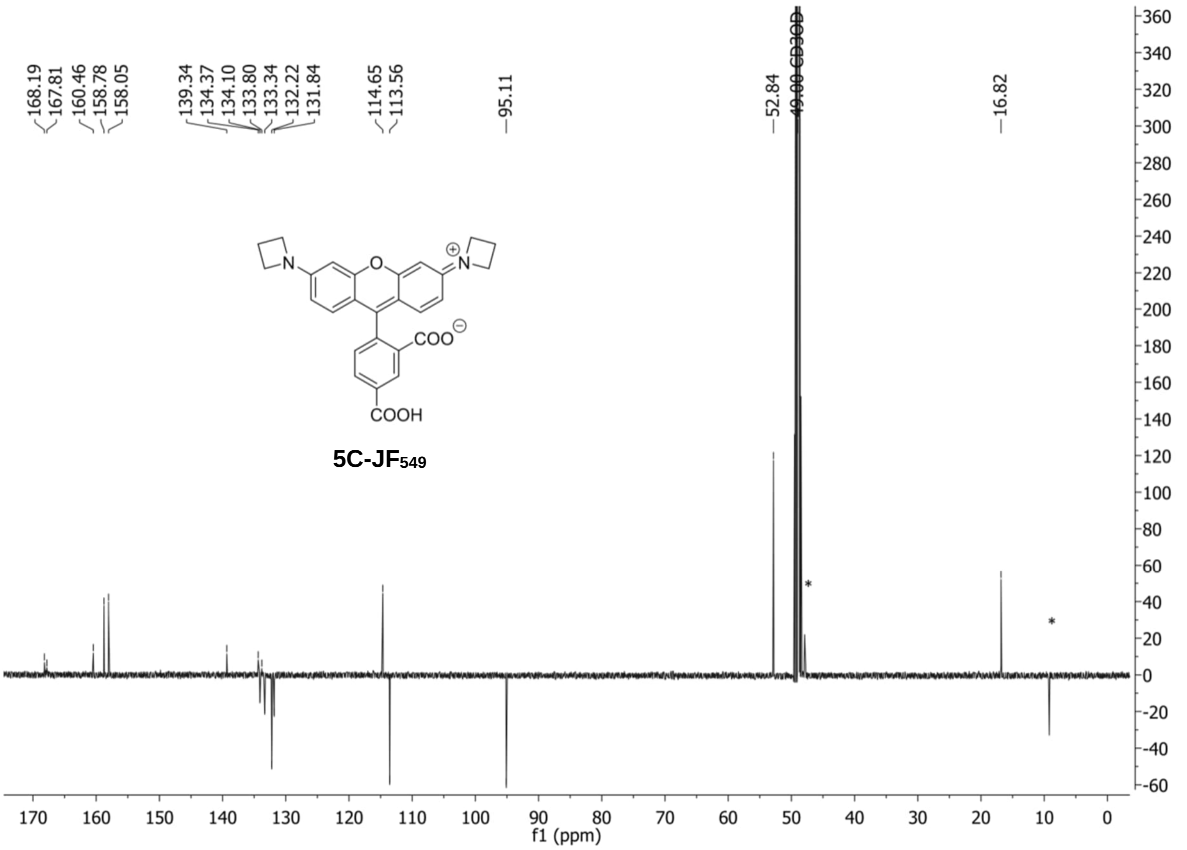

Signals indicated with * correspond to Et_3N.

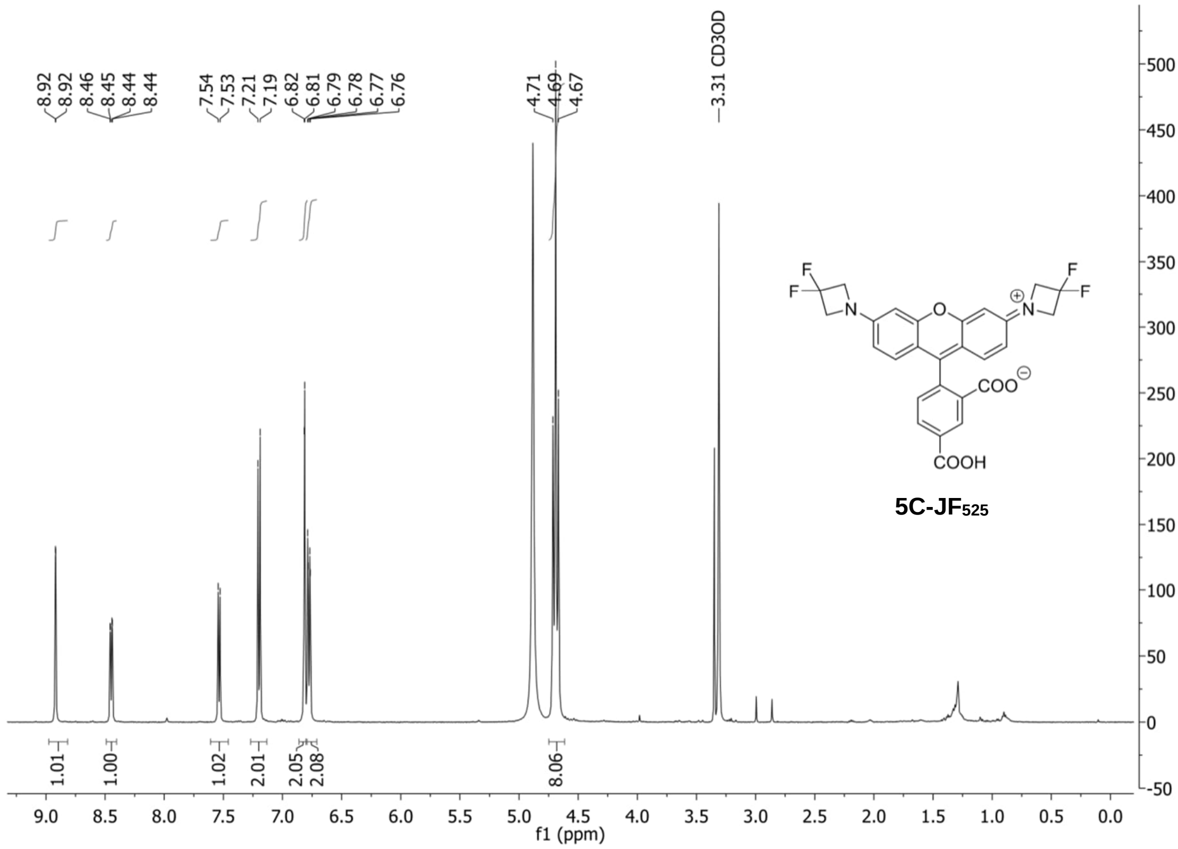
8.92
8.92
8.46
8.45
8.44
8.44
7.54
7.53
7.21
7.19
6.82
6.81
6.79
6.78
6.77
6.76
4.71
4.69
4.67
3.31 CD3OD
1.01
1.00
1.02
2.01
2.05
2.08
8.06
5C-JF525
COO
COOH
f1 (ppm)

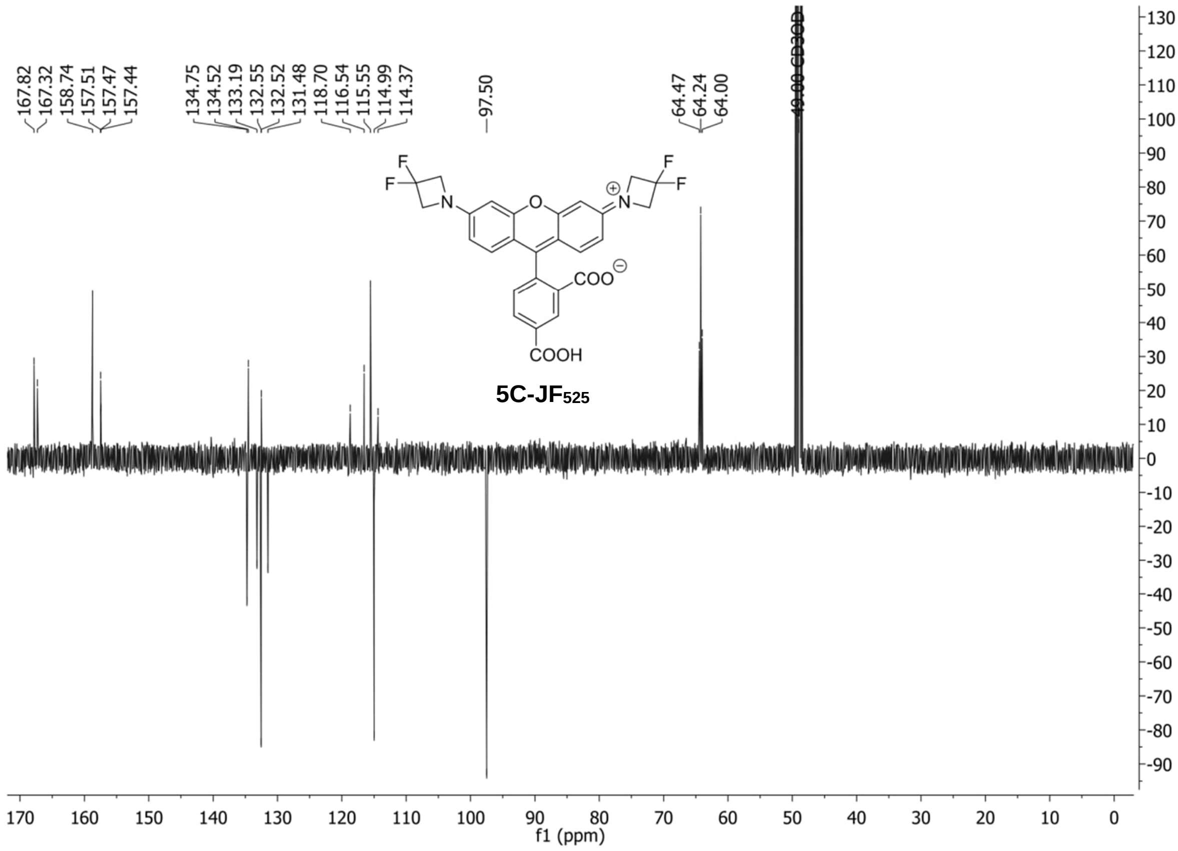
167.82
167.32
158.74
157.51
157.47
157.44
134.75
134.52
133.19
132.55
132.52
131.48
118.70
116.54
115.55
114.99
114.37
97.50
64.47
64.24
64.00
49.00 CD3OD
5C-JF525
COO
COOH
f1 (ppm)

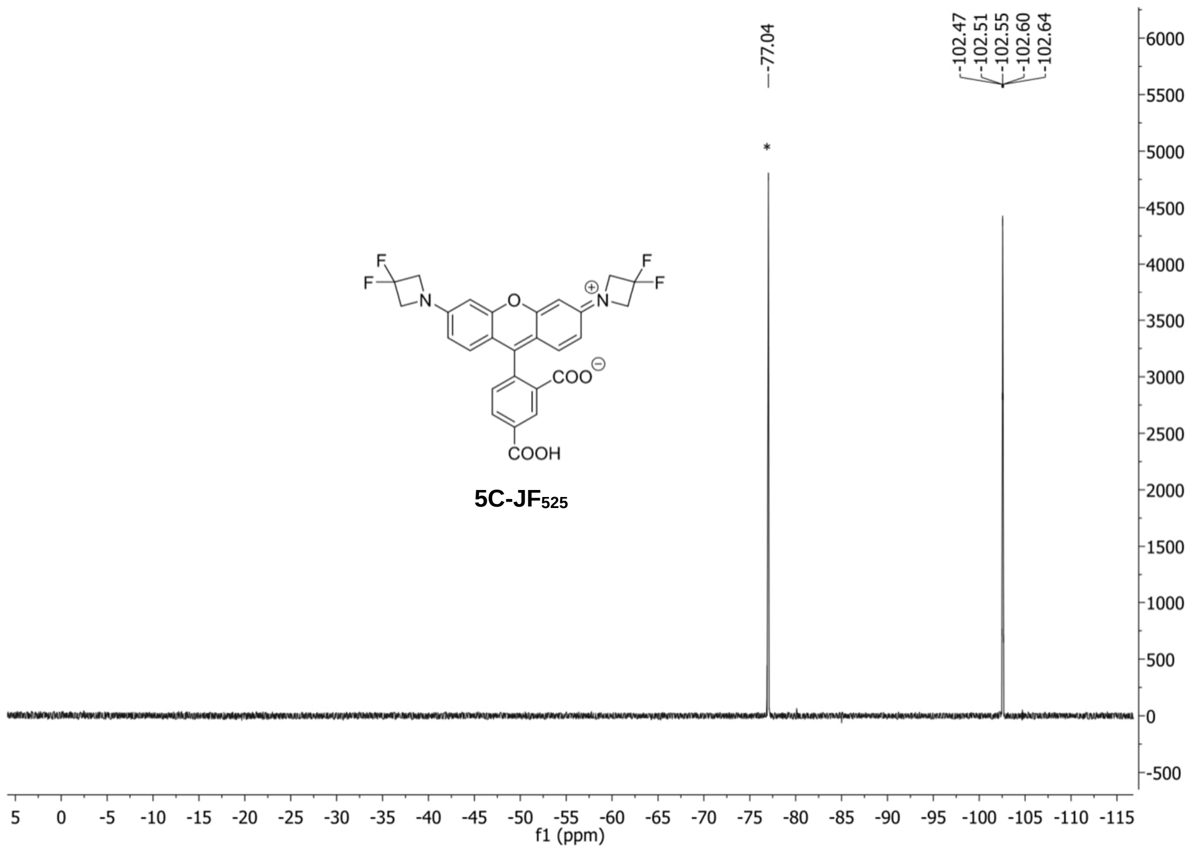

Signal indicated with * corresponds to TFA.

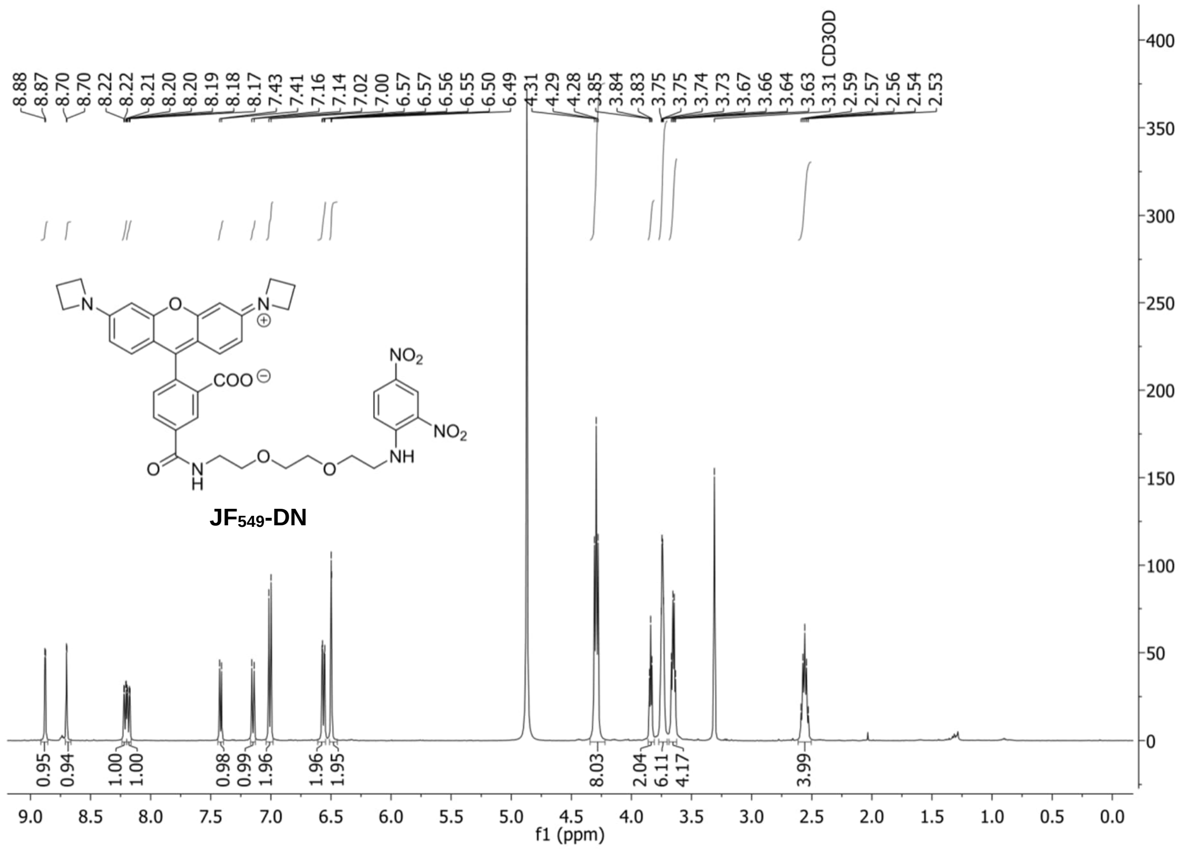
8.88 8.87 8.70 8.70 8.22 8.22 8.21 8.20 8.20 8.19 8.18 8.17 7.43 7.41 7.16 7.14 7.02 7.00 6.57 6.57 6.56 6.55 6.50 6.49 4.31 4.29 4.28 3.85 3.84 3.83 3.75 3.75 3.74 3.73 3.67 3.66 3.64 3.63 3.31 CD3OD 2.59 2.57 2.56 2.54 2.53
0.95 0.94 1.00 1.00 0.98 0.99 1.96 1.96 1.95 8.03 2.04 6.11 4.17 3.99
COO⊖
NO2
NO2
NH
O
H
N
JF549-DN
f1 (ppm)

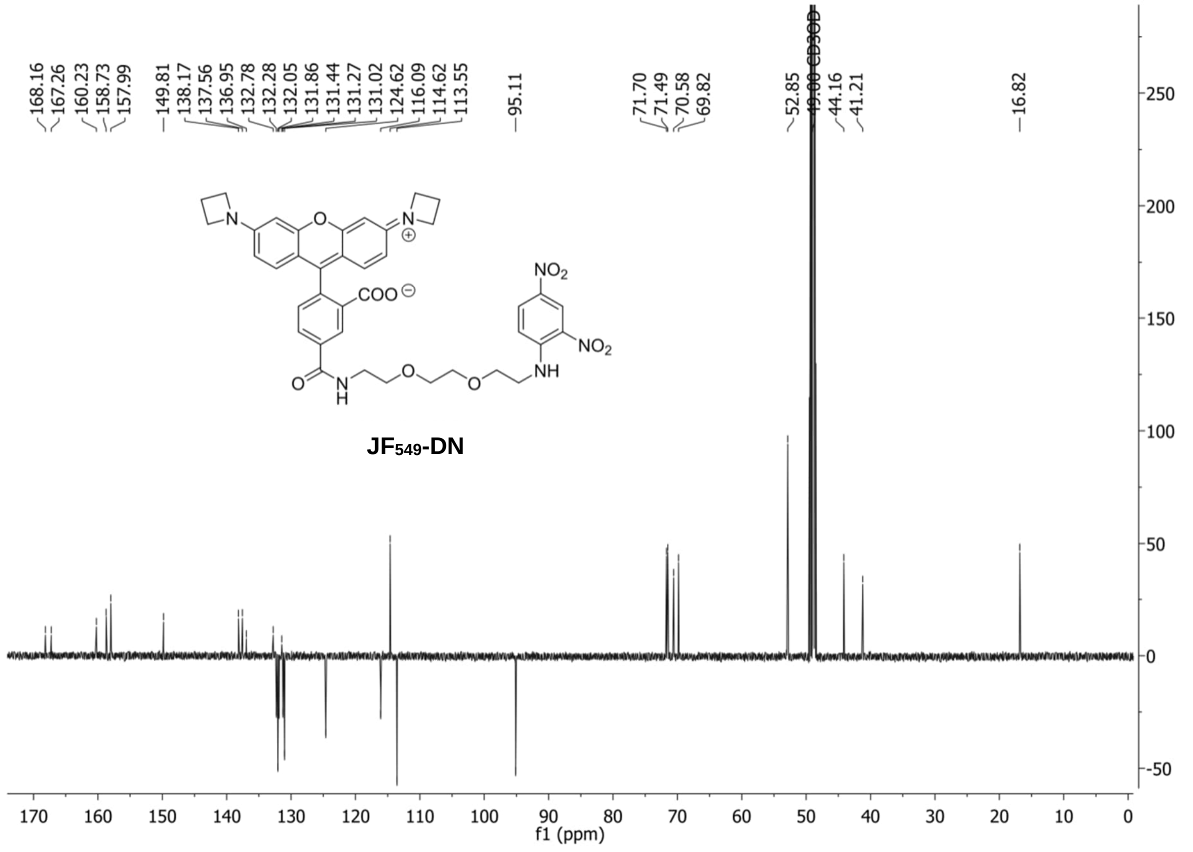
168.16 167.26 160.23 158.73 157.99 149.81 138.17 137.56 136.95 132.78 132.28 132.05 131.86 131.44 131.27 131.02 124.62 116.09 114.62 113.55 95.11 71.70 71.49 70.58 69.82 52.85 49.00 CD3OD 44.16 41.21 16.82
COO⊖
NO2
NO2
NH
JF549-DN
f1 (ppm)

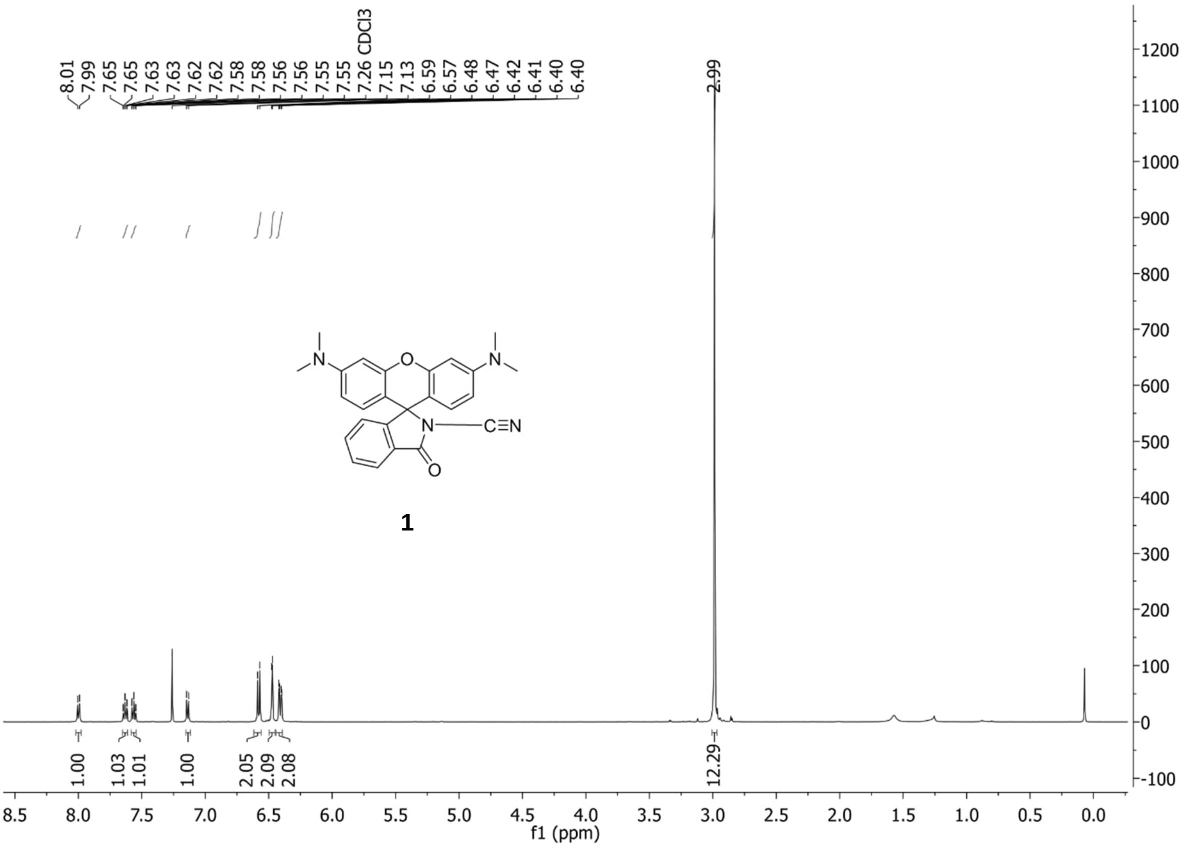
8.01
7.99
7.65
7.65
7.63
7.63
7.62
7.62
7.58
7.58
7.56
7.56
7.55
7.55
7.26 CDCl3
7.15
7.13
6.59
6.57
6.48
6.47
6.42
6.41
6.40
6.40
2.99
N
O
N
N
C≡N
O
1
1.00
1.03
1.01
1.00
2.05
2.09
2.08
12.29
f1 (ppm)

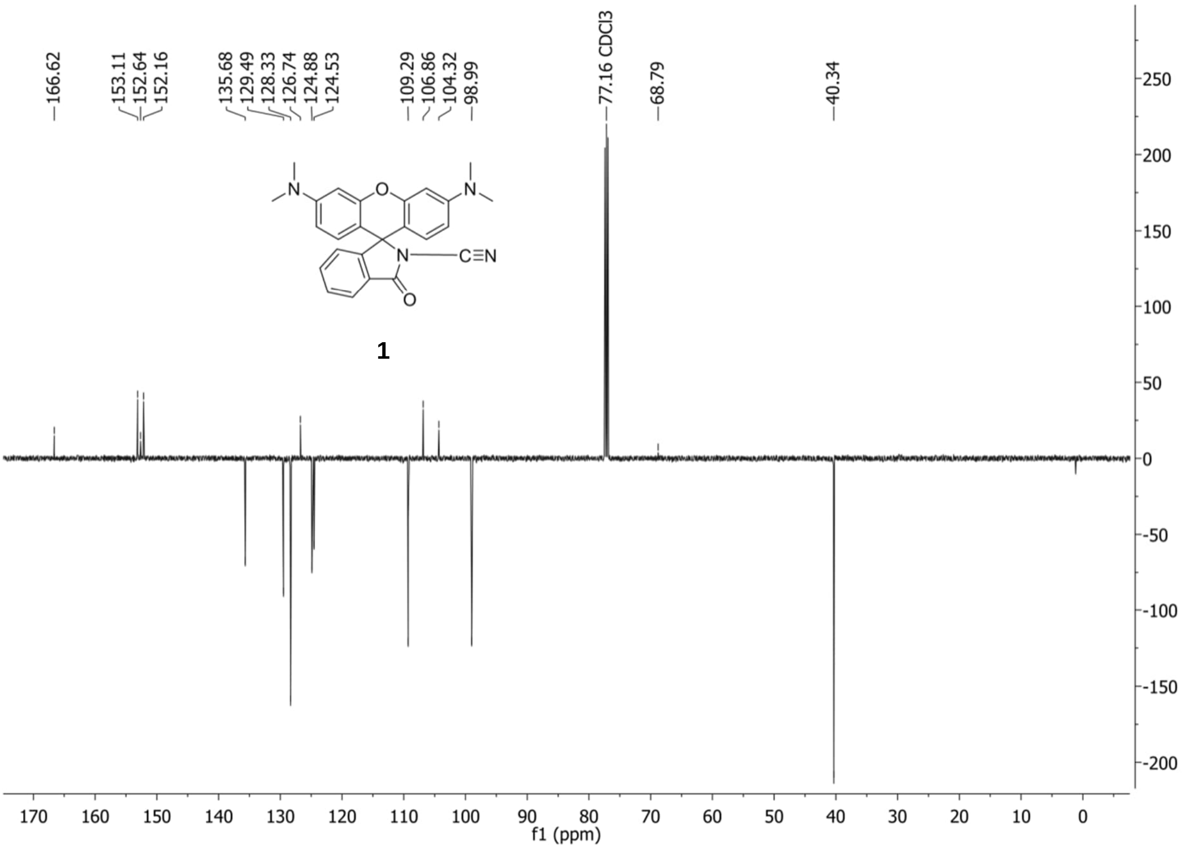
166.62
153.11
152.64
152.16
135.68
129.49
128.33
126.74
124.88
124.53
109.29
106.86
104.32
98.99
77.16 CDCl3
68.79
40.34
N
O
N
N
C≡N
O
1
f1 (ppm)

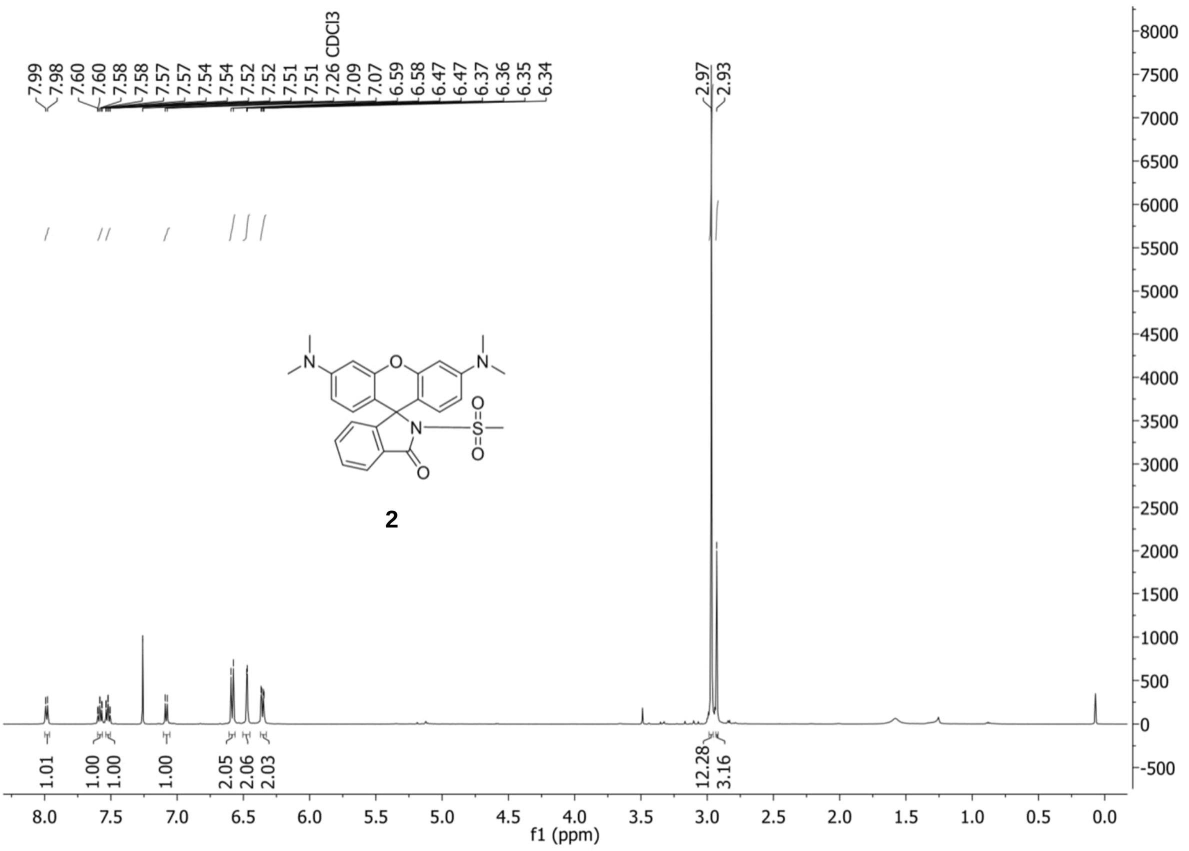
7.99
7.98
7.60
7.60
7.58
7.58
7.57
7.57
7.54
7.54
7.52
7.52
7.51
7.51
7.26 CDCl3
7.09
7.07
6.59
6.58
6.47
6.47
6.37
6.36
6.35
6.34
2.97
2.93
1.01
1.00
1.00
1.00
2.05
2.06
2.03
12.28
3.16
f1 (ppm)
2

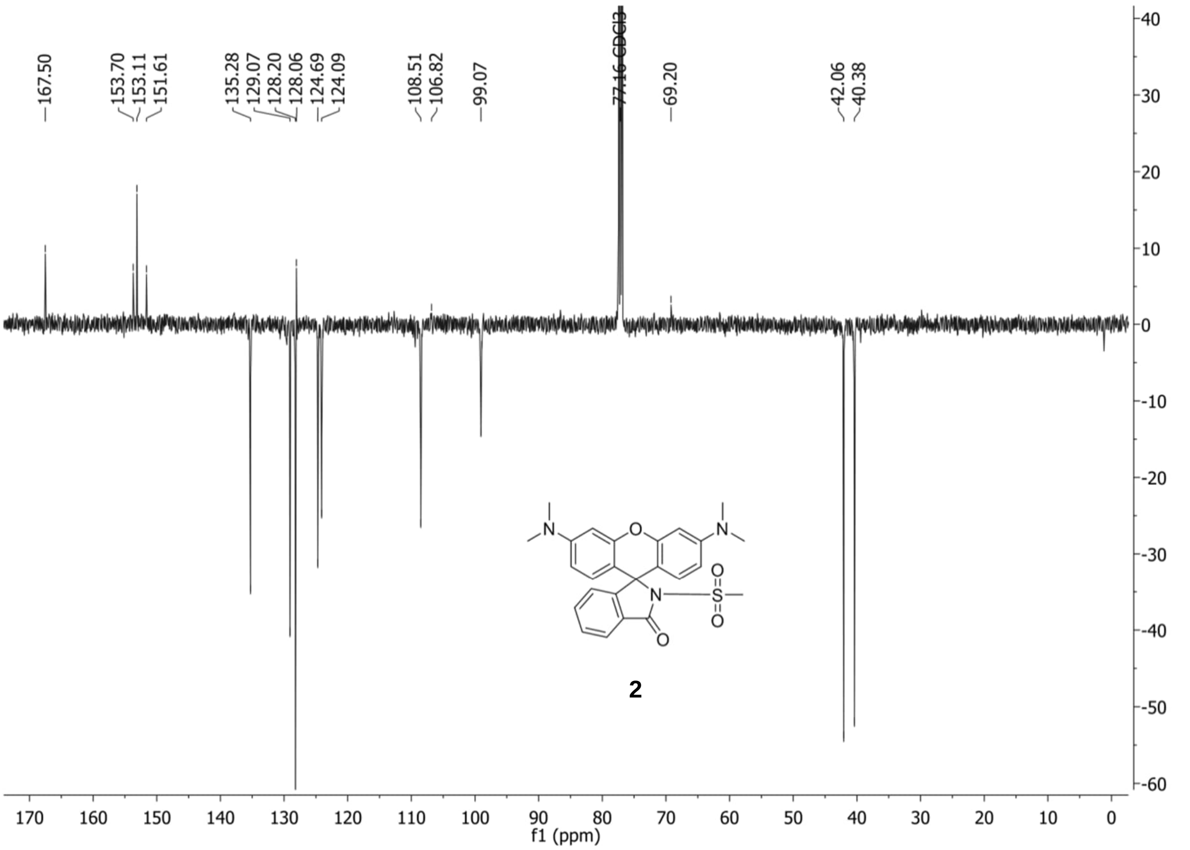
167.50
153.70
153.11
151.61
135.28
129.07
128.20
128.06
124.69
124.09
108.51
106.82
99.07
77.16 CDCl3
69.20
42.06
40.38
f1 (ppm)
2

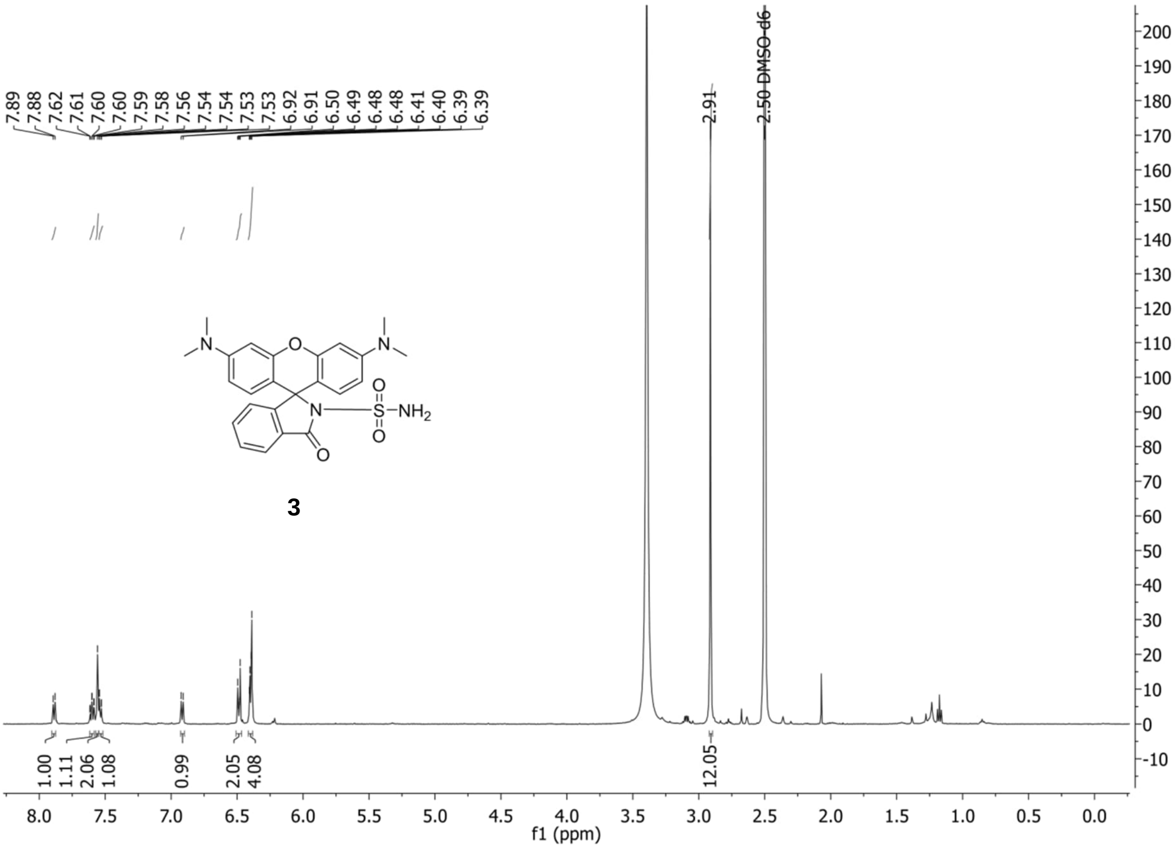
7.89
7.88
7.62
7.61
7.60
7.60
7.59
7.58
7.56
7.54
7.54
7.53
7.53
6.92
6.91
6.50
6.49
6.48
6.48
6.41
6.40
6.39
6.39
2.91
2.50 DMSO-d6
O
N
N
N
S
O
O
NH2
O
3
1.00
1.11
2.06
1.08
0.99
2.05
4.08
12.05
f1 (ppm)

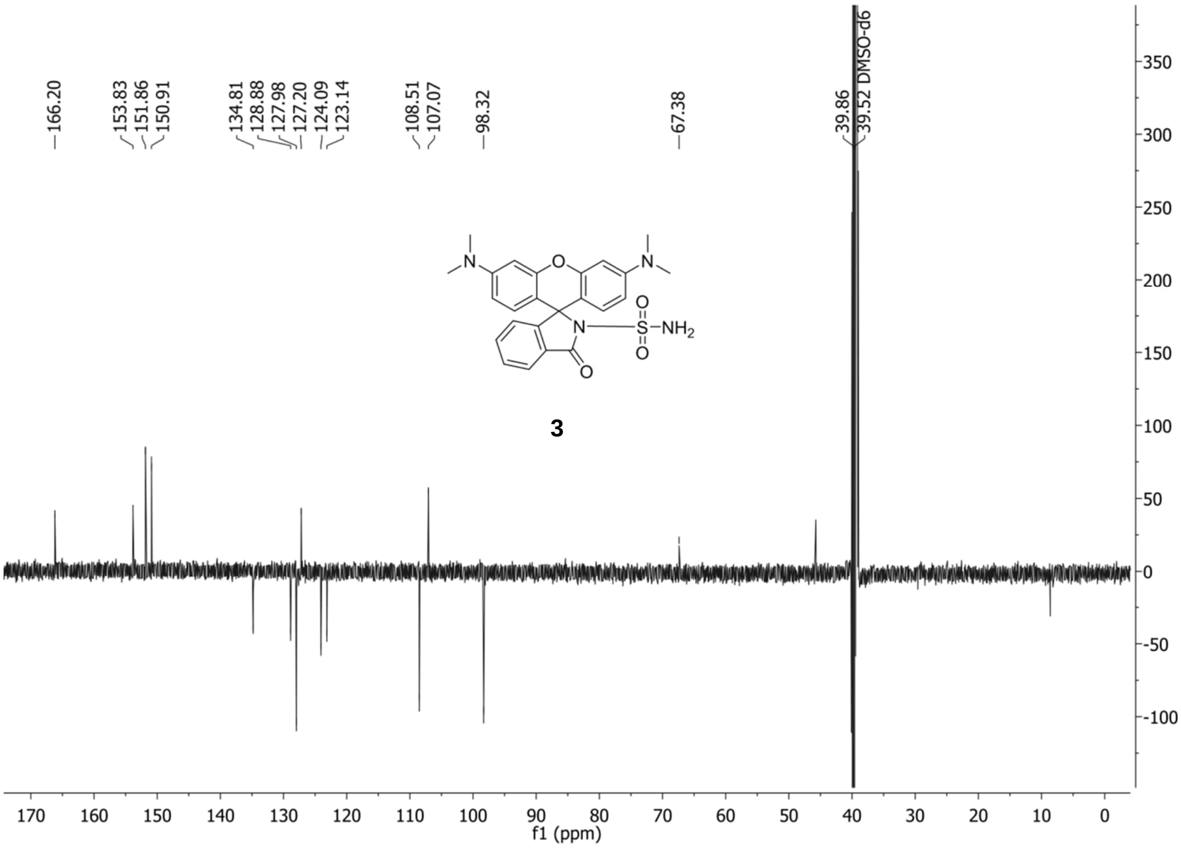
166.20
153.83
151.86
150.91
134.81
128.88
127.98
127.20
124.09
123.14
108.51
107.07
98.32
67.38
39.86
39.52 DMSO-d6
3
f1 (ppm)

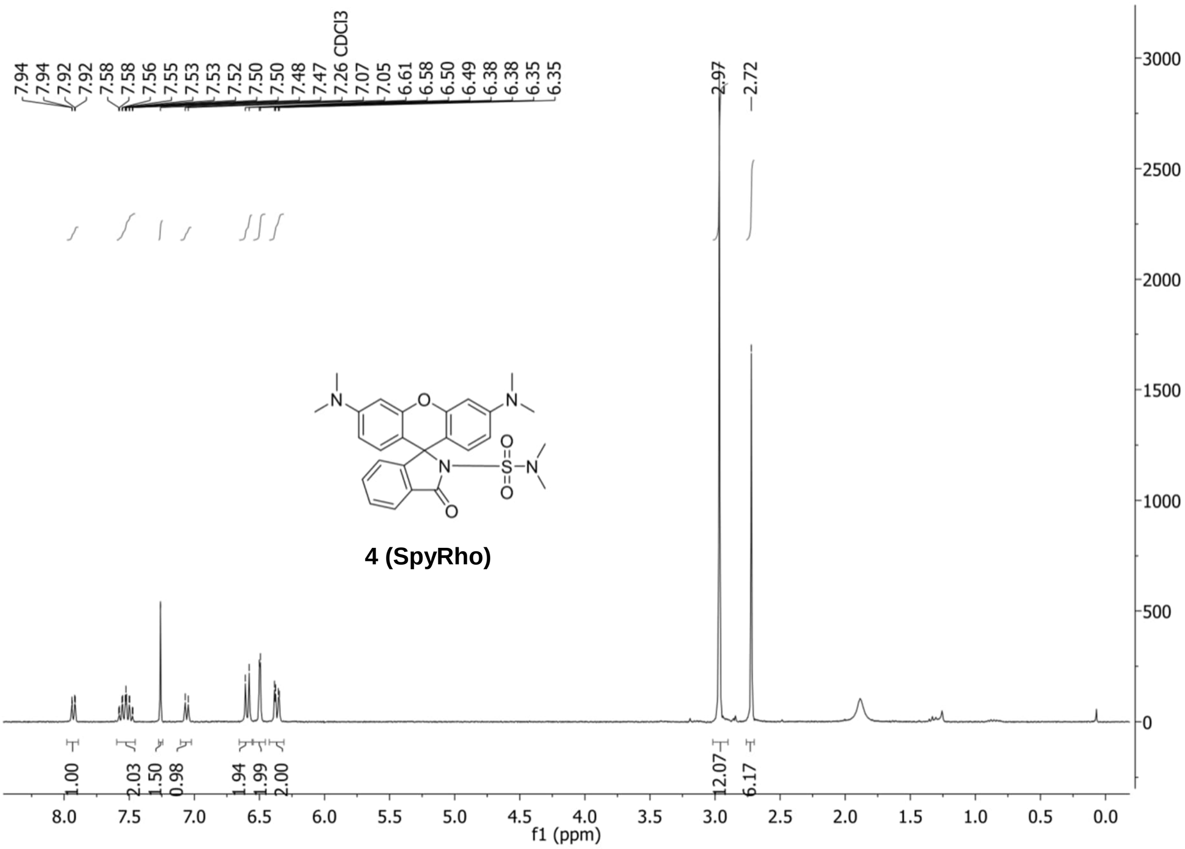
7.94
7.94
7.92
7.92
7.58
7.58
7.56
7.55
7.53
7.53
7.52
7.50
7.50
7.48
7.47
7.26 CDCl3
7.07
7.05
6.61
6.58
6.50
6.49
6.38
6.38
6.35
6.35
2.97
2.72
4 (SpyRho)
1.00
2.03
1.50
0.98
1.94
1.99
2.00
12.07
6.17
f1 (ppm)

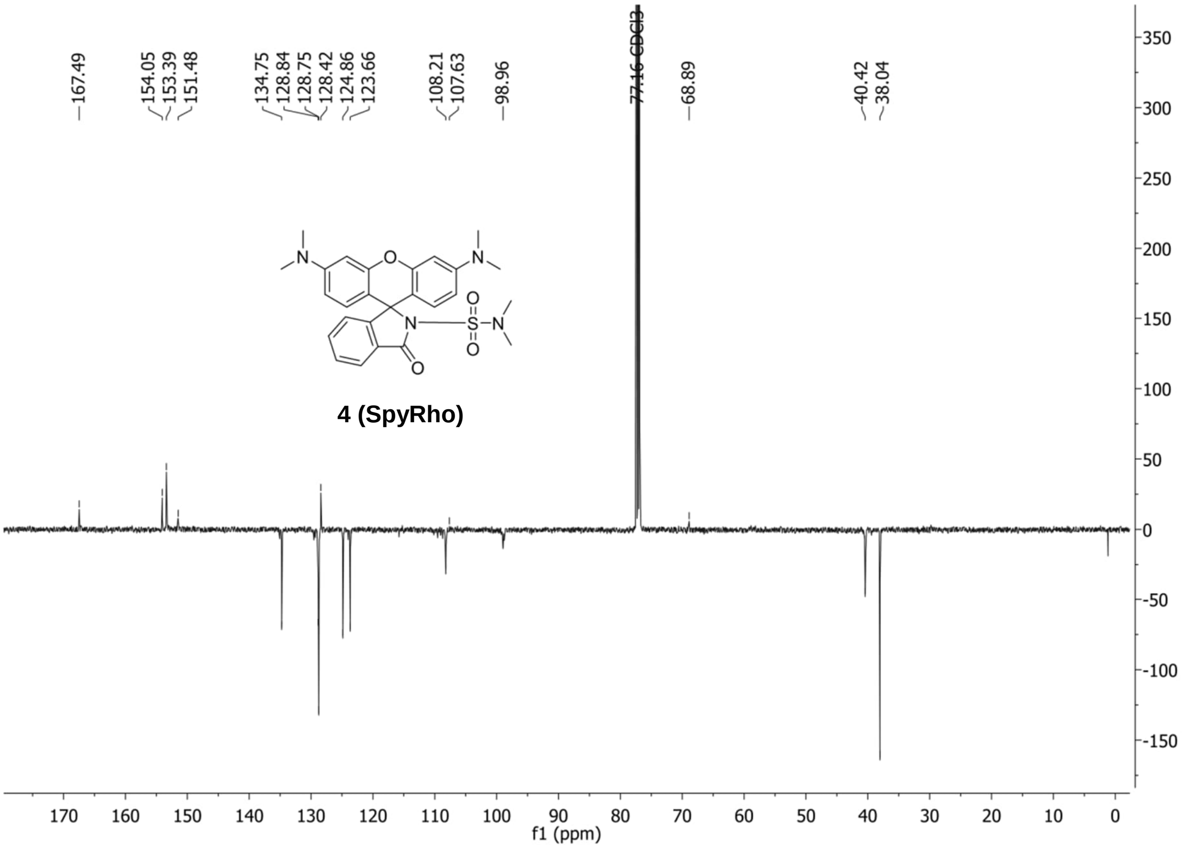
167.49
154.05
153.39
151.48
134.75
128.84
128.75
128.42
124.86
123.66
108.21
107.63
98.96
77.16 CDCl3
68.89
40.42
38.04
4 (SpyRho)
f1 (ppm)

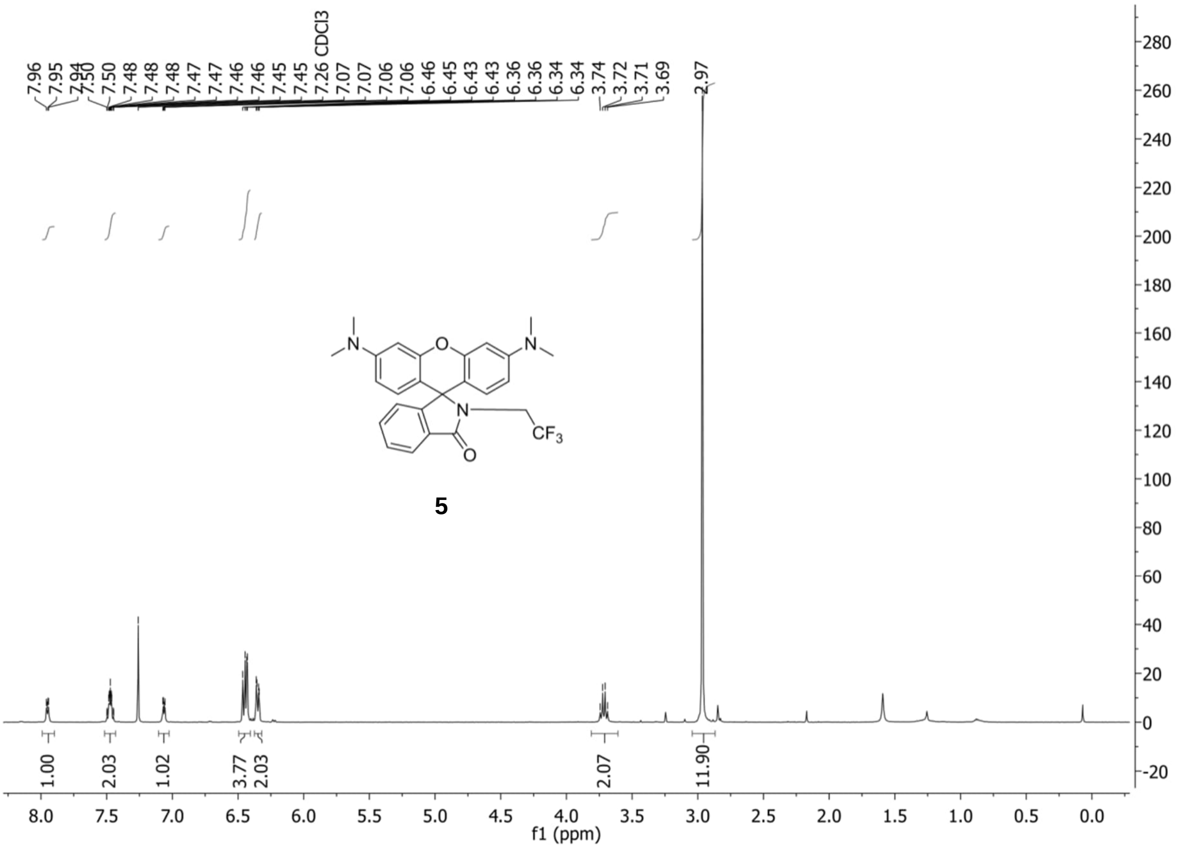
7.96
7.95
7.94
7.50
7.50
7.48
7.48
7.48
7.47
7.47
7.46
7.46
7.45
7.45
7.26 CDCl3
7.07
7.07
7.06
7.06
6.46
6.45
6.43
6.43
6.36
6.36
6.34
6.34
3.74
3.72
3.71
3.69
2.97
CF3
O
N
5
1.00
2.03
1.02
3.77
2.03
2.07
11.90
f1 (ppm)

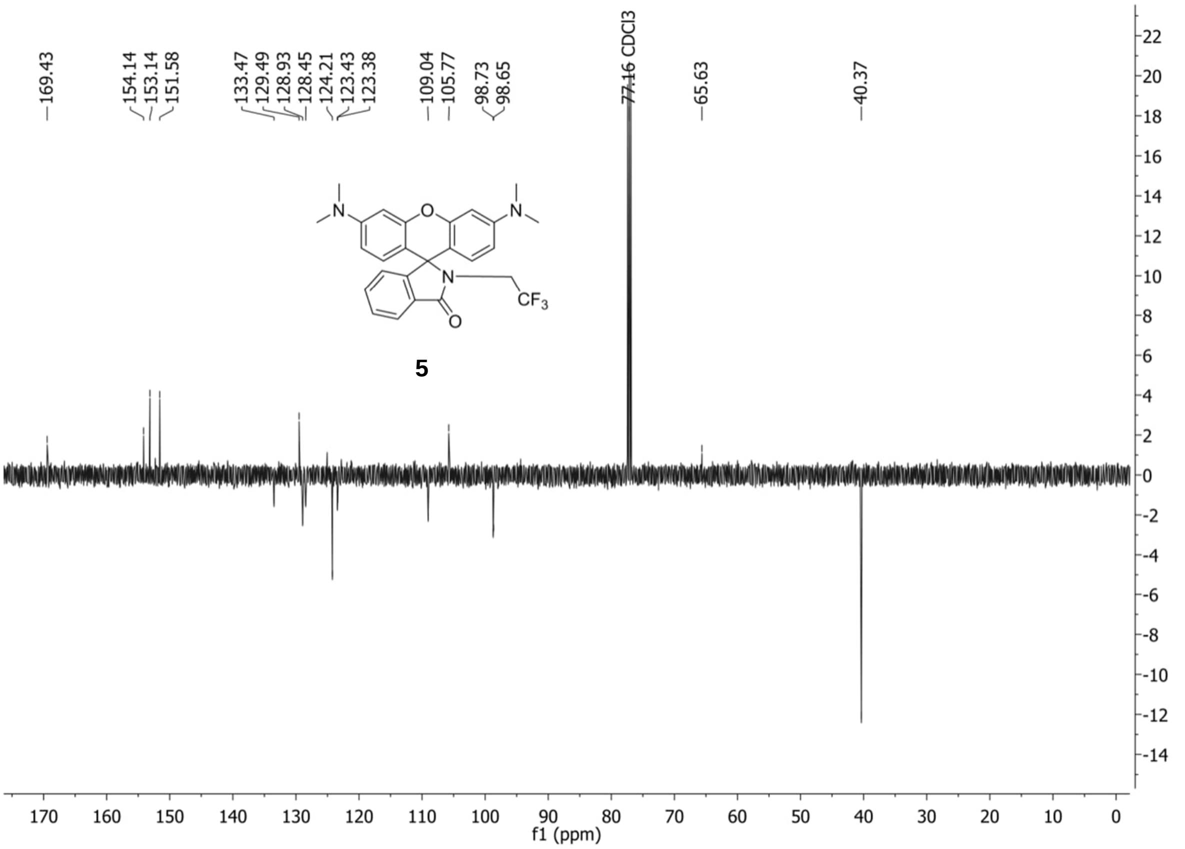
169.43
154.14
153.14
151.58
133.47
129.49
128.93
128.45
124.21
123.43
123.38
109.04
105.77
98.73
98.65
77.16 CDCl3
65.63
40.37
CF3
O
N
5
f1 (ppm)

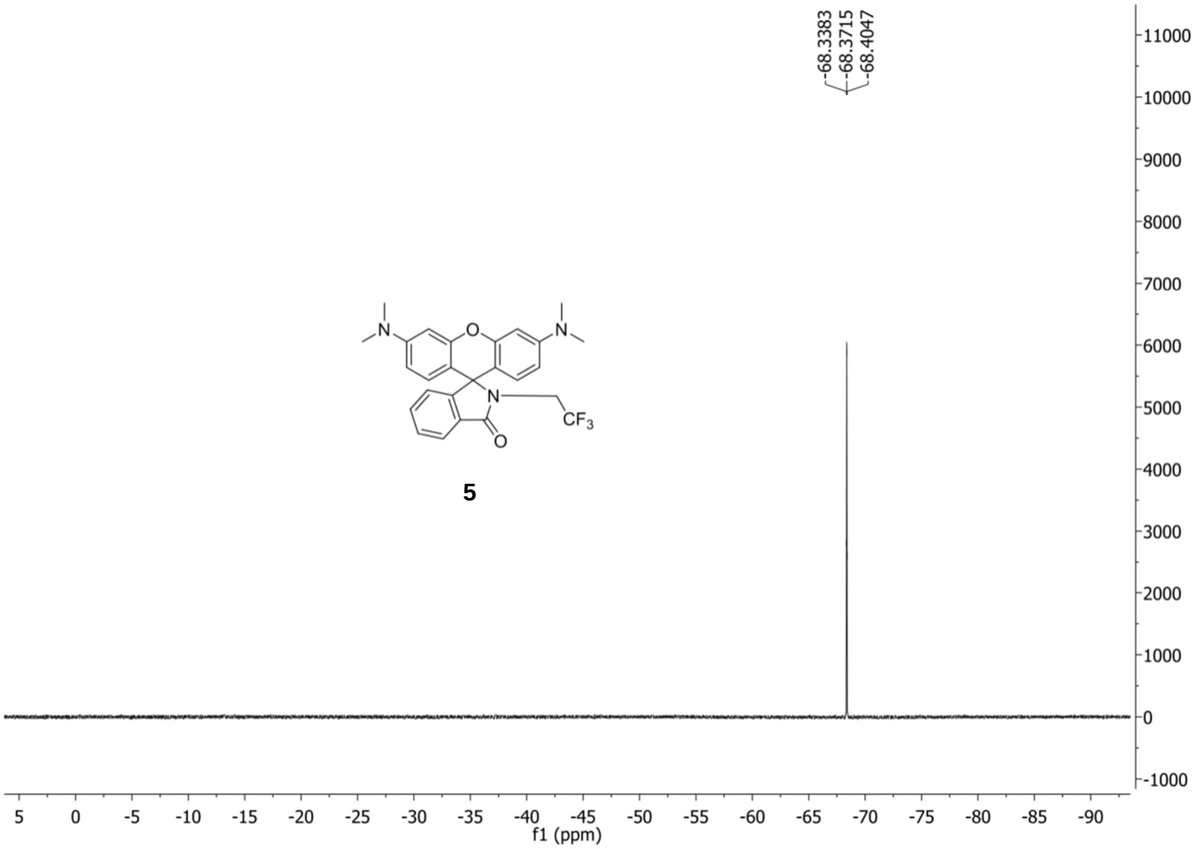
-68.3383
-68.3715
-68.4047
5
f1 (ppm)

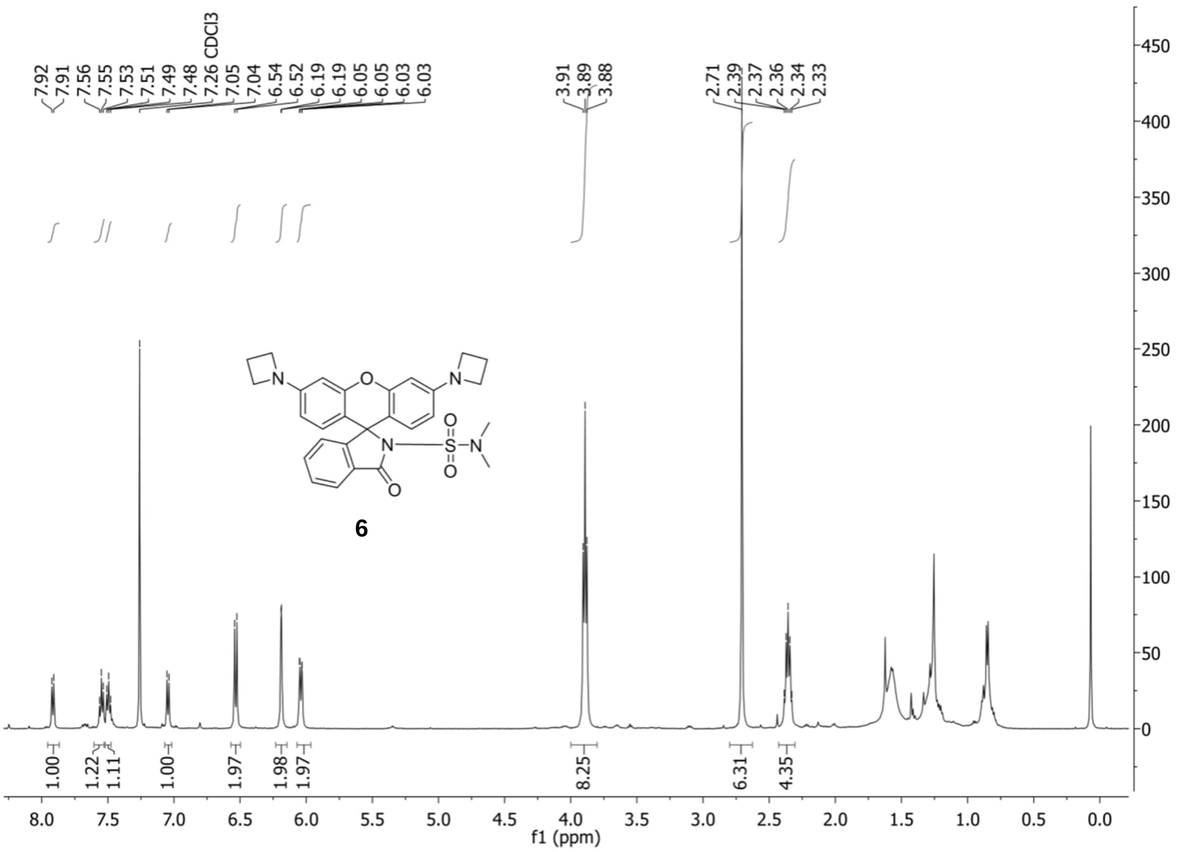
7.92
7.91
7.56
7.55
7.53
7.51
7.49
7.48
7.26 CDCl3
7.05
7.04
6.54
6.52
6.19
6.19
6.05
6.05
6.03
6.03
3.91
3.89
3.88
2.71
2.39
2.37
2.36
2.34
2.33
1.00
1.22
1.11
1.00
1.97
1.98
1.97
8.25
6.31
4.35
6
f1 (ppm)

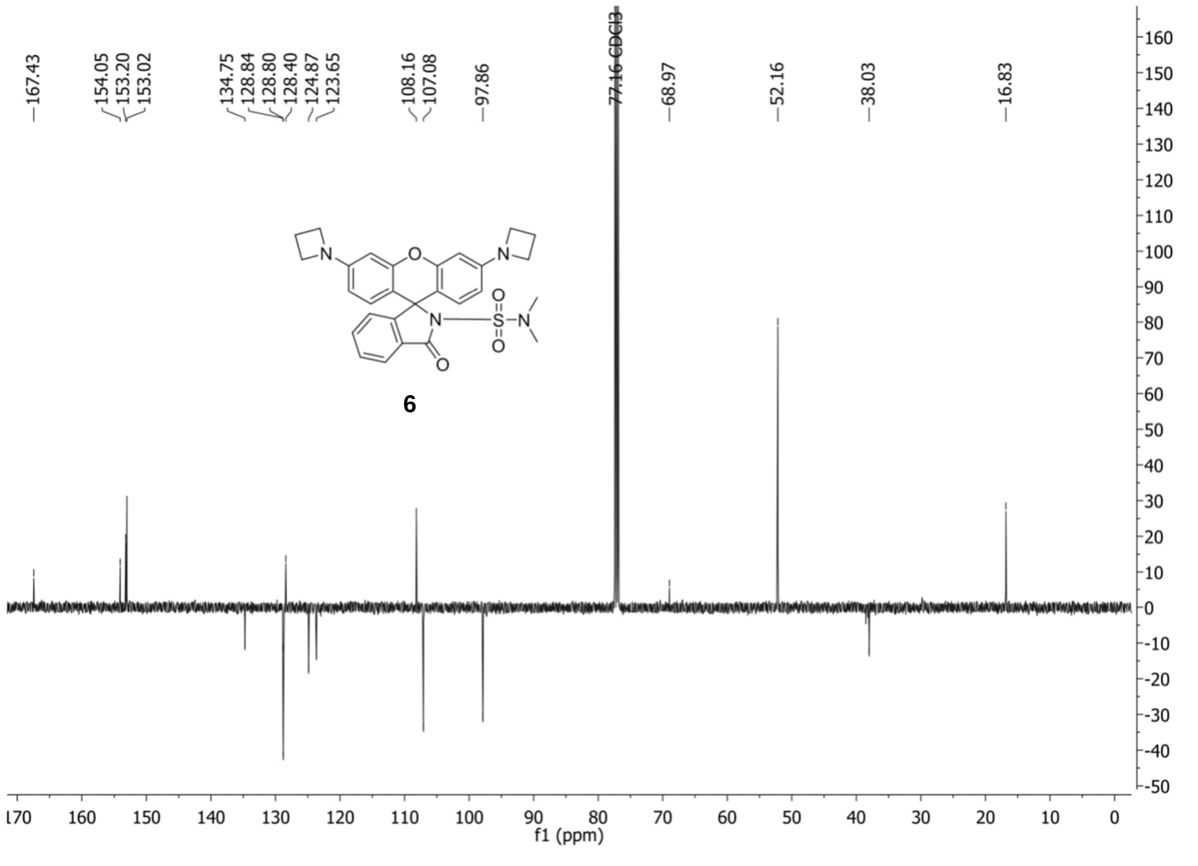
167.43
154.05
153.20
153.02
134.75
128.84
128.80
128.40
124.87
123.65
108.16
107.08
97.86
77.16 CDCl3
68.97
52.16
38.03
16.83
6
f1 (ppm)

Supplementary References

1. Sunbul, M. & Jäschke, A. SRB-2: a promiscuous rainbow aptamer for live-cell RNA imaging. *Nucleic Acids Res.* (2018).
2. Grimm, J.B. et al. A general method to improve fluorophores for live-cell and single-molecule microscopy. *Nat. Methods* 12, 244-250 (2015).
3. Grimm, J.B. et al. A general method to fine-tune fluorophores for live-cell and in vivo imaging. *Nat. Methods* 14, 987-994 (2017).
4. Mitronova, G.Y. et al. New fluorinated rhodamines for optical microscopy and nanoscopy. *Chem. Eur. J.* 16, 4477-4488 (2010).
5. Grimm, F., Nizamov, S. & Belov, V.N. Green-Emitting Rhodamine Dyes for Vital Labeling of Cell Organelles Using STED Super-Resolution Microscopy. *ChemBioChem* 20, 2248-2254 (2019).
6. Butkevich, A.N. et al. Fluorescent rhodamines and fluorogenic carbopyronines for super-resolution STED microscopy in living cells. *Angew. Chem. Int. Ed.* 55, 3290-3294 (2016).
7. Zheng, Q. et al. Rational Design of Fluorogenic and Spontaneously Blinking Labels for Super-Resolution Imaging. *ACS Cent. Sci.* 5, 1602-1613 (2019).
8. Lee, M. & Grissom, C.B. Design, Synthesis, and Characterization of Fluorescent Cobalamin Analogues with High Quantum Efficiencies. *Org. Lett.* 11, 2499-2502 (2009).
9. Schulte-Zweckel, J. et al. Site-specific, reversible and fluorescent immobilization of proteins on CrAsH-modified surfaces for microarray analytics. *Chem. Commun.* 50, 12761-12764 (2014).
10. Arora, A., Sunbul, M. & Jäschke, A. Dual-colour imaging of RNAs using quencher- and fluorophore-binding aptamers. *Nucleic Acids Res.* 43, e144 (2015).
11. Niwa, M., Hirayama, T., Okuda, K. & Nagasawa, H. A new class of high-contrast Fe(ii) selective fluorescent probes based on spirocyclized scaffolds for visualization of intracellular labile iron delivered by transferrin. *Org. Biomol. Chem.* 12, 6590-6597 (2014).
12. Grimm, J.B. & Lavis, L.D. Synthesis of Rhodamines from Fluoresceins Using Pd-Catalyzed C–N Cross-Coupling. *Org. Lett.* 13, 6354-6357 (2011).
13. Grimm, J.B. et al. A general method to improve fluorophores for live-cell and single-molecule microscopy. *Nat. Methods* 12, 244-250 (2015).
