# Supplementary material for: Fast-exchanging spirocyclic rhodamine probes for aptamer-based super-resolution RNA imaging
Source: Nat Commun. 2023 Jun 30;14:3879. doi: 10.1038/s41467-023-39611-1 (PMC10313827; doi:10.1038/s41467-023-39611-1)
Supplement: Supplementary file 4 — Description of Additional Supplementary Files [file 41467_2023_39611_MOESM4_ESM.pdf]

Tile: Supplementary Movie 1

Description: Fluorescence blinking of RhoBAST:SpyRho during SMLM experiments
